# Removal efficiency of pesticide residues on pesticide-spiked Perilla Leaf and Broccoli surfaces using microplasma-treated water

**DOI:** 10.1371/journal.pone.0351955

**Published:** 2026-06-26

**Authors:** Muhammad Saiful Islam Khan, Yun-Ji Kim, Jae-Hwan Ahn

**Affiliations:** 1 Department of Biomedicine and Life Science, School of Medicine, Central Asian University, Tashkent, Uzbekistan; 2 Korea Food Research Institute, Iseo-myeon, Wanju-gun, Jeollabuk-do, Republic of Korea; 3 Department of Food Biotechnology, Korea University of Science and Technology, Gajeong-ro, Yuseong-gu, Daejeon, Republic of Korea; Central Food Technological Research Institute CSIR, INDIA

## Abstract

In the current study, we evaluated the applicability of a microplasma device to reduce pesticide residues from the surface of perilla leaf and broccoli. We compared the pesticide removal efficiencies of four different washing methods: soaking in water, bubbling water, microplasma- treated water, and chlorine water. Pesticide-spiked food produce surfaces were treated individually with 2 mL of 2000 ppm of the pesticide solutions diazinon and chlorpyrifos. Washing water treated with microplasma was applied in two different ways, i.e., in bubbling and aerosolized modes. The removal efficiency of pesticides from the produce surface was determined by HPLC analysis following 4 min of treatment. Washing with microplasma-treated water (both under water and aerosolized modes) and chlorine water removed **80.84–87.17%** of pesticides from perilla leaves and **51.74–67.77%** from broccoli, irrespective of pesticide type. Evaluation of the effective washing systems at different temperatures showed that reducing the temperature from 22°C to 10°C resulted in greater pesticide removal and/or degradation in the case of washing with microplasma-treated water; however, chlorine water washing showed a reverse trend. No significant color differences were observed for any of the washing treatments (p > 0.05), even after one week of refrigerated storage.

## Introduction

Fresh produce is the most important item for a healthy diet plan. Recent national assessments by the Korean Ministry of Agriculture, Food and Rural Affairs (MAFRA) as well as the Korea National Health and Nutrition Examination Survey (KNHANES) show a continued rise in fruit and vegetable consumption in Korea [1,2]. KNHANES 2022 reports that over 72% of adults consume fresh vegetables regularly, which aligns with the **Korean Dietary Guidelines issued by the Ministry of Health and Welfare** promoting increased vegetable intake [1]. MAFRA’s 2023 trends further pointed to growing demand for fresh, nominally processed food driven by increasing health consciousness [2,3]. The growing demand for fresh vegetables indicates that the use of pesticides in farming all over the world is expected to increase at a similar speed [4]. Pesticides are chemicals used to raise the yields of crops by regulating pests and growing crop yield, nutritional value and appearance, ensuing in improved global trade [5–7]. Worldwide monitoring programs (e.g., EFSA, FDA) constantly report detectable pesticide residues on fresh produce, with some exceeding regulatory limits [8,9]. Current studies also highlight potential health risks related with chronic low-level exposure [10]. These comprise recent global assessments and epidemiological studies signifying relations between pesticide exposure and adverse health outcomes such as endocrine disruption, neurotoxicity, and chronic disease risks [10–12]. Organophosphorus pesticides (OPPs) were introduced as substitutes to persistent organochlorine pesticides primarily because of their reduced environmental persistence and lower tendency to bioaccumulate. OPPs exhibit moderate polarity and low-to-moderate water solubility, which differs extensively among individual compounds. However, the widespread agricultural use of organophosphate pesticides (OPPs) has become a significant contributor to surface water pollution, making OPPs a major concern for public health, food safety, and environmental sustainability [13,14]. While pesticides play a vital role in preserving crop productivity, their application frequently results in detectable residues on fresh produce, thereby raising persistent food safety and consumer health concerns. These alarms emphasize the need for effective and safer residue-reduction methods. Hence, several washing systems have been considered to remove chemical and biological threats from fresh produce [7].

Conventional fresh produce sanitization procedures, such as washing with tap water, chlorinated solutions, ozone, or organic acids, display notable limitations, including insufficient elimination of strongly attached pesticide residues and pathogenic microbes [15–17]. Furthermore, chlorine-based treatments may produce detrimental by-products, ozone and organic acids are expensive, limited penetration efficiency, or adverse effects on the quality of foodstuffs [16,18]. The effectiveness of these methods is additionally influenced by surface roughness and moisture content, leading to unreliable sanitization performances [17]. These shortcomings underscore the urgent need for alternative decontamination strategies that are effective, residue-free, eco-friendly, and safe for both consumers and food handlers [19]. Research is ongoing to achieve effective pesticide reduction from the food surface without changing the physicochemical characteristics of the treated produce items [20]. In this context, plasma-based and cutting-edge oxidation technologies have emerged as promising substitutes due to their ability to produce highly reactive species capable of attaining rapid and broad-range sanitization without chemical residues. For example, a physical processing technique known as nonthermal plasma treatment is an effective, eco-friendly, and economical way to remove pesticide residues in fresh produce [21]. However, numerous plasmas-generating techniques such as pulsed high-current discharge, gliding arc discharge, corona discharge, and capillary discharge can generate different types of active species during the process of inactivating different types of chemical and microbial threats [22]. Reactive species such as reactive oxygen species (ROS), reactive nitrogen species (RNS), and UV light are produced with various kinds of electrical discharge [23].

The use of microplasma-treated water is an emerging technology used for pesticide removal and/or degradation in fresh produce. Microplasma-treated water (MPW) is generated by discharging low-power microplasma directly into water, producing reactive oxygen and nitrogen species (RONS), including •OH, O₂ ⁻ , NO₂ ⁻ , and NO₃ ⁻ , within the liquid phase. Unlike conventional plasma jets that primarily treat produce surfaces, MPW enables uniform chemical interactions during washing, operates at lower energy input, and offers better scalability for practical decontamination applications [24,25]. Our previous work demonstrated that microplasma treatment of water for 1–3 min resulted in 97–99% degradation of chlorpyrifos and diazinon in standard aqueous solutions, with a standard deviation of less than ±2% [26]. Building on these findings, the present study moves beyond solution-based systems and evaluates the effectiveness of MPW washing in comparison with conventional chlorine washing, for removing and/or degrading these pesticides from actual food surfaces, namely broccoli and perilla leaves. Although pesticide residue levels on fresh produce under field conditions are typically much lower, a higher initial stock solution concentration (2000 ppm) was used to ensure accurate spiking and analytical reliability. The final residue levels in the samples (0.1–0.5 µg/g) were adjusted according to SANTE/11312/2021 guidelines, thereby providing realistic and comparable conditions for removal and/or degradation assessment [27]. It should be noted that the high stock solution concentration does not reflect actual residue levels on produce; rather, it facilitates accurate spiking, while the final concentrations applied to the samples (0.1–0.5 µg/g) are within ranges reported for real-world conditions and aligned with regulatory guidelines.

## Materials and methods

### Produce sample preparation and pesticide contamination

Perilla leaves (*Perilla frutescens*) and broccoli (*Brassica oleracea* var. *italica*) were selected as typical leafy and cruciferous vegetables for this study. Organically sourced produce was procured from a local market in Jeonju, South Korea. Perilla leaves were cut into standardized 2 × 2-inch sections (20 pcs, approx. 3.0 g), and broccoli florets were trimmed into ~1 cubic inch pieces (20 pcs, approx. 200 g) to ensure uniform sample size and surface area. Each sample was pesticide-spiked by dispensing 2 mL of the pesticide solution (diazinon or chlorpyrifos, applied separately) using a calibrated micropipette and distributing it evenly across the surface to ensure uniform exposure. Following application, the samples were air-dried for 2 h under controlled laboratory conditions (22–24 °C, 45–55% relative humidity) in a fume hood with gentle, constant airflow (≈ 0.3 m/s). A 2000 ppm stock solution was used to prepare appropriate intermediate working solutions, and aliquots of these were applied to achieve the desired spiking levels in the samples. The final pesticide concentrations in the matrices (0.1 and 0.5 µg/g) were established based on sample weight in accordance with SANTE/11312/2021 guidelines. Unless otherwise stated, a total application volume of 2 mL was maintained for all treatments to ensure consistent surface coverage. Samples washed with untreated water were used as controls to distinguish simple washing effects from plasma-assisted degradation. Three independent treatments of each sample were performed prior to HPLC analysis to ensure reproducibility and statistical reliability of the results.

### Generation of microplasma-treated water

**Fig 1:**
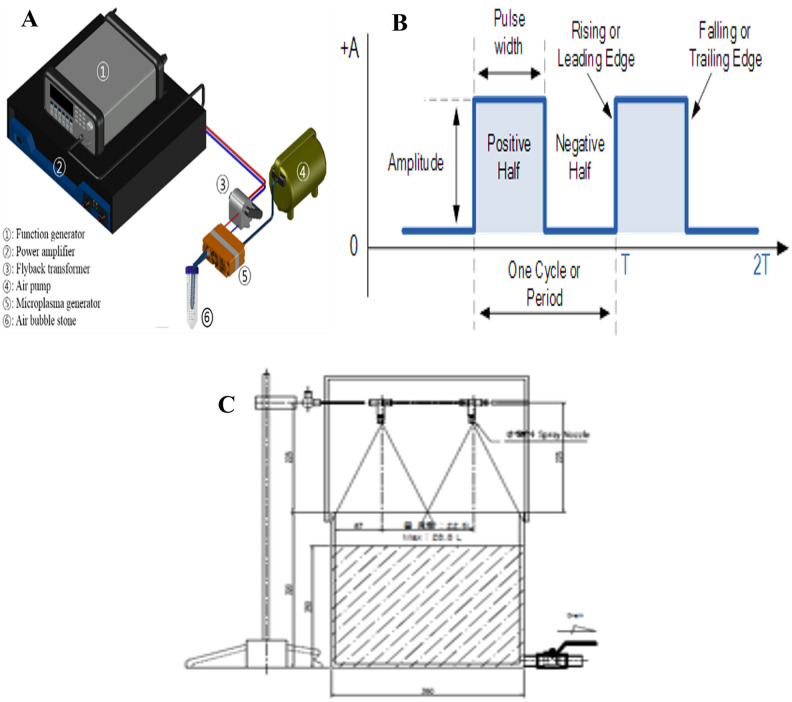
(A) Figure of the microplasma generation scheme; (B) square wave motion from the waveform generator; (C) Figure of the aerosol production system.

**Fig 2:**
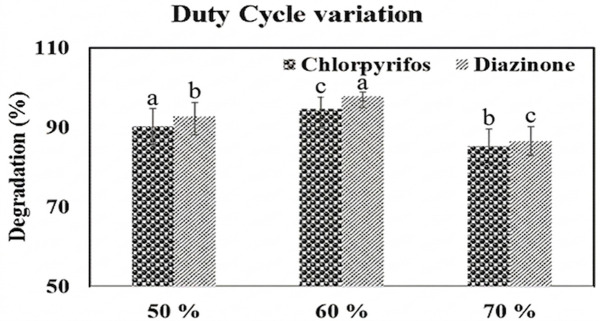
Effect of duty cycle variation on the degradation efficiency of Chlorpyrifos and Diazinon in aqueous solution at a constant temperature of 22 ± 1°C. Different lowercase letters indicate significant differences between treatments (p < 0.05).

**Fig 3:**
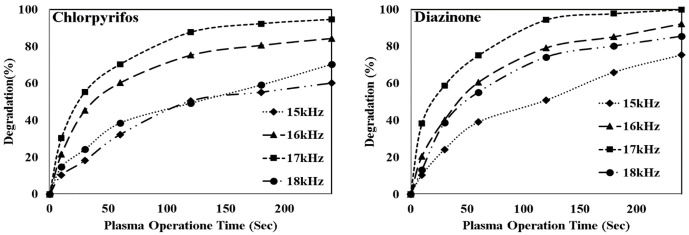
Pesticide removal at different working frequencies. The temperature of the water solution remained constant at 22 ± 1°C during the entire treatment.

The microplasma generator, originally developed at the University of Illinois, USA, has been described earlier [24,25]. In the present study, the same generator design as reported in our earlier work [28] was employed; and the operating conditions were further optimized for the current experiments. Fig 1A displays the microplasma generation system. The device was powered using a function generator (33220A, Agilent Technologies, USA), a power amplifier (GX-7, QSC LLC, USA), and a high-voltage boost converter (FLYLABURN, Information Unlimited, USA). A quadrangular waveform (10 Vp-p) was amplified to 35 V using a current stimulator, after which the signal was sent to the converter (Fig 1B). The transformer output voltage was about 30 kV, with operating frequencies ranging from 15 to 18 kHz and a duty cycle of 20–80%. Based on optimization trials, the optimal operating conditions were identified as a duty cycle of 60% (Fig 2) and an operating frequency of 17 kHz (Fig 3). Atmospheric air served as the feed gas. Energy consumption was monitored using a wattmeter (HPM-300A, AD Power Co. Ltd., Korea), yielding an average reading of 153.7 ± 0.57 watts (W). The voltage waveform was recorded with an oscilloscope. The generated plasma stream was introduced into water using a circular bubble diffuser, allowing homogeneous distribution of microplasma jets through the treatment container. A cooling system (Part 5 in Fig 1A), placed near the electrode, maintained the temperature of the water in plastic jar at about **22 ± 1°C** throughout the treatment. The aerosolized system was constructed within a plastic cabinet. Microplasma-treated water (MPW) was sprayed from the top of the cabinet using a nozzle with a **0.1 μm orifice size**, facilitating uniform aerosol generation (Fig 1C).

### Optical emission spectroscopy

Reactive species generated by the microplasma were analyzed using optical emission spectroscopy (OES). End-on emission from the discharge was collected via a fiber-optic cable and directed to a 0.75 m spectrophotometer (Acton SpectraPro 2750, Princeton Instruments, NJ, USA) equipped with an 1800-groove/mm holographic grating. Spectra were recorded over the 200–1100 nm range with a 100 μm entrance slit, yielding a spectral resolution of approximately Δλ ≈ 0.06 nm. An intensified charge-coupled device (ICCD; I-Max-512, Princeton Instruments) was used with integration times of 200–1100 ms, and each spectrum was averaged over 10 accumulations. Emission lines corresponding to major reactive species (e.g., OH, NO, N₂, and O) were identified using the NIST Atomic Spectra Database and reported plasma spectroscopy literature [28]. Representative spectra are provided in the Supplementary Information (S1 Fig).

### Determination of radical concentrations

Microplasma generates dissolved ozone, which we measured using a UV O_3_ display (Model 620, Ebara Jitsugyo, Japan). MPW was passed through the O_3_ measuring device through a plastic pipe connected to the lower part of the microplasma system. The concentration of hydroxyl radicals (•OH) generated in the treated water was estimated using terephthalic acid as a selective •OH scavenger [28]. Very short-lived OH radicals can combine and form hydrogen peroxide. Therefore, we used a commercially available H_2_O_2_ measuring kit (Amplex^®^, Molecular Probes, Eugene, OR, USA) to find the amount of hydrogen peroxide produced. A nitric oxide assay kit (QuantiChrom, BioAssay Systems, Hayward, CA, USA) was used to determine the levels of oxides of nitrogen (mostly nitrate and nitrite) that were produced in the microplasma-treated water. The pH of the microplasma-treated water was measured using an electronic pH meter (Thermo Scientific™ Orion™ Versa Star Pro™ Benchtop Meter). Comprehensive details of the identified hydroxyl radicals, oxides of nitrogen and hydrogen peroxide are provided in the Supplementary Information (1S- 3S text).

### Different modes of washing

All produce samples spiked with diazinon and chlorpyrifos were subjected to a 4 min treatment using four different approaches. In the soaking treatment, samples were immersed in distilled water without agitation to simulate conventional household washing. For the bubbling treatment, samples were placed in water through which air was continuously bubbled to provide mechanical agitation and enhanced mass transfer. In the MPW, plasma-activated water was applied in two modes: (i) an underwater mode, in which samples were directly submerged in MPW, allowing intimate contact with reactive species, and (ii) an aerosolized mode, in which MPW was sprayed as fine droplets onto the sample surface to simulate a spray-washing process. For chlorine water treatment, samples were immersed in a commercially available sodium hypochlorite–based disinfectant (Yuhanrox®, Yuhan Corporation, Seoul, Republic of Korea), diluted with distilled water to obtain a free chlorine concentration of about 200 ppm and treated for 4 min at room temperature. The solution pH was maintained at **pH 6.5–7.0** to ensure optimal chlorine activity. After treatment, samples were removed and rinsed with **distilled water** to eliminate residual chlorine, followed by air drying under ambient conditions. The treated samples were then subjected to pesticide residue analysis as described in below. All experiments were conducted in triplicate. All treatments were done for an equal duration of 4 min to allow direct comparison of treatment efficiency. Based on our previous studies, near-complete degradation (~99%) of diazinon and chlorpyrifos in standard solutions occurs within 3 min of direct microplasma treatment. The 4-min exposure exceeds the degradation time observed in standard solutions; however, degradation of pesticides transferred to the wash water was not directly verified under the present experimental conditions. Ultrasonication may allow limited additional degradation due to residual reactive species, although this effect is expected to be minimal.

### Pesticide extraction

After each treatment, samples were immediately transferred to 50-mL centrifuge tubes to minimize any post-treatment reactions. Twenty milliliters of methanol were added to the samples to rapidly quench reactive species and extract residual pesticides; for every experiment, the addition of methanol was performed quickly and at a consistent time interval to ensure a fair evaluation. The resulting mixture was vortexed and sonicated for 15 min to facilitate desorption of pesticides from the matrix. Ultrasonication was performed using a bath sonicator at 40 kHz and 150 W, with the temperature maintained at 22 ± 1°C to avoid thermal or secondary degradation effects. The samples were then centrifuged at 4000 rpm for 5 min, and the supernatant was filtered through a 0.22 µm membrane prior to analysis. Despite controlled extraction conditions, the possibility of continued degradation due to residual reactive species during ultrasonication cannot be completely excluded.

### Preparation of stock solutions and analytical calibration methods

The surfaces of the produce were individually treated with 2 mL of pesticide solution (diazinon or chlorpyrifos). A 2000 ppm stock solution was prepared by accurately weighing the appropriate mass of each standard (purity ≥99%, Sigma-Aldrich; diazinon, Cat. No. D-4401; chlorpyrifos, Cat. No. C-2430) using an analytical balance (±0.01 mg) and dissolved in HPLC-grade methanol using a calibrated volumetric flask. The stock solutions were sonicated for 5 min and filtered through a 0.22-µm PTFE syringe filter prior to use. All standards were stored in amber glass vials with PTFE-lined caps at −20 °C to minimize degradation. Working standards were freshly prepared by serial dilution in methanol and appropriate aliquots were used for sample spiking to achieve the desired concentrations in the samples. A consistent application volume of 2 mL was maintained to ensure uniform surface coverage. Working standards were stored at 4 °C for up to 7 days, while long-term aliquots were maintained at −20 °C. Stability was confirmed by comparing peak areas of freshly prepared and stored standards (acceptance: ± 10%). Calibration curves were constructed using at least six concentration levels (0.01–50 ppm) prepared by serial dilution of the stock solution. Each level was analyzed in triplicate. Excellent linearity was achieved, with correlation coefficients (R²) ranging from 0.997–0.999. Limits of detection (LOD) and quantification (LOQ), determined based on signal-to-noise ratios of 3 and 10, were 0.001–0.05 ppm and 0.003–0.01 ppm, respectively. Method accuracy and precision were evaluated through recovery studies at two spiking levels (0.1 and 0.5 µg/g). The mean recoveries ranged from 93.6% to 97.8%, with relative standard deviation (RSD) values ≤ 3.32%, demonstrating good accuracy and repeatability. These results met the acceptable criteria for method validation and are summarized in S1 Table.

### Determination of the residual amounts of diazinon and chlorpyrifos using HPLC

The extracted pesticide was analyzed by HPLC using a Luna 5U-C18 (2) 100A column (250 mm × 4.5 mm, 5 μm) equipped with a Jasco quaternary gradient pump (pu-2089) and a Jasco UV-2077 4λ intelligent UV/Vis detector. An isocratic system was maintained to elute diazinon and chlorpyrifos with a mobile phase comprising acetonitrile: water (75:25). Fifty microliters (50 µL) of the extracted samples were injected for each analysis. A continuous, 1 mL/min, flow rate was kept during the whole separation process. Diazinon and chlorpyrifos were measured at 245 nm and 290 nm, respectively, corresponding to their maximum UV absorbance. Diazinon was eluted at a retention time of 8.3 min with a total run time of 10 min, whereas chlorpyrifos exhibited a retention time of 12.8 min with a total run time of 15 min under the same chromatographic conditions. To ensure analytical validity, appropriate procedural controls were included. Solvent-only blanks were analyzed to account for potential contamination or analyte loss during extraction and handling. In addition, untreated samples spiked with a known concentration of pesticides but not exposed to MPW were used as matrix controls to evaluate recovery and stability of the pesticide in the absence of treatment (S7 text).

### Analysis of color changes

We used a colorimeter (Minolta CM-700d spectrophotometer; Konica Minolta Opts., Inc., Osaka, Japan) to identify any color changes occurring due to the microplasma treatment. The color measurements were conducted using a spectrophotometer configured with d/8° geometry, D65 illuminant, and a 10° standard observer. Color change measurements were recorded immediately after treatment and seven days later for untreated samples, samples treated with microplasma under water, and samples treated with chlorine; aerosolized microplasma-treated samples were not included in this analysis. The device was standardized to white stone (L* = 93.52, a* = 0.32, b* = 0.33). The brightness of the sample was dependent on the value of L*-axis, with values ranging from 0 (black) to 100 (white); the greenness (−)/redness (+) was dependent on the value of a*-axis; and blueness (−)/yellowness (+) was dependent on the value of the b* axis. The changes in each group before and after plasma treatment are denoted as Δ. We calculated color changes using the equation: (ΔE* = [ΔL*^2^ + Δa*^2^ + Δb*^2^]^1/2^) (Hunt & Pointer, 2011) where L*, a*, and b* values were determined by placing the spectrophotometer in front of the sample [29]. The samples were evaluated using a consistent sampling protocol, with five replications taken to ensure measurement reliability.

### Statistical analysis

All data were analyzed using a three-factor factorial analysis of variance (ANOVA) with washing method, pesticide type, and temperature as fixed factors. The statistical model included main effects and their interactions. Prior to analysis, assumptions of normality and homogeneity of variance were verified using the Shapiro–Wilk and Levene’s tests, respectively. When significant effects were detected (p < 0.05), Tukey’s honestly significant difference (HSD) test was applied for multiple comparisons among treatment means. Each treatment was conducted with n = 3 independent biological replicates unless otherwise stated. Results are presented as mean ± standard deviation, and statistical significance was set at p < 0.05.

### Ethical statement

Ethical approval was not required for this study as it did not involve human participants, human data, or animal subjects. Food samples were purchased from local markets and vendors through standard commercial transactions. No personal or identifiable information was collected from vendors. Permission to conduct laboratory analyses was granted by the appropriate institutional authority of **Korea Food Research Institute**, **Republic of Korea** and all experimental procedures were performed in accordance with institutional research and safety guidelines.

## Results

### Current–voltage features of the microplasma device

The microplasma producing device and its different components are shown in Fig 1. Part 5 of Fig 1 shows a picture of the microplasma electrode, which we have described previously [26]. Fig 1B shows the applied voltage (found as a square wave) and the amplified current waveform the amplifier, respectively. The total current is the combination of displacement current and numerous voltage pulsations. Photoionization was achieved by filamentary micro discharges. The formation of ions in a plasma device depends on certain functional parameters for example, the discharge hole, frequency, voltage, and width of the electrode. We achieved steady ion generation by maintaining optimum working conditions as described in the materials and methods section above.

### Characteristics of reactive radicals produced by microplasma

**Fig 4:**
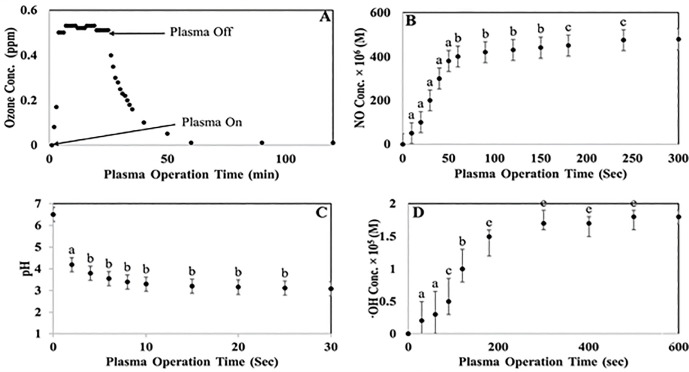
Changes in the levels of different species generated by microplasma treatment in water and their actions. **A) Generation of dissolved O**_**3**_ **and its disappearance after switching off the microplasma source. B) Amount of nitrogen oxides generated due to the microplasma operation. C) Changes in pH of the water during microplasma treatment. D) Changes in the amount of OH radical throughout microplasma action. (Ozone concentration showed minimal fluctuation during the 8-min treatment; error bars were therefore omitted because variability was within the instrument’s precision.) Data at longer treatment times are not shown because no significant additional changes occurred after the reactive species reached their maximum concentration.** For **(B)**, **(C)**, and **(D)**, different lowercase letters denote statistically significant differences between treatment intervals (p < 0.05).

Optical emission spectroscopy (OES) was performed using an optical emission spectrometer (HR4000, Ocean Optics Inc., USA). The spectrometer used a CCD detector, and the working range of the detector was 200–1100 nm. The OES spectrum in the near-UV region (300–400 nm) is dominated by emission peaks corresponding to OH radicals and the second positive system of N₂ (C–B), indicating the generation of reactive oxygen species and reactive nitrogen species during microplasma discharge. In addition to OH radicals and reactive nitrogen species (RNS), H_2_O_2_ and O_3_ were also formed in the microplasma-treated water [16]. After measuring the amounts of OH radicals, RNS (NO_3_^−^ and NO_2_^−^ only), dissolved O_3_, and H_2_O_2_ generated in the treated water, it was observed that the quantity of each reactive species or molecules remained unchanged after reaching its maximum. The time required to reach their peaks differed for different species. Fig 4 demonstrates the maximum concentrations of reactive species produced during plasma treatment: hydroxyl radicals reached (1.81 ± 0.15) × 10 ⁻ ⁵ M within 6 min; nitrogen oxides attained (500 ± 0.15) × 10 ⁻ ⁶ M within 3 min; dissolved ozone reached 1.5 ppm after 4 min; and H₂O₂ formed at a concentration of (2.5 ± 0.2) × 10 ⁻ ⁶ M within 8 min of treatment (mean ± SD, n = 3). The procedure for determining the concentration of individual reactive species has been described previously, and methodology is described in the supplementary section in 1S-3S text, [20,26,30]. To ensure that all the measured species were present at their maximum concentrations, we treated water for a minimum of 8 min with the plasma device. The pesticide-spiked produce samples were then washed as follows: with water bubbles, MPW, aerosolized MPW, and chlorine water for 4 min. We determined the role of each species on pesticide degradation using a passive method, a full description of the passive (radical scavenging) method is given in the supplementary information in S6 text and 2S Fig. To ensure that our microplasma treated water was free of O_3_, the solution was kept in a safety cabinet at room temperature and atmospheric pressure for two hours. Khan and Kim (2020) showed that the concentrations of oxides of nitrogen remain constant even after 7 days of treatment. A commercially available nitric oxide scavenger (S4 text) was used to create a nitric oxide-free environment [31].

### Effect of process parameters on pesticide reduction

**Fig 5:**
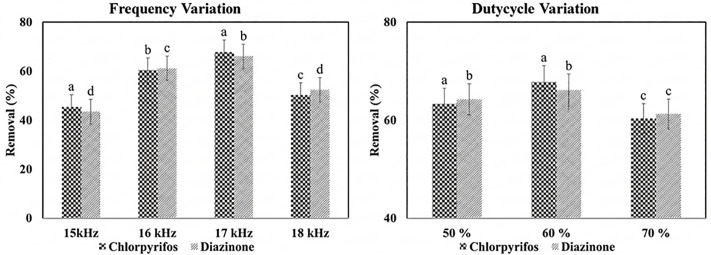
Removal percentage of chlorpyrifos and diazinon from broccoli surfaces at different working frequencies and duty cycles. Data are presented as mean ± standard deviation (n = 3). The temperature of the water solution was maintained at 22 ± 1 °C during the treatment. Statistical analysis was performed using factorial ANOVA to evaluate the effects of frequency, duty cycle, and pesticide type, with assumptions of normality and homogeneity of variance checked via Shapiro–Wilk and Levene’s tests. Significant differences (p < 0.05) were determined using Tukey’s honestly significant difference (HSD) test. Values marked with different lowercase letters indicate statistically significant differences (p < 0.05).

The optimum operational parameters of the device used in this study were checked by varying the operating frequency and duty cycle. After testing the degradation efficiencies of the pesticides at 15–18 kHz, the optimum degradation efficiency for both pesticides (Fig 3) in water was obtained at 17 kHz and 60% duty cycle during 4 min of treatment (Fig 2 and 3). Pesticide removal and/or degradation from the food surface was determined by selecting a broccoli sample and then checking the removal and/or degradation efficiencies of the pesticides in response to changes in operating frequency and duty cycle. We observed that 17 kHz and 60%, respectively provide higher degradation (Fig 5) efficiency. Hence, subsequent experiments were conducted using an operating frequency and duty cycle of 17 kHz and 60%, respectively, to achieve the highest possible degradation of pesticides from the contaminated produce surface.

### Measurement of pesticide degradation using HPLC

**Fig 6:**
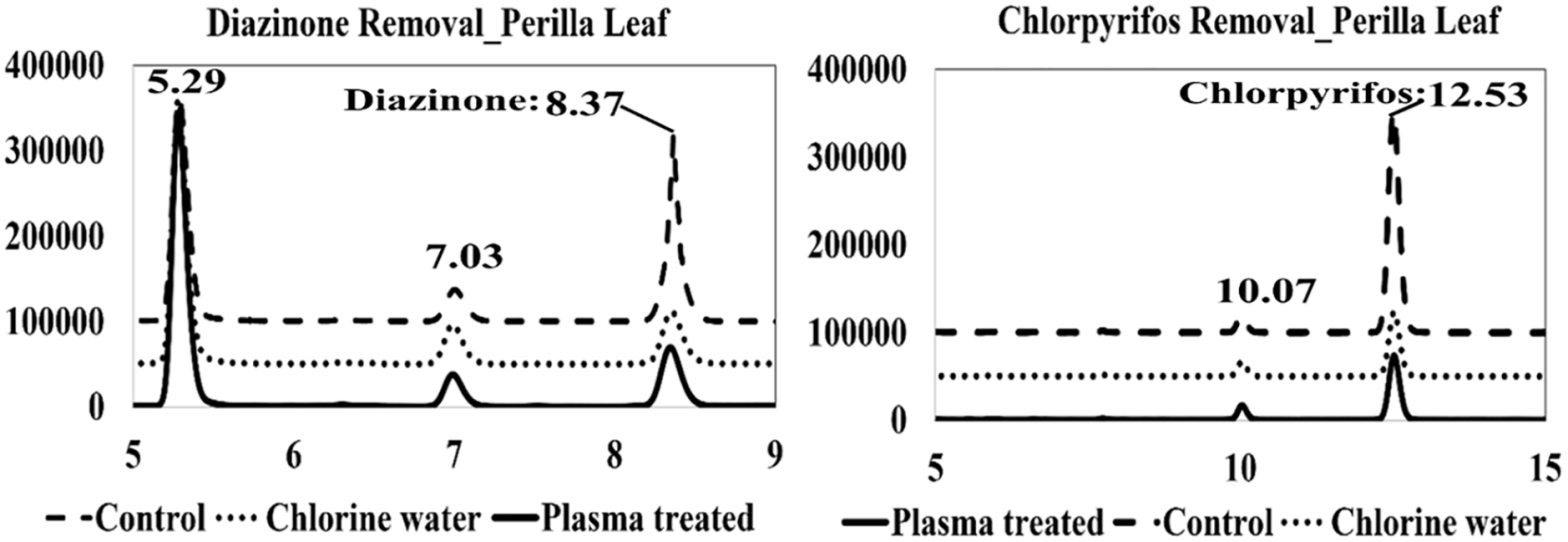
HPLC chromatogram of the control, and produce treated with microplasma and chlorine water. Treatments lasted for 4 min.

**Fig 7:**
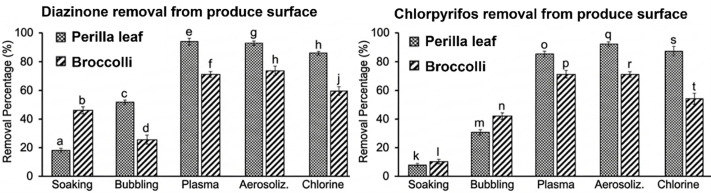
Removal efficiency (%) of diazinon and chlorpyrifos from perilla leaf and broccoli surfaces under various washing procedures. **The temperature of the washing solution was strictly maintained at 22 °C for 4 min.** Data are presented as mean ± standard deviation (n = 3). All data were analyzed using factorial ANOVA to evaluate the main and interaction effects of washing method, pesticide type, and temperature. Prior to analysis, assumptions of normality and homogeneity of variance were checked using Shapiro–Wilk and Levene’s tests, respectively. When significant differences were detected (p < 0.05), Tukey’s honestly significant difference (HSD) test was used for multiple comparisons. Values marked with different lowercase letters indicate statistically significant differences (p < 0.05).

The removal and/or degradation efficiencies of spiked pesticides from the food surface after different washing methods were evaluated by HPLC analysis. Pesticide degradation follows first-order reaction kinetics; detailed calculation methods are described in Supplementary information S5 text. To confirm steady degradation measurements, a strict protocol was followed in which produce samples were collected and prepared for HPLC analysis immediately after 4 min of microplasma treatment. Although residual microplasma produced species may continue to reduce pesticides over time, all samples were processed within the same timeframe. This standardized process reduces variability and ensures that the measured degradation primarily reflects the effect of the microplasma treatment. Fig 6 shows the chromatogram for the removal and/or degradation of diazinon and chlorpyrifos, Fig 7 displays the removal and/or degradation efficacies of diazinon and chlorpyrifos on different food surfaces following a 4 min wash. Independent of pesticide type, MPW treatment, aerosolized MPW, and chlorine water attained removal and/or degradation efficacies of 80.84–87.17% (± 2.3–3.1%) on perilla leaves and 51.74–67.77% (± 2.8–3.6%) on broccoli (mean ± SD, *n* = 3). In contrast, soaking and bubbling washing procedures resulted in relatively lower removal efficiencies, ranging from 7.88–29.83% (± 1.2–2.4%) for perilla leaves and 9.03–22.89% (± 1.5–2.6%) for broccoli (mean ± SD, *n* = 3). Washing with MPW, aerosolized MPW, and chlorine water achieved similar levels of pesticide reduction, which were significantly higher than those achieved by soaking and bubbling methods. All washing procedures resulted in significantly lower pesticide removal on broccoli compared to perilla leaves, indicating higher effectiveness on the latter. Particularly, diazinon was generally removed more efficiently than chlorpyrifos among all treatments.

### Effect of temperature on the pesticide reduction

**Fig 8:**
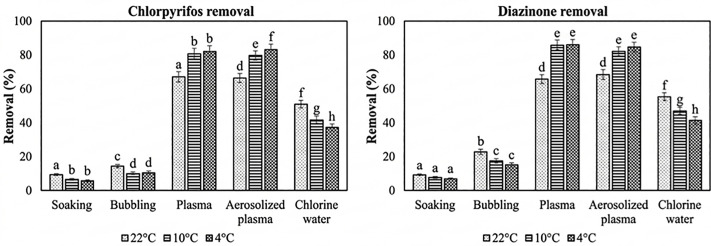
Effect of temperature on the removal efficiency (%) of chlorpyrifos and diazinon from broccoli using various washing procedures. **Data are presented for three temperature conditions (22°C, 10°C, and 4°C) across five treatments: Soaking, Bubbling, Plasma, Aerosolized plasma, and Chlorine water. Each bar represents the Mean ± SD (n = 3).** Different lowercase letters above the bars indicate significant differences (P < 0.05) between temperature treatments within each specific washing procedure according to Tukey’s HSD test. Values marked with different lowercase letters indicate statistically significant differences (p < 0.05).

**Fig 9:**
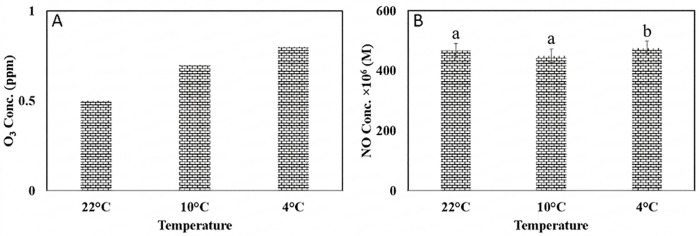
Effect of solution temperature on the concentrations of (A) gaseous ozone (O_3_) and (B) nitric oxide (NO) in water. For O_3_ **(A)**, error bars are omitted as fluctuations remained within the instrument’s precision limit. For NO **(B)**, data are presented as mean ± SD; different lowercase letters indicate statistically significant differences between temperature treatments (p < 0.05).

Fig 8 displays the effect of washing temperature on the degradation of diazinon and chlorpyrifos from broccoli surfaces. Temperature significantly influenced pesticide elimination during plasma-based washing, whereas a reverse trend was observed for chlorine-based treatment. For MPW, removal efficiencies were lowest at 22 °C (66.10–67.77% ± 2.5–3.2%), increased noticeably at 10 °C (81.37–86.32% ± 2.1–2.9%), and remained comparably high at 4 °C (83.25–86.32% ± 2.0–2.8%), with no considerable difference between 10 and 4 °C, regardless of pesticide type (mean ± SD, n = 3). In contrast, chlorine washing showed a clear temperature-related decrease in efficacy, attaining 51.74–57.00% (± 2.6–3.1%) at 22 °C, decreasing to 42.28–47.12% (± 2.3–2.9%) at 10 °C, and further decreasing to 38.25–41.54% (± 2.0–2.6%) at 4 °C (mean ± SD, n = 3). Overall, reducing the washing temperature from 22 to 10 °C improved pesticide removal and/or degradation during microplasma- and aerosolized MPW washing, whereas further reduction to 4 °C resulted in no additional enhancement. Conversely, the effectiveness of chlorine water increasingly decreased with lower temperature. As shown in Fig 9, ozone concentration increased with decreasing temperature, rising from 0.5 ppm at 22 °C to 0.75 ppm at 10 °C and 0.8 ppm at 4 °C. In contrast, nitrogen oxide concentrations remained essentially constant (5.00 × 10 ⁻ ⁶ M) across all temperatures, which may contribute to the enhanced pesticide degradation observed during plasma-based washing. Soaking and bubbling washing were not evaluated at reduced temperatures due to their limited pesticide removal efficiency under ambient conditions.

### Effect of the pesticide and microplasma on the color of produce

Table 1 shows the measurements of the color parameters (L*, a*, b*) for broccoli and perilla leaves before treatment, after treatment, and one-week post-treatment. The ΔE* values for perilla leaf washed with plasma and chlorine water are 1.38 ± 0.47 and 0.63 ± 0.35, respectively; and for broccoli, the values are 0.56 ± 0.87 and 1.65 ± 0.18, respectively. These changes were not statistically significant. Tiwari et al. (2008) proposed the following criteria based on the ΔE* value to characterize the overall changes in color: (1) If ΔE* > 3, then the color changes are considered very distinct; (2) if 1.5 < ΔE* < 3, then the color changes are considered as distinct; and (3) if ΔE* < 1.5, then color changes are considered as insignificant [32]. The calculated ΔE* values for the fresh produce were < 1.5, i.e., insignificant, except for broccoli with chlorine water treatment, where ΔE* = 1.65 ± 0.18 (distinct change). Chlorine water-treated broccoli turned yellowish after 7 days of storage, which may be due to the bleaching action of aqueous Cl_2_. Moreover, broccoli has a very rough surface, which tends to slow down the drying process, thus resulting in its bleaching.

**Table 1:** Comparative analysis of color parameters (L*, a*, b*) and total color difference (∆ E^*^) for perilla leaves and broccoli across different disinfection treatments.

Treatment	Perilla Leaf				Broccoli			
	Color Parameters				Color Parameters			
	L	a	b	ΔE* ± SD	L	a	b	ΔE* ± SD
Non-treated	30.76	−7.92	8.38	0.94 ± 0.96	37.40	−7.06	8.32	0.28 ± 0.64
Plasma treated	31.78	−7.84	9.32	1.38 ± 0.47	38.27	−7.54	9.25	0.56 ± 0.87
Chlorine water treatment	31.99	−8.90	10.62	0.63 ± 0.35	36.91	−7.81	10.16	1.65 ± 0.18

* Values are presented as mean ± standard deviation (n = 3)

## Discussions

Pesticide-spiked produce washing with MPW showed promising results, supporting its practical application. Although the degradation mechanism was previously recognized under controlled conditions, comparable trends were observed in produce samples, supporting the effectiveness of the treatment in reducing surface residues. Quantitative comparison proved that MPW related and chlorine-based washing methods distinctly outperformed conventional soaking and bubbling washing. For perilla leaves, MPW, aerosolized MPW, and chlorine water yielded an average removal and/or degradation effectiveness of about 84.0%, compared with 18.9% for soaking and bubbling washing, representing an increase of ~65 percentage points (≈4.5-fold). Likewise, for broccoli, MPW related and chlorine treatments achieved an average removal and/or degradation efficiency of ~59.8%, which was ~ 44 percentage points higher (≈3.7-fold) than that obtained by conventional washing methods. These results clearly demonstrate the greater efficiency of MPW based and chlorine washing in pesticide removal and/or degradation across different food surfaces. Specifically, the chemical species mainly responsible for pesticide degradation are the oxides of nitrogen, followed by dissolved O_3_ and/or OH. The trace amount of H_2_O_2_ (2.5 × 10^−6^ M throughout the experiment) produced in our study did not significantly contribute to the degradation process [26]. DBD plasma and microplasma devices with air feed gases produce somewhat similar species in water, at levels that do not differ significantly. Therefore, the radicals generated by both plasma devices play similar roles in pesticide degradation. The experiment was conducted at a constant water temperature (22 ± 1°C), and care was taken to ensure that temperature did not influence pesticide reduction. After 4 min of plasma treatment, varying degrees of pesticide reduction were observed across different produce surfaces (Fig 7). In addition, the extent of pesticide reduction differed significantly among the various washing methods applied. Factors such as food surface roughness, thickness, and wetness, as well as the types of pesticide and microplasma discharge device determine the potential for pesticide removal from produce surfaces [33,34]. Pesticide solutions were applied to both the front and back surfaces of the samples to account for the effect of surface roughness on removal and/or degradation behavior. Furthermore, all samples were air-dried prior to treatment to minimize variability arising from differences in moisture content, which is known to affect pesticide removal and/or degradation. Furthermore, higher moisture content tends to speed up the washing procedure from the surface to the water jar, which may result in more false positive results in our experiments. A lower proportion of pesticide (approx. 40.5%) was removed from broccoli due to its rougher surface compared to perilla leaf. During the washing procedure, transfer of pesticide from the contaminated produce surface into the water results in cross contamination; therefore, it is important to identify the presence of pesticides in the leftover water. Previously, we demonstrated that approximately 99% of diazinon and chlorpyrifos in standard solutions were degraded within 3 min of microplasma treatment. Based on these findings, the presence of residual pesticides in the washing water after 3 min of microplasma treatment was not further examined [26]. Although pesticide residues in the wash water were not directly quantified in this study, the applied plasma treatment duration exceeds the time required for near complete degradation in standard solutions, as shown in our previous work. However, the presence of pesticide residues in the wash water cannot be excluded. Future studies will include direct quantification of rinse water residues to further validate this assumption. For the prevention of any cross contamination, this approach may be superior to any other available methods. While the pesticide concentrations used in this study surpass those commonly encountered in real-world situations, this conventional approach does not reflect consumer exposure but rather establishes a stringent test of treatment efficiency. Given the lower residue burdens typically present on fresh produce, comparable or enhanced removal efficiency is expected under practical conditions, supporting the potential applicability of the proposed washing approach.

Non-thermal plasma is an emerging technology in the produce industry [34,35]. Several non-thermal plasma methods are currently being evaluated with respect to their ability to remove pesticide residues from produce surfaces. The efficiency of pesticide removal and/or degradation varies depending on the produce surface, which depends on numerous factors such as the type of plasma sources, operational and ambient conditions, the type of produce item, etc. Cold plasma treatment reduces 100 ppm paraoxon from apples by 95.5% [36]. Dorraki et al. (2016) showed that 15 min of treatment can reduce 25 ppm of diazinon on the surface of cucumber by 88% [37]. Some studies have shown that the chlorpyrifos were reduced from the surface of mango and tomato by 74% and 89% within 5–10 and 6 min, respectively [38]. However, the degradation efficiency of pesticides also depends on the type of plasma source used. For example, Ranjitha Gracy, Gupta, and Mahendran (2019a) showed that 54% chlorpyrifos was reduced within 15 min when they applied different types of plasma sources than the previous approach on same vegetables, same pesticide and same treatment time indicating [38]. Seyyedeh Mahbubeh Mousavi et al. (2021) investigated the effects of voltage (10 kV and 13 kV) of application of DBD plasma on the degradation of chlorpyrifos and diazinon residues from apple surfaces that had been applied at different concentrations (500 ppm and 1000 ppm) [39]. On the one hand, maximum detoxification (87.38%) of 1000 ppm diazinon occurred at 13 kV after 10 min of plasma exposure. On the other hand, the same exposure time and voltage detoxified chlorpyrifos by only 58.33%. Moreover, the degradation efficiency of apple differed from that on cucumber. These findings highlight the effectiveness of various types of plasma in reducing pesticide residues on produce surfaces; moreover, voltage, exposure time, and the type of produce are important features that can impact the extent of pesticide degradation. It should be noted that straight comparison among heterogeneous plasma systems is inherently limited due to differences in reactor configuration, plasma characteristics, and experimental conditions. The microplasma device used in this study varies from conventional non-thermal plasma systems by enabling efficient production of reactive oxygen species under relatively low-power working conditions. In addition, the device is lightweight and cost-effective, offering clear practical advantages and strong potential for scalable commercial applications.

The degradation of diazinon and chlorpyrifos by plasma in water follows first-order degradation kinetics (S2 Fig), detail equation shown in S5 Text. The mechanism of degradation involves the oxidation of pesticide molecules and the generation of one or more smaller fragmented products. S2 Fig shows different microplasma generated species play diverse roles in pesticide reduction. Among the various species produced by microplasma, the most important in terms of diazinon and chlorpyrifos degradation are oxides of nitrogen, followed by dissolved O_3_ and/or OH radicals. Detailed methodologies and analyses used to elucidate the roles of individual reactive species are provided in S6 Text and S2 Fig. Some of the interactions involving O_3_ occur due to the slightly acidic nature of the treated water. In our study, the amount of H_2_O_2_ generated was very low; therefore, it played no significant role in degrading the pesticides. However, it should be noted that pesticide degradation depends on the types of electrodes and feed gases used. Additionally, dropping the washing temperatures meaningfully improved pesticide reduction during MPW treatments, which can be accredited to the increased concentration of dissolved ozone at reduced temperatures (Fig 9). Precisely, lowering the temperature from 22 °C to 10 °C increased ozone concentration from 0.5 to 0.75 ppm (≈50% increase), accompanied by an enhancement in pesticide removal and/or degradation from approximately 66–68% to 81–86% (≈20–25 percentage-point increase). A further lessening to 4 °C resulted in only a marginal increase in ozone concentration (0.8 ppm) and no considerable additional enhancement in removal and/or degradation efficacy, signifying a saturation effect. In contrast, chlorine washing exhibited a clear decline in efficiency with reducing temperature, with pesticide reduction decreasing from about 52–57% at 22 °C to 38–42% at 4 °C (≈15–18 percentage-point reduction). This opposite trend is likely attributable to reduced molecular kinetic energy at lower temperatures, which reduces the frequency and effectiveness of reactive collisions involved in chlorine-mediated oxidation. Overall, these results validate that temperature plays a critical role in governing the efficiency of ozone-based versus chlorine-based pesticide removal mechanisms. Notably, MPW, aerosolized MPW, and chlorine water exhibited similar overall pesticide removal and/or detoxification efficiencies at room temperature, representing that the choice of a suitable washing system should be guided by application-specific requirements rather than efficiency alone.

**Fig 10:**
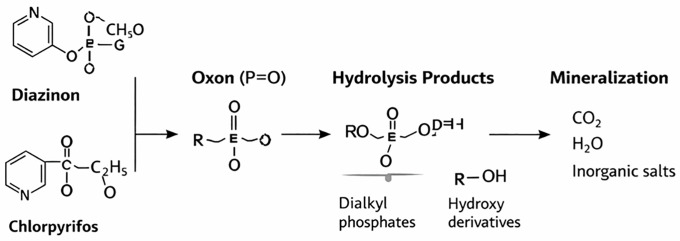
Proposed degradation pathways of diazinon and chlorpyrifos mediated by hydroxyl radicals and/or ozone, involving oxidation to the corresponding oxon derivatives, hydrolysis to dialkyl phosphates and hydroxylated intermediates, and subsequent mineralization to CO₂, H₂O, and inorganic salts [40–42].

**Fig 11:**
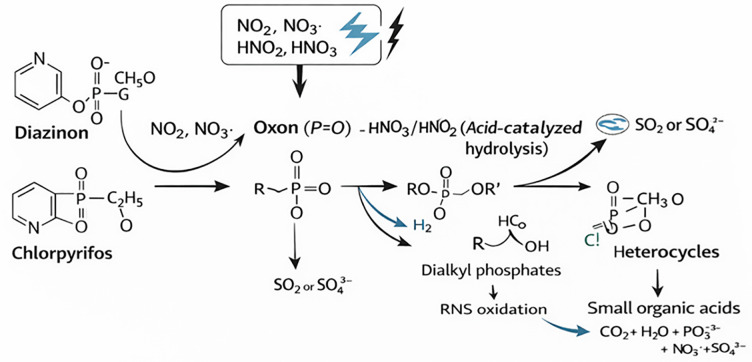
Proposed degradation pathways of diazinon and chlorpyrifos by oxides of nitrogen produced by microplasma [42–44].

Nitrogen oxides and reactive nitrogen species produced by microplasma drive the degradation of organophosphate pesticides through sequential oxidation, hydrolysis, and mineralization. Electrophilic NO₂· and NO₃· oxidize thionophosphate (P = S) bonds to oxon (P = O) intermediates, which undergo acid-catalyzed hydrolysis to form dialkyl phosphates and polar metabolites. Successive RNS-mediated oxidation promotes ring cleavage and fragmentation, yielding low-molecular-weight species that are completely mineralized to CO₂, H₂O, phosphate, nitrate, and sulfate, highlighting the main role of nitrogen-oxide chemistry in plasma-driven degradation [40–42]. Reaction outline figure with curved arrows for the degradation of diazinon and chlorpyrifos is shown in Fig 10. The degradation route of diazinon and chlorpyrifos by O_3_ likely involves oxidation and various chain reactions to formed peroxide and hydroxyl radicals. Ozone (O₃) can oxidize the P = S bond of organophosphorus pesticides, creating the corresponding oxon derivatives (P = O). In aqueous or MPW systems, O₃ also decomposes to produce hydroxyl radicals (•OH) and peroxides, which further attack alkyl or aromatic side chains, leading to cleavage of P–O–C bonds and construction of smaller, more polar degradation products [42–44]. Reaction scheme figure with curved arrows for the degradation of diazinon and chlorpyrifos is shown in Fig 11.

The interaction of OH radicals with flavonoids and ascorbic acid, which act as natural OH radical scavengers, raises concerns about the potential degradation of the nutritional and chemical properties of microplasma-treated water. The oxidative breakdown of flavonoids, particularly upon exposure to hydroxyl free radicals and following pathways similar to those observed in heat-induced oxidative cleavage, leads to the creation of lesser-molecular-weight phenolic compounds. Previously, we reported minimal reductions in the quercetin, kaempferol, and ascorbic acid contents of onion and various types of lettuce following plasma treatment [31,45]. Therefore, the potential change of produce chemical properties resulting from plasma treatment was not examined further in this study. We attribute the lack of significant chemical changes in response to plasma treatment to the limited penetration depth of OH radicals and their very short lifetime. Overall, the plasma treatment performed well at contaminant degradation without significantly altering the quality of the treated produce. We previously showed that the plasma process generated different fragments of pesticides. The presence of different microplasma generated radicals suggest that different reduction mechanisms occur. In our previously published studies, mass spectrometric analysis identified the primary and secondary fragments formed during the degradation of diazinon and chlorpyrifos, and their signal intensities were shown to decrease progressively over time [26,46]. These results suggest that plasma-induced reactions continue after pesticide degradation, and these reactions produce simpler, less toxic components. This detoxification is similar to the advanced oxidation processes used in water treatment. Post-plasma treatment leads to the formation of degradation fragments that are suggested to be less toxic than the parent pesticides, as depicted in the proposed pesticide fragmentation mechanisms shown in Figs 9 and 10. Some researcher reported similar findings for various pesticides, including chlorpyrifos, diazinon, and nitenpyram [26,46]. Additionally, further studies are needed to identify potential toxic byproducts that do not undergo further reactions. In particular for our future studies, LC-MS/MS, IR and NMR analyses would be used to check if fragments generated by the plasma process.

Analysis of the changes in color parameters (L*, a*, b*) of broccoli before and after microplasma-treated water treatment showed very slight changes that could not be differentiated by the naked eye. The differences that were detected by the camera were not very significant (p > 0.05). The hardness and hydrophobic nature of perilla leaves do not allow water to leach through; hence, no significant color changes were observed. The midrange color changes observed for broccoli one week after the treatment were likely due to the oxidizing nature of chlorine water. Overall, color changes for all the samples remain at a satisfactory level. Our findings can inform future optimization efforts of washing systems in various industries, enabling the identification of the most suitable approach for particular situations. In particular, the data obtained from the aerosolized MPW may prove highly valuable for its application in the food processing industry. This system offers a promising solution for maintaining hygiene and safety in environments where it is not feasible to wash every component of machinery, conveyor belts, and other equipment on a daily basis.

## Limitations

Although this study delivers valuable outcomes for pesticide removal and/or degradation using microplasma discharged water, certain limitations should be acknowledged. First, the experiments were conducted using controlled spiking levels in accordance with validation guidelines, which may not fully capture the distribution, binding behavior, and heterogeneity of naturally incurred pesticide residues under field conditions. Second, the existence of pesticide residues or their fragmented derivatives in treated water was not directly determined, limiting decisions concerning comprehensive decontamination. Third, comprehensive identification of degradation intermediates using LC-MS/MS, IR and NMR was not achieved, limiting complete mechanistic understanding. It should be noted that the present study does not distinguish between physical removal and chemical degradation of pesticide residues, as residues in the washing solution were not quantified. Lastly, the scalability of the treatment procedure and its effectiveness in actual samples continue to be validated. Therefore, toxicity and safety inferences cannot be conclusively established without additional study. Addressing these drawbacks in forthcoming studies will increase the applicability and strength of the findings.

## Conclusions

In the present study, the MPW system performed well in reducing pesticide residues (through removal and/or degradation) on the surface of pesticide-spiked fresh produce. Pesticide degradation depended on the formation of different reactive particles and the temperature of microplasma treatment. Surface roughness also played a significant role in pesticide removal and/or degradation. Microplasma water treated produce items did not undergo significant changes in color. Produce washing using an aerosolized approach also demonstrates potential for pesticide removal and/or degradation, thereby increasing its applicability in the food industry, mostly in situations where such a method is both appropriate and essential. However, further studies are required to optimize pesticide removal and/or degradation before the MPW device can be reliably applied in the produce processing and washing industry.

## Supporting information

S1 Fig Optical emission spectra (OES) of clean dry air gas plasma produced from a microplasma produced system. (TIF)

S2 Fig Microplasma degradation kinetics of diazinon and chlorpyrifos in the presence of different microplasma-generated reactive species, solution was selectively controlled using various scavenging method. (TIF)

S1 Table Recovery of pesticides from spiked blank matrices. (DOCX)

S1-S6 Text (DOCX)
