# Supplementary figures and images for: Removal efficiency of pesticide residues on pesticide-spiked Perilla Leaf and Broccoli surfaces using microplasma-treated water
Source: PLoS One. 2026 Jun 26;21(6):e0351955. doi: 10.1371/journal.pone.0351955 (PMC13309029; doi:10.1371/journal.pone.0351955)

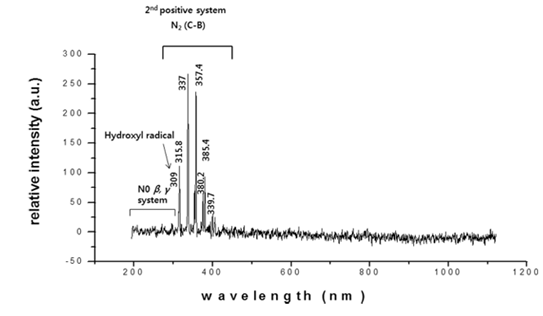

Supplement: S1 Fig — (TIF) [file pone.0351955.s001.tif]

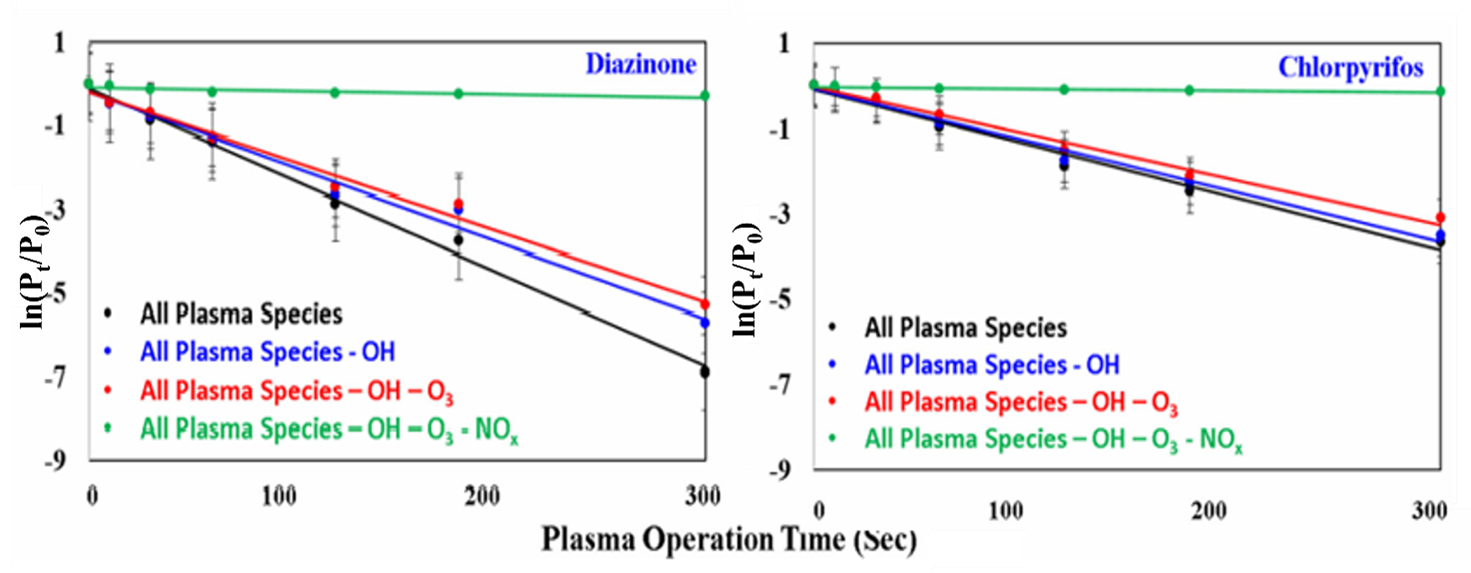

Supplement: S2 Fig — (TIF) [file pone.0351955.s002.tif]
