# Supplementary material for: Removal efficiency of pesticide residues on pesticide-spiked Perilla Leaf and Broccoli surfaces using microplasma-treated water
Source: PLoS One. 2026 Jun 26;21(6):e0351955. doi: 10.1371/journal.pone.0351955 (PMC13309029; doi:10.1371/journal.pone.0351955)
Supplement: S1 Table — (DOCX) [file pone.0351955.s003.docx]

**S1 Table.** **Recovery of pesticides from spiked blank matrices**

| **Pesticide** | **Spiked Concentration (µg/g)** | **Mean Recovery (%)** | **Standard Deviation (SD, %)** | **RSD (%)** |
| --- | --- | --- | --- | --- |
| Chlorpyrifos | 0.1 | 95.2 | 3.1 | 3.26 |
| Chlorpyrifos | 0.5 | 97.8 | 2.5 | 2.55 |
| Diazinon | 0.1 | 93.6 | 2.9 | 3.10 |
| Diazinon | 0.5 | 96.4 | 3.2 | 3.32 |
