# Supplementary material for: Removal efficiency of pesticide residues on pesticide-spiked Perilla Leaf and Broccoli surfaces using microplasma-treated water
Source: PLoS One. 2026 Jun 26;21(6):e0351955. doi: 10.1371/journal.pone.0351955 (PMC13309029; doi:10.1371/journal.pone.0351955)
Supplement: S1-S6 Text — (DOCX) [file pone.0351955.s004.docx]

**Supplementary Information**

**For**

**Removal Efficiency of Pesticide Residues on** **Pesticide-Spiked Perilla Leaf and Broccoli Surfaces Using Microplasma-Treated Water**

**Muhammad Saiful Islam Khan^1^, Yun-Ji Kim^2,3†^ and Jae-Hwan Ahn^2^**

^1^Department of Biomedicine and Life Science, School of Medicine, Central Asian University, 264, Milliy bog St, Tashkent, 111221, Uzbekistan.

^2^Korea Food Research Institute, 245, Nongsaengmyeong-ro, Iseo-myeon, Wanju-gun, Jeollabuk-do, 55365, Republic of Korea

^3^Department of Food Biotechnology, Korea University of Science and Technology, 217, Gajeong-ro, Yuseong-gu, Daejeon, 34113, Republic of Korea

†Corresponding Author: yunji@kfri.re.kr

**S1 Text. Measurement of OH radical concentration**

Chemical dosimetry was performed using terephthalic acid (TA; Sigma-Aldrich, USA) as a selective scavenger for hydroxyl (•OH) radicals. TA reacts specifically with •OH radicals and does not interact with other reactive species such as O₂•⁻, HO₂•, or H₂O₂. TA was dissolved in 0.5 M NaOH to prepare a 2.0 × 10⁻² M solution, as TA is poorly soluble under neutral or acidic conditions. The pH of the resulting TA solution was 9.73. Preliminary experiments were conducted to measure the optimal TA concentration for •OH quantification. Upon reaction with •OH radicals, TA is converted to 2-hydroxyterephthalic acid (HTA; Sigma-Aldrich, USA), which exhibits strong fluorescence emission at 425 nm when excited at 310 nm, whereas TA itself is non-fluorescent. The fluorescence concentration of HTA is stable over a pH range of 6–11. HTA fluorescence was determined by a multi-mode microplate reader (SpectraMax i3, Molecular Devices). Quantitative measurement of HTA was achieved by an additional calibration curve to ensure measurement accuracy and reproducibility, and the concentration of •OH radicals was measured on the basis of the known reaction stoichiometry between TA and •OH radicals. The formation rate of •OH radicals was measured accordingly. Comprehensive experimental trials for •OH radical quantification is described elsewhere [1]. All quantitative data were obtained using calibrated regression models with acceptable goodness-of-fit (R²).

**S2 Text. Measurements of nitric oxide concentrations**

A commercially available nitric oxide assay kit (QuantiChrom; BioAssay Systems, Hayward, CA, USA) was used to measure nitrogen oxides in the MPW system, mainly nitrate (NO₃⁻) and nitrite (NO₂⁻) those are the stable final products of nitric oxide reactions in aqueous media. Subsequent microplasma exposure, samples were gained at programmed time intervals and analyzed based on the manufacturer’s instructions. Briefly, the assay is based on the conversion of nitrate to nitrite, followed by colorimetric identification, allowing the determination of total nitrogen oxide concentration. All quantitative data were obtained using calibrated regression models with acceptable goodness-of-fit (R²), and calibration curves were established to ensure measurement accuracy and reproducibility. All measurements were made in triplicate to confirm reproducibility.

**S3 Text. Measurements of H_2_O_2_ concentrations:**

Due to the extremely short lifetime of hydroxyl (•OH) radicals, their recombination in aqueous media leads to the formation of hydrogen peroxide (H₂O₂). Therefore, a commercially available H₂O₂ assay kit (Amplex®, Molecular Probes, Eugene, OR, USA) was employed to quantify H₂O₂ generated in the plasma-treated system. Sample analysis was conducted according to the manufacturer’s protocol, based on the horseradish peroxidase–mediated oxidation of Amplex Red to the fluorescent product resorufin, allowing sensitive and selective detection of H₂O₂. All quantitative data were obtained using calibrated regression models with acceptable goodness-of-fit (R²), and calibration curves were established to ensure measurement accuracy and reproducibility.

**S4 Text. NO scavenger.**

The dissolved nitric oxide in water produced by microplasma generated system was scavenged by 2-(4-Carboxyphenyl)-4,4,5,5-tetramethylimidazoline-1-oxyl-3-oxide potassium salt (c-PTIO, Sigma-Aldrich, MO, USA). The stoichiometric quantity of c-PTIO was used in this study, c-PTIO and NO reacts in 1:1[2]. To obtain NOx free environment 150 µM of cPTIO was used.

**S5 Text:** **Kinetic modeling of pesticide degradation**

Pesticide degradation efficiency (η) during microplasma treatment was calculated using Eq. (1):

η (%) = (P_0_-P_t_)/P_0_ × 100………(1)

where P_0_​ is the preliminary pesticide concentration and P_t_​ is the pesticide concentration at treatment time t.

The degradation of pesticides under microplasma treatment followed a pseudo–first-order kinetic model. Kinetic parameters were obtained by fitting the experimental concentration–time data using a nonlinear least-squares regression approach based on the Levenberg–Marquardt algorithm implemented in MATLAB (version 7.5; The MathWorks, MA, USA).

The pseudo–first-order kinetic model is expressed as Eq. (2):

$ln=\frac{\mathrm{Pt}}{P0}$……………….(2), where k is the apparent first-order rate constant (s⁻¹) and t is the microplasma treatment time (s). The goodness of fit of the kinetic model was evaluated using the coefficient of determination (R²). The rate constant k was determined from the slope of the linear regression of ln (P_t_/P_0_) versus treatment time using pesticide concentration data obtained at different microplasma processing intervals.

**S6 Text. Role of various reactive species evaluated using a passive technique**

To determine the role of individual reactive species to pesticide degradation, MPW was used under four separate conditions using a passive scavenging approach. In **Mode 1**, all microplasma-produced reactive particles were existing. In **Mode 2**, hydroxyl radicals were selectively scavenged by the addition of ethanol. In **Mode 3**, both ozone and hydroxyl radicals were removed; an ozone-free environment was achieved by letting the MPW to stand under ambient conditions for 2 h to permit natural ozone decay. In **Mode 4**, ozone, hydroxyl radicals, and nitrogen oxides were simultaneously removed, with nitrogen oxides removed using a specific NOₓ scavenger.

These experimental modes signify passive strategies for measuring the relative effect of individual reactive particles in pesticide degradation. Since multiple reactive species coexist in the MPW system, separating their individual contributions is vital for understanding the fundamental degradation mechanisms. By selectively suppressing specific species, the separate effects of each reactive constituent on pesticide reduction can be assessed.

The degradation kinetics under these four modes are presented in **S2 Fig**. The results specify that nitrogen oxides contributed most meaningfully to the degradation of both pesticides, followed by hydroxyl radicals and ozone. These discoveries highlight the leading role of nitrogen-based reactive species in MPW pesticide degradation under the studied conditions.

**S7 Text. Recovery experiments were done using spiked blank matrices to assess the accuracy and reliability of the analytical method.**

Known concentrations of each pesticide were added to blank matrices, and the samples were processed following the same procedure as for real samples. The mean recovery (%) was calculated from three independent replicates, along with the standard deviation (SD) and relative standard deviation (RSD, %). The results (S1 Table) specify that the technique offers acceptable recovery (generally between 90–100%) for all tested pesticides, representing that the method is accurate and reproducible for the target analytes.
