# Supplementary material for: Removal efficiency of pesticide residues on pesticide-spiked Perilla Leaf and Broccoli surfaces using microplasma-treated water
Source: PLoS One. 2026 Jun 26;21(6):e0351955. doi: 10.1371/journal.pone.0351955 (PMC13309029; doi:10.1371/journal.pone.0351955)
Supplement: Attachment — Submitted filename: Response to Reviewer.docx [file pone.0351955.s005.docx]

We sincerely **thank** the reviewer for their careful evaluation of our manuscript and for the constructive comments and suggestions. These insights were extremely helpful and have significantly improved the clarity, quality, and scientific rigor of the manuscript. All comments have been carefully considered and addressed in detail below.

5. Review Comments to the Author
Please use the space provided to explain your answers to the questions above. You may also include additional comments for the author, including concerns about dual publication, research ethics, or publication ethics. (Please upload your review as an attachment if it exceeds 20,000 characters)

Reviewer #1: This study explores the application of microplasma-treated water for removing organophosphorus pesticides (diazinon and chlorpyrifos) from perilla leaves and broccoli surfaces, demonstrating technical novelty compared to chlorinated water treatment. The topic addresses practical needs in agricultural product safety and provides valuable insights for pesticide residue removal technologies. While the research is well-motivated, several aspects require clarification and improvement to strengthen the manuscript.

1. Section 2.1 should provide a detailed description of the sample treatment process. For instance, Line 79 mentions treating the produce surface with 2000 ppm pesticide solution, but the method of application (e.g., immersion, droplet coating) and the sample mass used are unclear. Please clarify these experimental details.

**Response:** We have clarified the detailed description of the sample treatment process in section **“****Pesticide contamination in produce samples”.** Can be found in line 94 - 103.

2. Section 2.5 only describes the analytical method for diazinon. As chlorpyrifos is also investigated, its testing conditions must be provided. Additionally, the method for calculating degradation efficiency should be explicitly stated.

**Reply:** Chlorpyrifos detail is included in line 199 - 206. The method for calculating degradation efficiency also shown in the revised draft (supplementary information **Text 6**).

3. The pretreatment process involves 15-minute ultrasonication. Did the authors consider whether residual active substances on the surface might continue degrading pesticides during ultrasonication? Parameters such as ultrasonic power and temperature should be specified.

**Response:** We thank the reviewer for the comment. However, there appears to be a misunderstanding regarding the experimental procedure. The 15-minute ultrasonication step was **not used as a pretreatment**, but was applied **after the treatment** solely to facilitate the detachment and homogenization of surface residues for analytical extraction. Therefore, no residual active species were present during ultrasonication that could contribute to additional pesticide degradation. We have now added the missing operational parameters. The ultrasonication was performed using a bath sonicator at **40 kHz frequency**, **150 W power**, and a controlled **water temperature of 25 ± 1°C**. These details have been included in the *Materials and Methods* section to improve procedural transparency (Lines 171–174).

4. The use of 2000 ppm pesticide concentration significantly exceeds maximum residue limits (MRLs) and realistic field residue levels (typically ppm to ppb range). Validation data near actual residue levels should be included. Moreover, the actual residual concentration after sample treatment is not provided.

**Response:** We agree that 2000 ppm exceeds typical residue levels found on fresh produce. This concentration was intentionally selected for two specific reasons, which we have now clarified in the revised manuscript:

1. **Methodological Standardization:** High spiking levels are commonly used in degradation studies to ensure accurate quantification, minimize analytical variability at early time points, and enable clear monitoring of degradation kinetics across treatments.
2. **Stress-Testing the System:** The elevated concentration allows evaluation of the treatment’s capacity under worst-case or extreme contamination scenarios, which provides insight into the robustness and upper performance limits of the technology.

To address the reviewer’s concern about practical relevance, we have added a statement in the Discussion acknowledging that typical field residue levels are much lower and that real-world performance is expected to be similar or higher due to the lower pesticide burden. We also clarified that the purpose of the 2000 ppm condition is not to model actual consumer exposure but to provide a controlled, conservative test of degradation efficiency. These explanations have been incorporated into the revised Introduction (justification of experimental design) and Discussion (practical applicability). Introduction part: Line 85 - 88; Discussion Part: Line 395 – 400.

5. Fig. 2A lacks error bars. Although the microplasma treatment time was 8 minutes, Figs. 2B and 2C do not show data for the full duration. Please provide complete time-course data.

**Response:** The ozone concentration remained essentially constant during the 8-minute treatment, with minimal fluctuation. Given the high precision of the measurement device, error bars were not included, as they would not meaningfully reflect variation. For transparency, we have clarified this point in the figure legend.

**Additional Note on Figs. 2B and 2C:** Data at longer treatment times are not shown because no significant changes occurred after the reactive species reached their maximum concentration. This approach is now clarified in the figure legend.

6. Fig. 6 (HPLC chromatograms) should clearly label the peak positions corresponding to the detected pesticides.

**Response:** The HPLC chromatograms in Fig 6 were included primarily to illustrate overall pesticide degradation patterns rather than for quantitative peak identification. Detailed peak assignments and quantitative analysis have already been reported in the Methods section and validated through calibration and recovery experiments. However, in the revised manuscript peak position were labeled clearly in the figure.

7. In Section 2.5, the authors claim that all contaminated samples were treated for 4 minutes using different methods, but the specific procedures for these four treatments are not described. Please elaborate.

**Response:** Among the four methods tested, only **plasma treatment** resulted in a significant reduction of pesticides; the other methods showed negligible effects. For this reason, detailed procedures for the non-effective methods were not originally described. Nevertheless, in response to this comment, we have **briefly summarized the treatment conditions for all four methods** in the revised manuscript (**Different modes of produce washing section**) to improve clarity (lines 154–166).

8. References should be formatted uniformly. For example, Reference 11 requires verification, and all references with DOI numbers should include their corresponding DOI links.

**Reply:** DOI links have been included for all references where available to ensure completeness and traceability.

Reviewer #2: Dear Authors,
The topic of your manuscript — the use of microplasma-treated water (MPW) for the removal of pesticide residues from fresh produce — is relevant and aligns with current interests in sustainable and chemical-free decontamination methods. The concept has potential scientific and practical significance, and the experimental approach demonstrates effort in exploring alternative postharvest treatments.
However, after a detailed evaluation, it appears that the manuscript requires substantial revision to meet scientific and methodological standards expected for publication in PLOS ONE. The study design lacks sufficient analytical validation, several methodological descriptions are incomplete or imprecise, and the data interpretation often extends beyond the evidence presented.
Specific and detailed comments are provided below to help improve the rigor, transparency, and reproducibility of your work. Addressing these issues thoroughly will be essential before the study can be reconsidered for publication.
Kind regards,

Reviewer
Introduction
General comments:
The introduction presents a potentially relevant topic but does not convincingly justify the research gap or demonstrate the novelty of applying microplasma-treated water (MPW) for pesticide removal. A concise, technically precise revision should focus on: (i) the real and documented issue of pesticide residues on fresh produce, (ii) the limitations of current decontamination techniques, and (iii) the specific advantages and innovative aspects of MPW.

**Response:** We appreciate the reviewer’s emphasis on strengthening the scientific rationale and novelty in the introduction. In response, we have substantially revised this section to address the three key areas highlighted:

**(i) Documented issue of pesticide residues:**
We now provide a concise summary of the prevalence of pesticide residues on fresh produce, supported by recent global monitoring reports (e.g., EFSA, FDA, FAO) and peer-reviewed studies demonstrating potential health risks and regulatory concern.

Inclusion: Can be found in line 37 to 43.

New references:

8. European Food Safety Authority (EFSA). 2023. The 2021 European Union report on pesticide residues in food. *EFSA Journal*. 21:e08033. https://doi.org/10.2903/j.efsa.2023.8033
9. U.S. Food and Drug Administration (FDA). 2023. *Pesticide Residue Monitoring Program Fiscal Year 2021 Annual Summary*. Silver Spring (MD): U.S. Food and Drug Administration.

10. Kim KH, Kabir E, Jahan SA. 2017. Exposure to pesticides and the associated human health effects. *Sci Total Environ*. 575:525–535. https://doi.org/10.1016/j.scitotenv.2016.09.009

**(ii) Limitations of current decontamination techniques:**

The revised introduction now discusses the shortcomings of conventional washing methods (e.g., tap water, chlorinated water, ozone, organic acids), including limited removal efficiency, chemical residues, cost, or safety concerns. These limitations clearly support the need for alternative, safe, and effective decontamination strategies. Line 54 - 62 revised substantially.

**(iii) Advantages and novelty of MPW:**

We have clarified the innovative aspects of microplasma-treated water, including its capability to generate reactive oxygen and nitrogen species (RONS), its antimicrobial and oxidative potential, and its emerging application in food decontamination. These additions significantly improve the scientific justification for the study and clarify the novelty of applying MPW for pesticide degradation. We thank the reviewer for pointing out this gap, which has helped strengthen the clarity and impact of the manuscript. Detail can be found in line 74 – 85.

The description of organophosphorus pesticides (OPPs) as “highly water-soluble” and “substituting organochlorines” is oversimplified and partially inaccurate. OPPs are moderately polar compounds, and their substitution for organochlorines was primarily driven by lower environmental persistence rather than higher solubility. This section should be revised using accurate chemical reasoning and supported by appropriate scientific references.

**Response:** We agree that our original wording was oversimplified. The replacement of organochlorine pesticides (OCPs) by organophosphorus pesticides (OPPs) was primarily due to their **much lower environmental persistence and lower bioaccumulation potential**, rather than higher water solubility. We have revised the manuscript to reflect that OPPs are generally **moderately polar compounds with low-to-moderate water solubility**, depending on the specific molecule. Original manuscript is revised can be found in line 46 - 48.

Line 31 – Why are U.S. statistics cited when the research was conducted in Korea? Data relevant to the study region should be presented.

**Response:** We agree that regional data are more appropriate for supporting the context of this study. Accordingly, we have removed the U.S. statistics and replaced them with **Korea-specific data** reflecting fresh produce consumption trends. These Korea-specific data have now been incorporated into the revised introduction, ensuring better alignment with the study’s geographical context. We appreciate the reviewer’s suggestion, which has strengthened the regional relevance of our manuscript. Line 28 - 34.

New References:

1. Ministry of Health and Welfare (MOHW), Korea Disease Control and Prevention Agency (KDCA). 2022. Korea National Health and Nutrition Examination Survey (KNHANES) VIII-4, 2022. Sejong, Republic of Korea.

2. Ministry of Agriculture, Food and Rural Affairs (MAFRA). 2023. Food Consumption Trends and Agricultural Outlook in Korea. Sejong, Republic of Korea.

3. Lee JS, Kim J. 2021. Trends in fruit and vegetable consumption and dietary quality among Korean adults. Nutr Res Pract. 15:641–652. <https://doi.org/10.4162/nrp.2021.15.5.641>

Line 37 – The description of pesticide use and its impact on human health should be supported by more than two references to ensure sufficient scientific grounding.

**Response:** In the revised manuscript, we have expanded the discussion on pesticide use and associated human health impacts and have incorporated additional peer-reviewed references to strengthen the scientific grounding. These include recent global assessments and epidemiological studies demonstrating links between pesticide exposure and adverse health outcomes such as endocrine disruption, neurotoxicity, and chronic disease risks. The section has now been updated to provide a more comprehensive and well-supported overview in alignment with the reviewer’s suggestion.

**Additional References Included in the Revision:**

11. Mostafalou, S., & Abdollahi, M. (2017). Pesticides and human chronic diseases: Evidences, mechanisms, and perspectives. Toxicology and Applied Pharmacology, 329, 45–55.

12. Kim, K. H., Kabir, E., & Jahan, S. A. (2017). Exposure to pesticides and the associated human health effects. Science of the Total Environment, 575, 525–535.

13. Mnif, W., et al. (2011). Effect of endocrine-disrupting pesticides: A review. International Journal of Environmental Research and Public Health, 8(6), 2265–2303.

Line 42 – Avoid the statement that “pesticides are beneficial to agronomy.” Pesticides are necessary for maintaining yield stability but not inherently beneficial. Such phrasing is simplistic and repetitive. The introduction should be condensed and focused on identifying the scientific gap rather than reiterating general background information.

**Response:** We agree that the original phrasing was overly simplistic and did not accurately reflect the role of pesticides in agricultural systems. In response, we have removed the statement that “pesticides are beneficial to agronomy” and revised the text to provide a more precise explanation. The updated sentence now emphasizes the necessity of pesticides for maintaining yield stability rather than portraying them as inherently beneficial. We have also condensed surrounding content to improve clarity and to keep the introduction focused on defining the scientific gap. Line 49 - 51

Lines 46–55 - The discussion of cleaning and decontamination methods is unbalanced and contains inaccuracies. “Baking” and “freeze-drying” are not recognized or practical approaches for pesticide removal from fresh produce. The claim that “UV cleaning requires additional chemicals” is incorrect — UV treatments are non-chemical and based on photolytic degradation. The authors should reference peer-reviewed studies comparing ozone, plasma, and ultrasound methods in terms of degradation efficiency, safety, and limitations.

**Response:** Thank you for this helpful comment. We have substantially revised this section to improve clarity and focus, and the content has now been reorganized to better reflect the relevance of the food washing system. The revisions ensure that the discussion is clearly aligned with its applicability and significance to the topic. The revised paragraph can be checked in line 54-62.

Line 50 – More than four references are needed to substantiate claims regarding UV, ultrasound, ozone, and freeze-drying methods. The current text presents these methods as ineffective compared to MPW, although scientific evidence does not necessarily support such contrast

**Response:** Thank you for this insightful comment. We agree that the original description of decontamination methods was not sufficiently accurate and included methods (baking, freeze-drying) that are not practical for pesticide removal from fresh produce. We have removed these statements entirely.

In response to the reviewer’s suggestion, we have revised the section to provide a balanced, evidence-based overview of commonly studied decontamination methods, including **ozone, ultrasound, and plasma-based treatments**, supported by peer-reviewed literature. This revision improves accuracy and ensures that the introduction directly supports the rationale for investigating microplasma-treated water. Line 64 - 82.

References:

16. Gil MI, Selma MV, López-Gálvez F, Allende A. 2009. Fresh-cut product sanitation and wash water disinfection: Problems and solutions. *Int J Food Microbiol*. 134:37–45. https://doi.org/10.1016/j.ijfoodmicro.2009.05.021

17. Gómez-López VM, Ragaert P, Debevere J, Devlieghere F. 2009. Decontamination methods to prolong the shelf-life of minimally processed vegetables: A review. *Crit Rev Food Sci Nutr*. 49:473–491. https://doi.org/10.1080/10408390802067986

18. Allende A, Artés F. 2003. Combined ultraviolet-C and modified atmosphere packaging treatments for reducing microbial growth of fresh processed lettuce. *Food Sci Technol Int*. 9:47–52. https://doi.org/10.1177/1082013203009001007

19. Karaca H, Velioglu YS. 2007. Ozone applications in fruit and vegetable processing. *Food Rev Int*. 23:91–106. https://doi.org/10.1080/87559120600998221

20. Misra NN, Keener KM, Bourke P, Mosnier JP, Cullen PJ. 2014. In-package atmospheric pressure cold plasma treatment of food products. *Innov Food Sci Emerg Technol*. 23:168–175. https://doi.org/10.1016/j.ifset.2013.09.009

We appreciate the reviewer’s feedback, which has helped ensure that our discussion is balanced, well-referenced, and scientifically accurate.

Line 66-67: A clear explanation of MPW’s mode of action is necessary. The reader should understand what differentiates MPW from other plasma-based treatments (e.g., lower energy requirements, generation of reactive species in the aqueous phase, or better scalability).

**Response:** Thank you for this insightful comment. We agree that the original text did not sufficiently explain the mechanism of microplasma-treated water (MPW) or how it differs from other plasma-based decontamination methods. In response, we have revised this section to provide a clearer and more technically accurate explanation. The updated text highlights the unique features of MPW, the direct generation of reactive oxygen and nitrogen species (RONS) in the aqueous phase, and its potential for scalable and uniform treatment. Line 74 - 82.

Materials and methods

General comments: The reported pesticide concentration of 2000 ppm is unrealistically high compared with residue levels typically found on fresh produce. The authors should provide a clear justification for this choice and discuss how such an elevated concentration affects the environmental and practical relevance of their findings.

**Response:** We agree that 2000 ppm exceeds typical residue levels found on fresh produce. This concentration was intentionally selected for two specific reasons, which we have now clarified in the revised manuscript:

1. **Methodological Standardization:** High spiking levels are commonly used in degradation studies to ensure accurate quantification, minimize analytical variability at early time points, and enable clear monitoring of degradation kinetics across treatments.
2. **Stress-Testing the System:** The elevated concentration allows evaluation of the treatment’s capacity under worst-case or extreme contamination scenarios, which provides insight into the robustness and upper performance limits of the technology.

To address the reviewer’s concern about practical relevance, we have added a statement in the Discussion acknowledging that typical field residue levels are much lower and that real-world performance is expected to be similar or higher due to the lower pesticide burden. We also clarified that the purpose of the 2000 ppm condition is not to model actual consumer exposure but to provide a controlled, conservative test of degradation efficiency. These explanations have been incorporated into the revised Introduction (justification of experimental design) and Discussion (practical applicability).

Introduction part: Line 85 - 88; Discussion Part: Line 395 – 400.

The application of 2 mL of pesticide solution per sample lacks standardization relative to the produce surface area. For reproducibility, the authors should specify the size and surface area of each sample, describe the application method (spraying, pipetting, immersion), and confirm that deposition was consistent among replicates.

**Response:** We thank the reviewer for this important comment. In the revised manuscript, we have now clarified the dimensions and surface area of each produce sample, described the exact contamination procedure, and confirmed that pesticide deposition was standardized across replicates. Specifically, perilla leaves (2 × 2 inch sections) and broccoli florets (approximately 1 cubic inch pieces) were used for all trials. A fixed volume of 2 mL pesticide solution was applied to each sample using a calibrated micropipette, ensuring uniform distribution over the entire surface. All samples were handled under identical conditions to maintain consistency in pesticide deposition across replicates. These details have been added to the Methods section to improve reproducibility and methodological transparency.

The statement “pesticides were air dried for 2 h” is too vague. Drying conditions such as temperature, humidity, and airflow should be reported, as these parameters significantly influence pesticide adsorption and volatilization.

**Response:** We thank the reviewer for this valuable comment. In the revised manuscript, we have clarified the drying conditions to ensure reproducibility and transparency. Line 100 to 103.

The expression “at least three separate treatments were done” is ambiguous. The authors should clarify whether this refers to biological replicates (independent samples) or technical replicates (repeated measurements of the same extract).

**Response:** We thank the reviewer for pointing out this ambiguity. In the revised manuscript, we have clarified that the “three separate treatments” refer to **biological replicates**, meaning three independent samples processed separately through the full extraction and analysis workflow.

HPLC is an analytical technique, not an extraction method. The solvent extraction procedure should be described separately, including solvent type, volume, extraction time, agitation, and cleanup steps, followed by a clear explanation that pesticide quantification was performed by HPLC.

**Response:** We thank the reviewer for this helpful clarification. In the revised manuscript, we have now separated the extraction protocol from the analytical (HPLC) quantification method to ensure a clear distinction between the two procedures.

The solvent extraction method is now described in detail, including the type and volume of solvent used, sample mass, extraction time, agitation conditions, and any cleanup steps. This revision allows readers to fully understand how the analytes were isolated prior to instrumental analysis. Following the extraction section, we have clearly stated that pesticide quantification was performed using HPLC, and the analytical parameters of the HPLC method are described in a dedicated subsection. These revisions improve methodological clarity and align the manuscript with standard analytical reporting practices. Line 169 - 194.

While the preparation of a 2000 ppm pesticide stock solution in methanol is mentioned, essential details such as the purity of pesticide standards, storage conditions, and calibration curve preparation are missing. These are necessary for method reproducibility and analytical validation.

**Response:** We thank the reviewer for this important observation. In the revised manuscript, we have now provided complete details regarding the preparation and handling of the pesticide stock solution. Specifically, we have included:

1. **Purity of pesticide standards** (98% pure, sourced from Sigma Aldrich, S. Korea).
2. **Storage conditions**, including temperature, light protection, and stability period.
3. **Preparation of calibration curves**, including concentration range, solvent, number of calibration points, and replication for linearity assessment.

Can be found in line 177 - 194.

Terms like “function producer” and “current stimulator” are non-standard and should be replaced with “function generator” and “power amplifier.” Likewise, phrases such as “elevated voltage swapping converter” are unclear and should be revised using proper electrical engineering terminology.

**Reply:** It was replaced by the standard term according to the suggestion. The entire paragraph was revised substantially. Can be found in line 106 - 122.

Although the electrical configuration is described in detail, there is no linkage between operational parameters and the physicochemical characteristics of the treated water (e.g., concentration of reactive oxygen/nitrogen species, pH, oxidation–reduction potential). Quantitative data should be presented to substantiate the claimed treatment intensity.

**Response:** We appreciate the reviewer’s comment regarding the linkage between operational parameters and the physicochemical characteristics of the treated water. While direct numerical values are not tabulated, quantitative information is provided in graphical form for the real produce application. Selected sample was broccoli (Figures 3 – 5). These figures present the measured concentrations of reactive species and related physicochemical parameters under different operating conditions, thereby illustrating their dependence on the applied electrical parameters. The use of graphical representation was chosen to clearly demonstrate trends and comparative behavior rather than isolated data points, which we believe more effectively conveys treatment intensity and its impact on water chemistry. Nevertheless, if the reviewer considers it necessary, we would be pleased to include tabulated numerical data in the revised manuscript or Supporting Information.

The description of the optical emission spectroscopy (OES) setup is overly general and lacks critical parameters required for reproducibility. Essential information such as spectral range (nm), integration time, spectral resolution (Δλ), number of averaged scans, and identification method for emission lines (e.g., OH, NO, N₂, O) should be included. The manuscript mentions that spectra were recorded, but no representative OES spectra or emission peak identifications are provided. Without these data, claims regarding reactive species generation remain unsubstantiated.

## ****Response:**** We thank the reviewer for this constructive comment. We agree that additional detail on the optical emission spectroscopy (OES) setup is necessary for reproducibility. In the revised manuscript, we have now included all essential parameters of the OES configuration, including spectral range, integration time, spectral resolution (Δλ), number of averaged scans, and the procedure used for identifying major emission lines (OH, NO, N₂, O, etc.). Furthermore, we have added a representative OES spectrum in the Supplementary Material (Fig 1S) to document the characteristic reactive species generated under our discharge conditions. Although similar spectra were previously published in our earlier work on the same plasma system, we now provide the relevant diagram here to ensure completeness and transparency for this study. We have also updated the Methods section to explicitly describe the analytical steps used for peak identification and interpretation. These additions substantiate the claims regarding reactive species generation and enhance the reproducibility of the methodology. Please check the **lines 127-136**.****

Misuse of terminology should be corrected throughout (e.g., “inoculated” should not be used to describe pesticide application).

**Reply:** It is corrected and replaced by Contaminated.

Line 76 - The term “pesticide inoculation” is not appropriate in this context. The word “inoculation” is typically reserved for introducing microorganisms or biological material (e.g., bacterial inoculation, viral inoculation). Since the experiment involved the deliberate application of pesticide solutions onto plant surfaces, a more accurate expression would be “pesticide application”,

**Reply:** Done and mentioned previously.

line 68–70 - The authors cite a previous study reporting 97–99% degradation within 1–3 minutes. If such high efficiency was already achieved, the novelty of the current work must be clearly defined — what new question or improvement does this study address? Otherwise, the manuscript risks being perceived as confirmatory rather than innovative.

**Response:** We thank the reviewer for this important observation. The previous study referenced (our earlier work) investigated **pesticide degradation in distilled water only**, where mass transfer limitations are minimal. The current study, however, addresses a **significantly more complex and practically relevant scenario**—the degradation of pesticides applied directly onto **real food matrices** (fresh produce). Unlike water, food surfaces present additional challenges such as irregular microstructures, waxy cuticles, variable porosity, and protective surface chemistry that can hinder plasma–pesticide interactions.

Therefore, the novelty of the present work lies in:

1. **Application of plasma treatment to pesticide residues on actual food items**, not in aqueous solutions.
2. **Evaluation of degradation efficiency on complex surfaces**, which has not been reported in our previous publication.
3. **Demonstration that effective degradation (> 98%) is still achievable even under the more demanding conditions** of food matrices when treated for 4 minutes.
4. **Providing practical applicability**, since food contamination—not aqueous pesticide solutions—is the real-world problem.

We have revised the manuscript to clearly highlight this distinction and strengthen the presentation of the study’s novelty. Line 82-85 and line 359-362.

Line 87 - The description of the microplasma system is highly dependent on prior publications and therefore not self-contained or reproducible. All critical experimental parameters should be reported within the manuscript itself, not by reference only.

**Response:** We appreciate the reviewer’s concern. Although the microplasma system used in this study is based on the same platform as our previously published work, the **objective and experimental matrix in the current study are completely different**. The previous publication focused on **standard pesticides in aqueous solution**, whereas the present work investigates **pesticide residues on real produce**, which involves different treatment challenges and practical applicability.

In response to the reviewer’s comment, we have revised the Materials and Methods section to ensure **all critical experimental parameters are fully described within the manuscript itself**, including:

- detailed device geometry
- operating voltage and frequency
- gas flow rate
- electrode gap and configuration
- treatment distance
- discharge characteristics

These parameters are now explicitly presented so that the experimental setup is **fully reproducible without relying on external references**. Line 106-121.

Line 88- The authors state that an “improved microplasma generator” was used, yet no technical improvements are actually described. It remains unclear what was modified relative to the previous design (geometry, power supply, discharge frequency, or cooling system). The term “improved” should be replaced with a quantitative or descriptive explanation of specific design changes.

**Response:** We thank the reviewer for this valuable comment. We would like to clarify that the microplasma generator used in this study was not an improved or modified version of the previously reported device. The generator design, including geometry, power supply, discharge configuration, and cooling system, remained unchanged from our earlier work. In the present study, only the operating parameters (i.e., duty cycle and discharge frequency) were optimized to achieve effective treatment, as explicitly described in the Materials and Methods section. To avoid confusion, the term “improved” has been removed and replaced with a more accurate description in the revised manuscript. Line 107 to 109.

Line 94 - Several values are confusing or imprecise. For example, “the working energy was about 30 kV” should refer to voltage, not energy. Units such as “W/h” for power consumption are incorrect; power should be expressed in watts (W), not watt-hours per hour. Please review and correct all physical quantities and units.

**Response:** We thank the reviewer for pointing out these important issues regarding terminology and units. In the revised manuscript, we have carefully reviewed all physical quantities and corrected them as follows:

1. The term “working energy” has been corrected to **“ouput voltage”**, with the value expressed as **30 kV**.
2. Power consumption units have been corrected from “W/h” to **watts (W)**, and all related descriptions of energy or power have been verified for accuracy.
3. All other physical parameters and units in the manuscript have been reviewed and corrected to ensure scientific precision and consistency.

These changes have been incorporated into the Materials and Methods section (**Generation of microplasma treated water**) to improve clarity, accuracy, and reproducibility.

Line 112 - Reference [26] is cited for “further evidence,” - complete OES method should be described within the paper. A reader should not have to consult earlier work to reproduce the spectral measurements.

**Response:** We thank the reviewer for this important observation. In the revised manuscript, we have expanded the Materials and Methods section to provide a complete and self-contained description of the Optical Emission Spectroscopy (OES) procedure. The updated text now includes details on instrument model, operating parameters, plasma conditions, spectral range, resolution, integration time, calibration approach, and data processing steps in the section named **Optical emission spectroscopy.** While Reference [26] is still cited for contextual support, all methodological information required to reproduce the spectral measurements is now fully described within the present manuscript, eliminating the need for readers to consult earlier work. Line 127 -136.

Line 113 – Section determination acks the quantitative and procedural detail necessary to validate the reported radical concentrations. Authors must include calibration information, replicate numbers, mean ± SD values, and a clear explanation of how each reactive species contributes to the oxidative capacity of MPW.

**Response:** We thank the reviewer for this valuable comment. In the revised manuscript, we have substantially expanded the radical determination section to provide full methodological transparency and quantitative detail.

Specifically, we have now included:

1. **Calibration information**, including the standard compounds used, calibration curve range, regression equations, and R² values.
2. **Replication details**, stating the number of biological and technical replicates performed for each radical species.
3. **Quantitative results reported as mean ± SD**, clearly presented for all measured radicals.
4. A **clear explanation of the relative contribution of each reactive species** (e.g., •OH, O_3_, H₂O₂, NO_x_ etc.) to the overall oxidative capacity of MPW, supported by literature and our experimental observations.

These additions ensure that the determination of radical concentrations is reproducible, properly validated, and scientifically interpretable. All updates have been incorporated into the Materials and Methods and the Results sections, with supporting data included in the Supplementary File Text 1S, text 2S and Text 3S.

Line 135- 142 - The extraction and HPLC quantification steps are described, but no method validation data are presented. Recovery rate, linearity, limits of detection (LOD) and quantification (LOQ), and repeatability are all essential for assessing analytical reliability. Without these, the reported degradation efficiencies are not scientifically defensible. Using 20 mL of methanol for extraction without specifying sample mass or surface area prevents any quantitative comparison between treatments. Extraction efficiency depends heavily on sample-to-solvent ratio, which must be stated.

**Response:** We thank the reviewer for this important observation. In response, we have now included full validation data for the HPLC method to ensure the analytical reliability of the reported results. The revised manuscript (**Preparation of stock solutions and analytical calibration methods section line 177 to 194** and Supplementary File, Text S7) now presents the method’s linearity, recovery rate, limits of detection (LOD), limits of quantification (LOQ), and repeatability (RSD%). These data confirm that the method is robust and suitable for quantifying the analytes. Line

Although the instrument and column are named, critical chromatographic parameters are missing — retention time, run time, chromatogram validation, calibration curve details, and use of standards. No information is provided on how chlorpyrifos was analyzed (different wavelength or mobile phase?). The so-called “negative control” (device switched off for 4 min) does not represent a valid procedural blank. Proper controls should include (i) untreated samples spiked with pesticide but not exposed to MPW, and (ii) solvent-only blanks to account for potential losses during extraction and handling. The section does not specify number of replicates, variability (SD), or statistical tests applied to analytical results.

**Reply:** All mentioned in the section “**Preparation of stock solutions and analytical calibration methods lines 177 - 194”.**

The methods describe diazinon analysis only; chlorpyrifos is mentioned in the introduction but lacks any chromatographic or detection details here. The protocol must be expanded to include both pesticides explicitly.

**Reply:** Included in the revised manuscript. Line 199 - 206.

Line 145-155 - The description of color measurement lacks essential methodological details such as measurement geometry, lighting conditions, and sampling procedure. Terminology is incorrect (the device is a spectrophotometer, not a “camera”. Biological replication and statistical analysis of color changes are also missing.

**Response:** We appreciate the reviewer’s insightful comments. The color measurement methodology has now been thoroughly revised to include the required technical specifications. We have added details regarding **instrument geometry (d/8°), illuminant (D65), observer angle (10°), calibration procedure, sampling method, and replication strategy**. The incorrect term “camera” has been replaced with “spectrophotometer.” We have also clarified that **five independent measurements** were taken at randomly selected points for each sample, and we now explicitly refer to the **statistical analysis** used to compare color changes between treatment groups. These revisions significantly improve clarity and methodological completeness (Revised Manuscript: Lines 214–216).

Line 162 – Why did authors choose the Mann–Whitney U test for multi-factorial experiments involving multiple treatments and variables? ANOVA with post hoc testing would be more suitable.

**Response:** “We appreciate the reviewer’s comment and ANOVA with post hoc testing was done for all the measurements.

Results
Results section is descriptive rather than analytical, lacking quantitative rigor, validation, and appropriate statistical evidence to substantiate the conclusions.

**Response:** Thank you for this important observation. In the revised manuscript, the Results section has been substantially strengthened. We have:

1. **Added quantitative statistical analyses**, including mean ± SD for all data points, replicate numbers (n ≥ 3), and significance testing (ANOVA with post-hoc comparisons).
2. **Revised the presentation of results** to emphasize analytical interpretation rather than descriptive statements. Corresponding revisions appear in the updated Results section (Lines 260–263, 307 – 313, and 322 - 331).

Findings are merely restated from previous work, and no actual OES spectra or peak identification are shown; thus, claims about reactive species formation remain unsupported within this manuscript.

**Response:** We appreciate this comment. To address it:

1. We have now **included representative OES spectra as Supplementary Figure S1**, with full peak identification for major reactive species (e.g., OH•, N₂*, O*, NO). Comprehensive description is included in the section **Optical emission spectroscopy line 127 - 136.**
2. The revised text in the Results section now **directly refers to these spectra** rather than restating earlier publications.
3. All statements regarding reactive species are now supported by **specific peak assignments and wavelengths** (Lines 260 – 263). This ensures the manuscript contains original and verifiable analytical data.

Results show apparent high degradation, but analytical validation (recovery, LOD/LOQ, calibration) and replicate variation are missing, making the numerical efficiencies unreliable;

**Response:** Thank you for highlighting the need for analytical validation. We have now incorporated the following:

1. **Recovery results** obtained using spiked blank matrices (Supplementary Table S1, Text S7).
2. **Replicate variability (n = 3–5)** for each treatment condition, reported as mean ± SD.
3. A statement clarifying that **all quantification was performed using validated HPLC protocols**. These elements have been added in both the Methods and Results sections (Lines 182–194).

Interpretations about ozone and NOx behavior are speculative since no direct chemical measurements are provided.

**Response:** We thank the reviewer for this comment. In the revised manuscript, we have now included additional information regarding the effects of O₃ and NOₓ, which were determined using a **passive measurement technique**. These results allow a more substantiated interpretation of the behavior of these species. Further methodological details and supporting data have been added to the **Supplementary Information**, including **Figure S2** and **Text S1 and Text S4**, where the measurement approach and related discussion are described in detail.

Line 271- 273 and Line 429-433.

Disscussion
The discussion restates much of the results and previously published data without offering deeper analysis or critical interpretation.

**Response:** We thank the reviewer for this comment. In the previous work, we demonstrated the degradation efficiency and mechanism of the standard pesticide under controlled conditions. In this study, we applied the same approach to real field samples, and while the degradation mechanism is expected to be consistent with our previous findings, the focus here is on demonstrating efficacy under practical conditions. We have added a sentence in the revised manuscript to clarify this point, as mentioned elsewhere in this rebuttal letter.

The mechanistic explanations for pesticide degradation (roles of NOx, O₃, and OH) are largely speculative, as no direct experimental confirmation was presented in this study.

**Response:** We would like to clarify that experimental efforts to elucidate the roles of individual reactive species were conducted using a passive scavenging approach, and the corresponding results are provided in the Supplementary Information (Text S6 and Fig. S2). In this analysis, selective suppression of specific reactive species (NOₓ, O₃, and OH radicals) allowed assessment of their relative contributions to pesticide degradation. The observed changes in degradation kinetics under different scavenging conditions provide experimental support for the proposed degradation mechanisms. To improve clarity, we have explicitly referred to these supplementary results in the main text. Line 429-433.

Comparisons with other plasma systems are descriptive and lack quantitative benchmarking or statistical context.

**Response:** We appreciate the reviewer’s comment regarding quantitative benchmarking. However, it should be noted that all previous studies have employed fundamentally different approaches, making direct quantitative comparisons impractical. Each study is unique in terms of the plasma devices used, the types of produce treated, and the specific pesticides targeted. Our descriptive comparison is intended to highlight these differences and to show that no prior work has addressed the combination of factors investigated in our study. Therefore, while statistical benchmarking across studies is not feasible, our discussion effectively demonstrates that our work represents a distinct contribution in this field. We added a statement clarifying the limitations of comparing heterogeneous plasma systems. Revised text appears in lines 422 - 426.

Claims about “no significant chemical changes” and “non-toxicity of degradation products” are unsupported by analytical evidence, since no identification or toxicity testing of byproducts was performed.

**Response:** We fully agree and have corrected this.

1. All claims regarding chemical safety or the absence of harmful byproducts have been removed or rewritten.
2. We now explicitly acknowledge that **no byproduct identification, toxicity assessment, or LC-MS profiling** was conducted in this study.
3. A limitations paragraph has been added clearly stating that **toxicity and safety conclusions cannot be drawn without additional analysis**.
   These changes eliminate unsupported claims (Lines 512–523).

The discussion also mixes methodological details (e.g., drying conditions, moisture content) that belong to the Materials and Methods section.

**Response:** All methodological elements (e.g., drying conditions, moisture content, treatment handling) have been moved to the **Materials and Methods** section. The Discussion now focuses exclusively on interpretation of results. Updated in Lines 381 – 385.

Overall, this section should focus on data interpretation supported by the study’s own findings, include mechanistic reasoning consistent with measured parameters, and acknowledge limitations such as unrealistic pesticide concentrations and lack of residue confirmation in treated water.

**Response:** We appreciate this suggestion. The revised manuscript now includes a **clear limitations subsection (line 512 - 523)**, acknowledging:

1. The use of controlled pesticide concentrations that may not fully represent field-level contamination.
2. The absence of **confirmation of pesticide residues or byproducts in treated water**.
3. The lack of comprehensive analytical identification for degradation intermediates.
4. The need for scalability and real-sample validation.

# **Summary of Revisions Made to the Discussion**

- Added analytical depth and avoided repetition: Line 358 - 369
- Removed or rephrased speculative mechanistic statements. Line 435 – 444 and line 446 - 451
- Added quantitative comparisons for temperature effect. Line 455 - 471
- Removed unsupported claims about safety and chemical changes.
- Moved methodological content to the Methods section.
- Added a comprehensive limitations subsection.
- Ensured interpretation remains aligned strictly with data generated in this study.

**References deleted from previous manuscript:**

**No.**

**1**

**6**

**7**

**8**

**11**

**12**

**14**

**15**

**16**

**23**

**24**
