# Supplementary material for: Removal efficiency of pesticide residues on pesticide-spiked Perilla Leaf and Broccoli surfaces using microplasma-treated water
Source: PLoS One. 2026 Jun 26;21(6):e0351955. doi: 10.1371/journal.pone.0351955 (PMC13309029; doi:10.1371/journal.pone.0351955)
Supplement: Attachment — Submitted filename: Response to Reviewer R2.docx [file pone.0351955.s006.docx]

We sincerely **thank** the Editor and reviewers for their careful evaluation of our manuscript and for the constructive comments and suggestions. These insights were extremely helpful and have significantly improved the clarity, quality, and scientific rigor of the manuscript. All comments have been carefully considered and addressed in detail below.

**Additional Editor Comments:**

The manuscript needs major revision with respect to the reviewers comments and following comments. Authors may revise and submit the revised manuscript with authors response to each comment.
The manuscript study focused pesticide degradation analysis on the spiked samples not contaminated samples. So, please avoid “contaminated produce surface” and may write specific term throughout the manuscript including manuscript title. Or, justify with relevant literature.

**Reply:** “Pesticide-spiked” word is used in throughout the manuscript.

With respect to the study objective (Line 72) and obtained results, lacking of methodology about the chlorine washing treatment on the pesticide spiked samples. Please check and add methodology in the materials and method section.

**Reply:** Thank you for your valuable comment. We agree that the description of the chlorine washing treatment was insufficient in the original manuscript. Accordingly, a detailed methodology for the chlorine washing of pesticide-spiked samples has now been added to the Materials and Methods section (Lines 172–177).

Lacking of statistical representation in all the presented results data. Please check and add statistical significance representation in the data.

Reply: Added!

Line 32: Which standard agency or regulatory body guidelines? Specify.

Reply: Line is modified as KNHANES 2022 reports that over 72% of adults consume fresh vegetables regularly, which aligns with the **Korean Dietary Guidelines issued by the Ministry of Health and Welfare** promoting increased vegetable intake [1] which Specified the regulatory body.
Line 68-69: With reference to the cited reference Hernandez-Borges et al., (2009), please check the sentence “We previously showed that one ……………………..” and revise with correct reference.
Reply: This one will be reference 26.

Line 77: Add scientific name of perilla leaf and broccoli.

Reply: Included.

Line 77-78: How the pesticide free produce were confirmed/ authenticated?

Reply: Thank you for your insightful comment. The produce samples used in this study were procured from a government-regulated fresh produce market in Korea that certifies products as organic. We acknowledge that “organic” does not necessarily guarantee the complete absence of pesticide residues. Therefore, the manuscript has been revised to clarify that the samples were **organically sourced produce** rather than analytically confirmed pesticide-free samples (Line 95).

Line 79: How much quantity of samples were treated?. Specify.

Reply: Mentioned in the revised manuscript (Lines 97-98).

Line 80-81: Move the sentence “Pesticide samples were obtained from Sigma-Aldrich, South Korea” to before the sentence “The surfaces of the produce ………………”.

Reply: It is now shifted in relevant paragraph.

Line 80: Add purity and sample code of pesticide standards (diazinon and chlorpyrifos) of Sigma-Aldrich.

Reply: Added!! Line 202-203.

Line 82: Why the higher concentration “2 ml of 2000 ppm pesticide solution” was used in the study?. Justify with relevant literature.

*Response:* We thank the reviewer for this valuable observation. Pesticide spiking was performed by applying 2 mL of a 2000 ppm solution to each treatment batch (3 g perilla leaf; 200 g broccoli), resulting in a uniform residue level in the range of 0.1–0.5 µg/g, in accordance with SANTE/11312/2021 guidelines to ensure environmental relevance. We have also added a statement acknowledging that degradation kinetics at higher initial concentrations may differ from those observed under realistic conditions. These revisions improve the clarity and practical applicability of our findings. The corresponding changes have been incorporated in lines 87–91, lines 103 – 106 and lines 545-546.

Line 84: Why “At least”?. Please specify.

Reply: Sentence was re-written. Three independent treatments of each sample were performed using microplasma prior to HPLC analysis to ensure reproducibility and statistical reliability of the results.

Line 129: Why residual analysis performed for diazinon only not for another pesticide?. Please check the sub-heading and explain in the methodology.

Reply: Chlorpyrifos detail is included in the section **Preparation of stock solutions and analytical calibration methods.**

Line 146: In which samples, colour changes analysis were performed?. Specify

Reply: Specified in line 241-244.

Line 368-370: Please remove the sentences “To the best of our knowledge, ………………………… treated produce”, not required.

Reply: Removed.

Line 370: Avoid “our” and may write “In the present study, microplasma discharge water system performed ……………………….”.

Reply: Done!

**6. Review Comments to the Author**

Please use the space provided to explain your answers to the questions above. You may also include additional comments for the author, including concerns about dual publication, research ethics, or publication ethics. (Please upload your review as an attachment if it exceeds 20,000 characters).

Reviewer #1: The authors have diligently addressed most of the concerns raised in the previous review round. The manuscript has been significantly improved in terms of experimental design and data presentation. However, two critical issues require further clarification to ensure the robustness of the conclusions and the practical interpretation of the results.
1. Potential Interference from Residual Active Species During Ultrasonication
Comment:
In the revised manuscript, the authors state that reactive nitrogen species (RNS) are the primary agents responsible for pesticide degradation and that these species are long-lived in the system. This raises a valid concern: could residual RNS present on the treated produce surface continue to degrade pesticides during the subsequent ultrasonication step used for extraction? This potential ongoing degradation would conflate the effect of the microplasma-treated water wash with the effect of ultrasonication-assisted degradation, thereby overestimating the efficiency attributed solely to the plasma-treated water.

*Response:* We thank the reviewer for this insightful comment. We agree that residual reactive nitrogen species (RNS) could potentially contribute to further degradation during extraction. To address this concern, samples were immediately transferred to methanol following treatment. Methanol acts as an effective quenching and extraction solvent, rapidly dissolving pesticide residues while minimizing continued reactions from residual reactive species. Additionally, ultrasonication was conducted under controlled temperature conditions (22 ± 1°C) to avoid any secondary degradation effects. We have clarified this point in the revised manuscript (**Pesticide extraction methods**), emphasizing that the ultrasonication step was intended solely to enhance extraction efficiency rather than induce degradation. Line 189-185 and line 331-334.

2. In lines 383-393, the authors cite previous work showing ~99% degradation of pesticides in standard solution within 3 minutes of direct microplasma treatment. They use this to support the efficacy of their process. However, a crucial analytical step is missing in the current study: the quantification of pesticide residues in the rinse water after it has been used to wash the contaminated produce.

The efficiency of pesticide degradation in a standard solution under direct plasma exposure can differ significantly from the efficiency of removal and degradation in a complex matrix like produce wash water. Pesticides may be partially transferred from the produce surface into the wash water without being fully degraded. Therefore, relying solely on prior standard solution data is insufficient to claim complete degradation in the applied context.

*Response:* “While pesticide residues in rinse water were not directly measured, the 4-minute plasma treatment exceeds the 3-minute degradation time established in our previous studies for standard solutions. Therefore, we reasonably expect that any pesticides transferred to the wash water were effectively degraded. A statement clarifying this assumption has been added to the Methods and Discussion sections (Lines 180 – 186). And in the discussion part line 418 to 423.

Reviewer #2:

Dear authors, The revised manuscript shows improved clarity and structure, and many of the comments from the first review have been addressed. The study addresses a relevant topic and the experimental approach is of potential practical interest. However, several methodological aspects would benefit from further clarification to ensure analytical transparency and reproducibility.

In particular, although additional information on analytical validation, statistical analysis, and plasma diagnostics has been provided, some key methodological details remain insufficiently summarized in the main text. The environmental relevance of the pesticide concentration used (2000 ppm) should also be discussed more explicitly.

Specific comments

1. Analytical validation of the HPLC method

Validation parameters are reported in the Supplementary Information. It would be helpful to briefly summarize the key values (recovery range, R², LOD and LOQ) in the main manuscript to facilitate evaluation of the analytical reliability.

*Response:* “We appreciate the reviewer’s suggestion. Key validation parameters have now been summarized in the main text (Methods section). Line 214-216

2. Control experiments

The description of control conditions could be clarified further. In particular, please specify whether pesticide-spiked produce washed with untreated water was included as a control to distinguish degradation from simple washing effects.

*Response:* “Produce washed with untreated water was included as a control in all experiments to distinguish the effect of simple washing from plasma-assisted degradation. This has been clarified in the Methods section.” Line 108-109.

3. Environmental relevance of pesticide concentrations

The use of a 2000 ppm pesticide concentration is justified by the authors; however, this value is considerably higher than typical residue levels on fresh produce. The Discussion should more clearly acknowledge how this may affect degradation kinetics and the applicability of the results to realistic scenarios.

*Response:* We thank the reviewer for this valuable observation. Pesticide spiking was performed by applying 2 mL of a 2000 ppm solution to each treatment batch (3 g perilla leaf; 200 g broccoli), resulting in a uniform residue level in the range of 0.1–0.5 µg/g, in accordance with SANTE/11312/2021 guidelines to ensure environmental relevance. We have also added a statement acknowledging that degradation kinetics at higher initial concentrations may differ from those observed under realistic conditions. These revisions improve the clarity and practical applicability of our findings. The corresponding changes have been incorporated in lines 87–91, lines 103 – 106 and lines 545-546.

4. Statistical analysis

Please clarify the statistical model applied, the factors included in the analysis (e.g., washing method, pesticide type, temperature), and the number of biological replicates used in each treatment.

*Response:* “Statistical analysis was performed using ANOVA, with factors including washing method, pesticide type, and temperature. A minimum of three biological replicates per treatment were included, and in some cases up to five replicates. This has been clarified in the Methods section.”
5. Mechanistic interpretation

**Reviewer Comment:**

Additional plasma diagnostics are useful, but the mechanistic interpretation remains partly indirect. The discussion should clearly distinguish between experimentally measured parameters and interpretations based on previous studies.

**Response:** We sincerely thank the reviewer for this valuable comment. We agree that clearly distinguishing between experimentally measured results and literature-based interpretations is important. In fact, this distinction was already indicated in the original manuscript. For example, in Discussion Section (Lines 458– 485), we state: *“Detailed methodologies and analyses used to elucidate the roles of individual reactive species are provided in Text S6 and Fig. S2.”* This was intended to clarify that while reactive species (e.g., OH radicals, RNS, H₂O₂, and pH changes) were experimentally quantified in the present study, the specific roles and relative contributions of individual species in pesticide degradation are inferred based on our previous work and other published studies, as appropriately cited. However, we acknowledge that this distinction may not have been sufficiently explicit. Therefore, we have revised the relevant paragraph in the Discussion section to more clearly separate (i) findings obtained directly from the current study and (ii) mechanistic interpretations supported by prior studies.
