# Supplementary material for: Removal efficiency of pesticide residues on pesticide-spiked Perilla Leaf and Broccoli surfaces using microplasma-treated water
Source: PLoS One. 2026 Jun 26;21(6):e0351955. doi: 10.1371/journal.pone.0351955 (PMC13309029; doi:10.1371/journal.pone.0351955)
Supplement: Attachment — Submitted filename: Response to Reviewer R3.docx [file pone.0351955.s007.docx]

We sincerely **thank** the Editor and reviewers for their careful evaluation of our manuscript and for the constructive comments and suggestions. These insights were extremely helpful and have significantly improved the clarity, quality, and scientific rigor of the manuscript. All comments have been carefully considered and addressed in detail below.

Additional Editor Comments:

With the reviewer comments, the revised manuscript is improved compared to previous version. Though, the manuscript needs revision with respect to the reviewer comments and following comments.
Line 1-2: In the manuscript title, write like “……. on pesticide-spiked surfaces using ……” instead of “……… on pesticide-spiked produce surfaces using ………”.

Reply: Changed

Line 15: Write like “Pesticide-spiked food produce surfaces …..” instead of “Pesticide-spiked Produce surfaces …..”.
Reply: Wrote.

Line 20-21: Remove the SD values “(± 2.3–3.1%), (± 2.8–3.6%)” and “(mean ± SD, n = 3)”.

Reply: Removed

Please check the typographical and grammatical errors throughout the manuscript.

Reply: Checked carefully and corrected.

However, a few points would still benefit from clarification:

1. Distinction between pesticide removal and degradation

The study refers to “degradation efficiency,” but the current design does not clearly distinguish between physical removal (transfer to washing solution) and chemical degradation. As residues in the rinse water were not measured, the reported effects may partly reflect removal rather than degradation. This should be more explicitly acknowledged as a limitation, or the interpretation adjusted accordingly.

**Reply:** Thank you for this important observation. We agree that the current experimental design does not fully distinguish between physical removal (transfer into the washing solution) and actual chemical degradation. In our study, the reduction in pesticide residues on the sample surface may indeed result from a combination of both processes. As the pesticide content in the washing solution was not quantified, we cannot definitively separate these contributions. We have now explicitly acknowledged this as a limitation in the revised manuscript and adjusted the wording from “degradation efficiency” to “removal/degradation efficiency” and sometimes removal and/or degradation efficiency where appropriate to avoid overinterpretation. It was acknowledged in the limitation section line 447 to 449.

2. Potential artefacts during extraction

The use of methanol and controlled temperature during extraction is noted. Given the presence of relatively long-lived reactive species, some continued degradation during ultrasonication cannot be fully excluded. A brief statement acknowledging this as a potential limitation would improve transparency.

**Reply:** We appreciate this insightful comment. Although methanol extraction and controlled temperature conditions were employed to minimize post-treatment reactions, we acknowledge that the presence of relatively long-lived reactive species may lead to continued degradation during ultrasonication. This possibility has now been included as a limitation in the revised manuscript to ensure transparency. *“Despite controlled extraction conditions, the possibility of continued degradation due to residual reactive species during ultrasonication cannot be completely excluded.”* Is included in the materials and methods section line 195 to 197.

3. Environmental relevance of pesticide concentration

Although the authors provide a justification for using a 2000 ppm stock solution and relate the resulting residue levels to SANTE guidelines, the explanation remains somewhat unclear and may be misleading for readers. A short note on how this setup relates to real-world conditions would strengthen the interpretation.

Reply: Thank you for this valuable comment. We agree that clarification of the environmental relevance of the pesticide concentrations is important. The high concentration (2000 ppm) refers only to the stock solution used for accurate preparation of working solutions and does not represent the actual residue levels on the produce. The final spiking levels applied to the samples (0.1–0.5 µg/g) were selected in accordance with SANTE/11312/2021 guidelines and are within ranges reported for real-world residue levels. To avoid misunderstanding, we have added a clarifying statement in the Introduction section (Line 92 - 95) and in the limitation part line is polished (541-542).

4. Statistical analysis

The inclusion of ANOVA is appropriate, however, a brief clarification of the statistical model, post-hoc testing (if applied), and number of replicates per treatment would improve reproducibility.

Reply: Paragraph is modified to address the query, line 253-260.
