# Supplementary material for: Removal efficiency of pesticide residues on pesticide-spiked Perilla Leaf and Broccoli surfaces using microplasma-treated water
Source: PLoS One. 2026 Jun 26;21(6):e0351955. doi: 10.1371/journal.pone.0351955 (PMC13309029; doi:10.1371/journal.pone.0351955)
Supplement: Attachment — Submitted filename: Response to Reviewer R4.docx [file pone.0351955.s008.docx]

We sincerely thank the reviewer for the thorough and highly constructive evaluation of our manuscript. We particularly appreciate the detailed and precise suggestions provided, which have been extremely helpful in improving the clarity, consistency, and scientific interpretation of our work. All comments have been carefully addressed, and the manuscript has been revised accordingly. We believe these revisions have strengthened the overall quality of the manuscript and ensured that all conclusions are fully supported by the experimental data.

6. Review Comments to the Author

Please use the space provided to explain your answers to the questions above. You may also include additional comments for the author, including concerns about dual publication, research ethics, or publication ethics. (Please upload your review as an attachment if it exceeds 20,000 characters)

Reviewer #2: Dear authors,
the manuscript has improved substantially and now meets the general standards of PLOS ONE in terms of methodological transparency, structure, and clarity. The authors have addressed most of the previously raised concerns in a satisfactory manner.
Before final acceptance, I recommend one minor but important revision related to the interpretation of the results:
While the distinction between pesticide removal and degradation is now acknowledged and the terminology has been adjusted accordingly, some statements in the manuscript still imply effective or near-complete degradation in the washing system based on prior studies in standard solutions. In particular, assumptions regarding the absence of pesticide residues in the wash water are not directly supported by the experimental data presented in this study.
I recommend moderating the wording in the following instances to avoid overinterpretation:

Line 182: “The 4 min exposure … ensures that any pesticides transferred to the wash water are effectively degraded.”
The term “ensures” is too strong and not methodologically justified.
Suggested: “The 4 min exposure exceeds the degradation time observed in standard solutions; however, degradation of pesticides transferred to the wash water was not directly verified under the present experimental conditions.”

Reply: Accepted the suggestion and modified accordingly.

Line 184: “ultrasonication … is unlikely to result in additional degradation attributable to residual reactive nitrogen species (RNS)”
This statement is inconsistent with the acknowledged limitation.
Suggested: “Ultrasonication may still allow limited additional degradation due to residual reactive species, which cannot be fully excluded.”

Reply: Accepted the suggestion and modified accordingly.

Line 380: “comparable behavior was observed in real field samples, ensuring its effectiveness.”
“Ensuring” implies confirmation beyond the presented data.
Suggested: “Comparable trends were observed in produce samples, supporting the effectiveness of the treatment in reducing surface residues.”

Reply: Accepted the suggestion and modified accordingly.

Line 416: “Therefore, it may be reasonably assumed that the wash water did not contain significant amounts of undegraded pesticides.”
This is a direct assumption without experimental verification.
Suggested: “However, the presence of pesticide residues in the wash water was not quantified and cannot be excluded.”

Reply: Accepted the suggestion and modified accordingly.

Line 554: “MPW system performed well at removing and/or degrading pesticides…”
This still implies confirmed degradation.
Suggested: “MPW system performed well in reducing pesticide residues (through removal and/or degradation) on produce surfaces.”

Reply: Accepted the suggestion and modified accordingly.

This is a matter of wording and interpretation rather than additional experimentation, but it is important to ensure that the conclusions remain fully supported by the data. With these minor clarifications, the manuscript would be suitable for publication. With my best regards.
