# Supplementary material for: Removal efficiency of pesticide residues on pesticide-spiked Perilla Leaf and Broccoli surfaces using microplasma-treated water
Source: PLoS One. 2026 Jun 26;21(6):e0351955. doi: 10.1371/journal.pone.0351955 (PMC13309029; doi:10.1371/journal.pone.0351955)
Supplement: Peer review history

# Peer review: Decision Letter 0

**DOI:** 10.1371/journal.pone.0351955.r001

**Authors:** S Ezhil Vendan

6 Nov 2025

PONE-D-25-44051

Degradation efficiency of pesticide residues on contaminated produce surface using microplasma treated water

PLOS ONE

Dear Dr. Khan,

Thank you for submitting your manuscript to PLOS ONE. After careful consideration, we feel that it has merit but does not fully meet PLOS ONE’s publication criteria as it currently stands. Therefore, we invite you to submit a revised version of the manuscript that addresses the points raised during the review process.

==============================

**ACADEMIC EDITOR:**

The manuscript needs revision. Authors may revise the manuscript with respect to the reviewer comments.

Please submit your revised manuscript by Dec 21 2025 11:59PM. If you will need more time than this to complete your revisions, please reply to this message or contact the journal office at plosone@plos.org. When you're ready to submit your revision, log on to https://www.editorialmanager.com/pone/ and select the 'Submissions Needing Revision' folder to locate your manuscript file.

Please include the following items when submitting your revised manuscript:

- A rebuttal letter that responds to each point raised by the academic editor and reviewer(s). You should upload this letter as a separate file labeled 'Response to Reviewers'.
- A marked-up copy of your manuscript that highlights changes made to the original version. You should upload this as a separate file labeled 'Revised Manuscript with Track Changes'.
- An unmarked version of your revised paper without tracked changes. You should upload this as a separate file labeled 'Manuscript'.

If you would like to make changes to your financial disclosure, please include your updated statement in your cover letter. Guidelines for resubmitting your figure files are available below the reviewer comments at the end of this letter.

If applicable, we recommend that you deposit your laboratory protocols in protocols.io to enhance the reproducibility of your results. Protocols.io assigns your protocol its own identifier (DOI) so that it can be cited independently in the future. For instructions see: https://journals.plos.org/plosone/s/submission-guidelines#loc-laboratory-protocols. Additionally, PLOS ONE offers an option for publishing peer-reviewed Lab Protocol articles, which describe protocols hosted on protocols.io. Read more information on sharing protocols at https://plos.org/protocols?utm_medium=editorial-email&utm_source=authorletters&utm_campaign=protocols.

We look forward to receiving your revised manuscript.

Kind regards,

S Ezhil Vendan, Ph.D

Academic Editor

PLOS ONE

Journal Requirements:

When submitting your revision, we need you to address these additional requirements.

1. Please ensure that your manuscript meets PLOS ONE's style requirements, including those for file naming. The PLOS ONE style templates can be found at

https://journals.plos.org/plosone/s/file?id=wjVg/PLOSOne_formatting_sample_main_body.pdf and

https://journals.plos.org/plosone/s/file?id=ba62/PLOSOne_formatting_sample_title_authors_affiliations.pdf

2. Please include a complete copy of PLOS’ questionnaire on inclusivity in global research in your revised manuscript. Our policy for research in this area aims to improve transparency in the reporting of research performed outside of researchers’ own country or community. The policy applies to researchers who have travelled to a different country to conduct research, research with Indigenous populations or their lands, and research on cultural artefacts. The questionnaire can also be requested at the journal’s discretion for any other submissions, even if these conditions are not met.  Please find more information on the policy and a link to download a blank copy of the questionnaire here: https://journals.plos.org/plosone/s/best-practices-in-research-reporting. Please upload a completed version of your questionnaire as Supporting Information when you resubmit your manuscript.

3. Thank you for stating the following financial disclosure:

“This research was supported by the Main Research Program (E0232100-03) of the Korea Food Research Institute (KFRI) funded by the Ministry of Science and ICT.”

Please state what role the funders took in the study. If the funders had no role, please state: "The funders had no role in study design, data collection and analysis, decision to publish, or preparation of the manuscript."

If this statement is not correct you must amend it as needed.

Please include this amended Role of Funder statement in your cover letter; we will change the online submission form on your behalf.

4. Please provide a complete Data Availability Statement in the submission form, ensuring you include all necessary access information or a reason for why you are unable to make your data freely accessible. If your research concerns only data provided within your submission, please write "All data are in the manuscript and/or supporting information files" as your Data Availability Statement.

5. Thank you for stating the following in the Acknowledgments Section of your manuscript:

“This research was supported by the Main Research Program (E0232100-03) of the Korea Food Research Institute (KFRI) funded by the Ministry of Science and ICT.”

We note that you have provided funding information that is not currently declared in your Funding Statement. However, funding information should not appear in the Acknowledgments section or other areas of your manuscript. We will only publish funding information present in the Funding Statement section of the online submission form.

Please remove any funding-related text from the manuscript and let us know how you would like to update your Funding Statement. Currently, your Funding Statement reads as follows:

“This research was supported by the Main Research Program (E0232100-03) of the Korea Food Research Institute (KFRI) funded by the Ministry of Science and ICT.”

Please include your amended statements within your cover letter; we will change the online submission form on your behalf.

6. We note that you have included the phrase “data not shown” in your manuscript. Unfortunately, this does not meet our data sharing requirements. PLOS does not permit references to inaccessible data. We require that authors provide all relevant data within the paper, Supporting Information files, or in an acceptable, public repository. Please add a citation to support this phrase or upload the data that corresponds with these findings to a stable repository (such as Figshare or Dryad) and provide and URLs, DOIs, or accession numbers that may be used to access these data. Or, if the data are not a core part of the research being presented in your study, we ask that you remove the phrase that refers to these data.

7. If the reviewer comments include a recommendation to cite specific previously published works, please review and evaluate these publications to determine whether they are relevant and should be cited. There is no requirement to cite these works unless the editor has indicated otherwise.

**Additional Editor Comments:**

The manuscript needs revision with respect to the reviewers comments.

[Note: HTML markup is below. Please do not edit.]

Reviewers' comments:

Reviewer's Responses to Questions

**Comments to the Author**

1. Is the manuscript technically sound, and do the data support the conclusions?

The manuscript must describe a technically sound piece of scientific research with data that supports the conclusions. Experiments must have been conducted rigorously, with appropriate controls, replication, and sample sizes. The conclusions must be drawn appropriately based on the data presented.

Reviewer #1: Partly

Reviewer #2: Partly

**********

2. Has the statistical analysis been performed appropriately and rigorously?

Reviewer #1: Yes

Reviewer #2: No

**********

3. Have the authors made all data underlying the findings in their manuscript fully available?

The PLOS Data policy requires authors to make all data underlying the findings described in their manuscript fully available without restriction, with rare exception (please refer to the Data Availability Statement in the manuscript PDF file). The data should be provided as part of the manuscript or its supporting information, or deposited to a public repository. For example, in addition to summary statistics, the data points behind means, medians and variance measures should be available. If there are restrictions on publicly sharing data—e.g. participant privacy or use of data from a third party—those must be specified.

Reviewer #1: No

Reviewer #2: No

**********

4. Is the manuscript presented in an intelligible fashion and written in standard English?

PLOS ONE does not copyedit accepted manuscripts, so the language in submitted articles must be clear, correct, and unambiguous. Any typographical or grammatical errors should be corrected at revision, so please note any specific errors here.

Reviewer #1: Yes

Reviewer #2: Yes

**********

5. Review Comments to the Author

Please use the space provided to explain your answers to the questions above. You may also include additional comments for the author, including concerns about dual publication, research ethics, or publication ethics. (Please upload your review as an attachment if it exceeds 20,000 characters)

Reviewer #1: This study explores the application of microplasma-treated water for removing organophosphorus pesticides (diazinon and chlorpyrifos) from perilla leaves and broccoli surfaces, demonstrating technical novelty compared to chlorinated water treatment. The topic addresses practical needs in agricultural product safety and provides valuable insights for pesticide residue removal technologies. While the research is well-motivated, several aspects require clarification and improvement to strengthen the manuscript.

1、Section 2.1 should provide a detailed description of the sample treatment process. For instance, Line 79 mentions treating the produce surface with 2000 ppm pesticide solution, but the method of application (e.g., immersion, droplet coating) and the sample mass used are unclear. Please clarify these experimental details.

2、Section 2.5 only describes the analytical method for diazinon. As chlorpyrifos is also investigated, its testing conditions must be provided. Additionally, the method for calculating degradation efficiency should be explicitly stated.

3、The pretreatment process involves 15-minute ultrasonication. Did the authors consider whether residual active substances on the surface might continue degrading pesticides during ultrasonication? Parameters such as ultrasonic power and temperature should be specified.

4、The use of 2000 ppm pesticide concentration significantly exceeds maximum residue limits (MRLs) and realistic field residue levels (typically ppm to ppb range). Validation data near actual residue levels should be included. Moreover, the actual residual concentration after sample treatment is not provided.

5、Fig. 2A lacks error bars. Although the microplasma treatment time was 8 minutes, Figs. 2B and 2C do not show data for the full duration. Please provide complete time-course data.

6、Fig. 6 (HPLC chromatograms) should clearly label the peak positions corresponding to the detected pesticides.

7、In Section 2.5, the authors claim that all contaminated samples were treated for 4 minutes using different methods, but the specific procedures for these four treatments are not described. Please elaborate.

8、References should be formatted uniformly. For example, Reference 11 requires verification, and all references with DOI numbers should include their corresponding DOI links.

Reviewer #2: Dear Authors,

The topic of your manuscript — the use of microplasma-treated water (MPW) for the removal of pesticide residues from fresh produce — is relevant and aligns with current interests in sustainable and chemical-free decontamination methods. The concept has potential scientific and practical significance, and the experimental approach demonstrates effort in exploring alternative postharvest treatments.

However, after a detailed evaluation, it appears that the manuscript requires substantial revision to meet scientific and methodological standards expected for publication in PLOS ONE. The study design lacks sufficient analytical validation, several methodological descriptions are incomplete or imprecise, and the data interpretation often extends beyond the evidence presented.

Specific and detailed comments are provided below to help improve the rigor, transparency, and reproducibility of your work. Addressing these issues thoroughly will be essential before the study can be reconsidered for publication.

Kind regards,

Reviewer

Introduction

General comments:

The introduction presents a potentially relevant topic but does not convincingly justify the research gap or demonstrate the novelty of applying microplasma-treated water (MPW) for pesticide removal. A concise, technically precise revision should focus on: (i) the real and documented issue of pesticide residues on fresh produce, (ii) the limitations of current decontamination techniques, and (iii) the specific advantages and innovative aspects of MPW.

The description of organophosphorus pesticides (OPPs) as “highly water-soluble” and “substituting organochlorines” is oversimplified and partially inaccurate. OPPs are moderately polar compounds, and their substitution for organochlorines was primarily driven by lower environmental persistence rather than higher solubility. This section should be revised using accurate chemical reasoning and supported by appropriate scientific references.

Line 31 – Why are U.S. statistics cited when the research was conducted in Korea? Data relevant to the study region should be presented.

Line 37 – The description of pesticide use and its impact on human health should be supported by more than two references to ensure sufficient scientific grounding.

Line 42 – Avoid the statement that “pesticides are beneficial to agronomy.” Pesticides are necessary for maintaining yield stability but not inherently beneficial. Such phrasing is simplistic and repetitive. The introduction should be condensed and focused on identifying the scientific gap rather than reiterating general background information.

Lines 46–55 - The discussion of cleaning and decontamination methods is unbalanced and contains inaccuracies. “Baking” and “freeze-drying” are not recognized or practical approaches for pesticide removal from fresh produce. The claim that “UV cleaning requires additional chemicals” is incorrect — UV treatments are non-chemical and based on photolytic degradation. The authors should reference peer-reviewed studies comparing ozone, plasma, and ultrasound methods in terms of degradation efficiency, safety, and limitations.

Line 50 – More than four references are needed to substantiate claims regarding UV, ultrasound, ozone, and freeze-drying methods. The current text presents these methods as ineffective compared to MPW, although scientific evidence does not necessarily support such contrast.

Line 66-67: A clear explanation of MPW’s mode of action is necessary. The reader should understand what differentiates MPW from other plasma-based treatments (e.g., lower energy requirements, generation of reactive species in the aqueous phase, or better scalability).

Materials and methods

General comments:

The reported pesticide concentration of 2000 ppm is unrealistically high compared with residue levels typically found on fresh produce. The authors should provide a clear justification for this choice and discuss how such an elevated concentration affects the environmental and practical relevance of their findings.

The application of 2 mL of pesticide solution per sample lacks standardization relative to the produce surface area. For reproducibility, the authors should specify the size and surface area of each sample, describe the application method (spraying, pipetting, immersion), and confirm that deposition was consistent among replicates.

The statement “pesticides were air dried for 2 h” is too vague. Drying conditions such as temperature, humidity, and airflow should be reported, as these parameters significantly influence pesticide adsorption and volatilization.

The expression “at least three separate treatments were done” is ambiguous. The authors should clarify whether this refers to biological replicates (independent samples) or technical replicates (repeated measurements of the same extract).

HPLC is an analytical technique, not an extraction method. The solvent extraction procedure should be described separately, including solvent type, volume, extraction time, agitation, and cleanup steps, followed by a clear explanation that pesticide quantification was performed by HPLC.

While the preparation of a 2000 ppm pesticide stock solution in methanol is mentioned, essential details such as the purity of pesticide standards, storage conditions, and calibration curve preparation are missing. These are necessary for method reproducibility and analytical validation.

Terms like “function producer” and “current stimulator” are non-standard and should be replaced with “function generator” and “power amplifier.” Likewise, phrases such as “elevated voltage swapping converter” are unclear and should be revised using proper electrical engineering terminology.

Although the electrical configuration is described in detail, there is no linkage between operational parameters and the physicochemical characteristics of the treated water (e.g., concentration of reactive oxygen/nitrogen species, pH, oxidation–reduction potential). Quantitative data should be presented to substantiate the claimed treatment intensity.

The description of the optical emission spectroscopy (OES) setup is overly general and lacks critical parameters required for reproducibility. Essential information such as spectral range (nm), integration time, spectral resolution (Δλ), number of averaged scans, and identification method for emission lines (e.g., OH, NO, N₂, O) should be included. The manuscript mentions that spectra were recorded, but no representative OES spectra or emission peak identifications are provided. Without these data, claims regarding reactive species generation remain unsubstantiated.

Misuse of terminology should be corrected throughout (e.g., “inoculated” should not be used to describe pesticide application).

Line 76 - The term “pesticide inoculation” is not appropriate in this context. The word “inoculation” is typically reserved for introducing microorganisms or biological material (e.g., bacterial inoculation, viral inoculation). Since the experiment involved the deliberate application of pesticide solutions onto plant surfaces, a more accurate expression would be “pesticide application”,

line 68–70 - The authors cite a previous study reporting 97–99% degradation within 1–3 minutes. If such high efficiency was already achieved, the novelty of the current work must be clearly defined — what new question or improvement does this study address? Otherwise, the manuscript risks being perceived as confirmatory rather than innovative.

Line 87 - The description of the microplasma system is highly dependent on prior publications and therefore not self-contained or reproducible. All critical experimental parameters sholud be reported within the manuscript itself, not by reference only.

Line 88- The authors state that an “improved microplasma generator” was used, yet no technical improvements are actually described. It remains unclear what was modified relative to the previous design (geometry, power supply, discharge frequency, or cooling system). The term “improved” should be replaced with a quantitative or descriptive explanation of specific design changes.

Line 94 - Several values are confusing or imprecise. For example, “the working energy was about 30 kV” should refer to voltage, not energy. Units such as “W/h” for power consumption are incorrect; power should be expressed in watts (W), not watt-hours per hour. Please review and correct all physical quantities and units.

Line 112 - Reference [26] is cited for “further evidence,” - complete OES method should be described within the paper. A reader should not have to consult earlier work to reproduce the spectral measurements.

Line 113 – Section determination acks the quantitative and procedural detail necessary to validate the reported radical concentrations. Authors must include calibration information, replicate numbers, mean ± SD values, and a clear explanation of how each reactive species contributes to the oxidative capacity of MPW.

Line 135- 142 - The extraction and HPLC quantification steps are described, but no method validation data are presented. Recovery rate, linearity, limits of detection (LOD) and quantification (LOQ), and repeatability are all essential for assessing analytical reliability. Without these, the reported degradation efficiencies are not scientifically defensible. Using 20 mL of methanol for extraction without specifying sample mass or surface area prevents any quantitative comparison between treatments. Extraction efficiency depends heavily on sample-to-solvent ratio, which must be stated.

Although the instrument and column are named, critical chromatographic parameters are missing — retention time, run time, chromatogram validation, calibration curve details, and use of standards. No information is provided on how chlorpyrifos was analyzed (different wavelength or mobile phase?). The so-called “negative control” (device switched off for 4 min) does not represent a valid procedural blank. Proper controls should include (i) untreated samples spiked with pesticide but not exposed to MPW, and (ii) solvent-only blanks to account for potential losses during extraction and handling.

The section does not specify number of replicates, variability (SD), or statistical tests applied to analytical results.

The methods describe diazinon analysis only; chlorpyrifos is mentioned in the introduction but lacks any chromatographic or detection details here. The protocol must be expanded to include both pesticides explicitly.

Line 145-155 - The description of color measurement lacks essential methodological details such as measurement geometry, lighting conditions, and sampling procedure. Terminology is incorrect (the device is a spectrophotometer, not a “camera”. Biological replication and statistical analysis of color changes are also missing.

Line 162 – Why did authors choose the Mann–Whitney U test for multi-factorial experiments involving multiple treatments and variables? ANOVA with post hoc testing would be more suitable.

Results

Results section is descriptive rather than analytical, lacking quantitative rigor, validation, and appropriate statistical evidence to substantiate the conclusions.

Findings are merely restated from previous work, and no actual OES spectra or peak identification are shown; thus, claims about reactive species formation remain unsupported within this manuscript.

Results show apparent high degradation, but analytical validation (recovery, LOD/LOQ, calibration) and replicate variation are missing, making the numerical efficiencies unreliable; also, chromatograms and statistical significance are not shown.

Interpretations about ozone and NOx behavior are speculative since no direct chemical measurements are provided

Disscussion

The discussion restates much of the results and previously published data without offering deeper analysis or critical interpretation. The mechanistic explanations for pesticide degradation (roles of NOx, O₃, and OH) are largely speculative, as no direct experimental confirmation was presented in this study. Comparisons with other plasma systems are descriptive and lack quantitative benchmarking or statistical context. Claims about “no significant chemical changes” and “non-toxicity of degradation products” are unsupported by analytical evidence, since no identification or toxicity testing of byproducts was performed. The discussion also mixes methodological details (e.g., drying conditions, moisture content) that belong to the Materials and Methods section. Overall, this section should focus on data interpretation supported by the study’s own findings, include mechanistic reasoning consistent with measured parameters, and acknowledge limitations such as unrealistic pesticide concentrations and lack of residue confirmation in treated water.

**********

6. PLOS authors have the option to publish the peer review history of their article (what does this mean?). If published, this will include your full peer review and any attached files.

If you choose “no”, your identity will remain anonymous but your review may still be made public.

**Do you want your identity to be public for this peer review?**  For information about this choice, including consent withdrawal, please see our Privacy Policy.

Reviewer #1: No

Reviewer #2: No

**********

[NOTE: If reviewer comments were submitted as an attachment file, they will be attached to this email and accessible via the submission site. Please log into your account, locate the manuscript record, and check for the action link "View Attachments". If this link does not appear, there are no attachment files.]

To ensure your figures meet our technical requirements, please review our figure guidelines: https://journals.plos.org/plosone/s/figures

You may also use PLOS’s free figure tool, NAAS, to help you prepare publication quality figures: https://journals.plos.org/plosone/s/figures#loc-tools-for-figure-preparation.

NAAS will assess whether your figures meet our technical requirements by comparing each figure against our figure specifications.

# Author comment: Author response to Decision Letter 1

**DOI:** 10.1371/journal.pone.0351955.r002

30 Jan 2026

We sincerely thank the reviewer for their careful evaluation of our manuscript and for the constructive comments and suggestions. These insights were extremely helpful and have significantly improved the clarity, quality, and scientific rigor of the manuscript. All comments have been carefully considered and addressed in detail below.

5. Review Comments to the Author

Please use the space provided to explain your answers to the questions above. You may also include additional comments for the author, including concerns about dual publication, research ethics, or publication ethics. (Please upload your review as an attachment if it exceeds 20,000 characters)

Reviewer #1: This study explores the application of microplasma-treated water for removing organophosphorus pesticides (diazinon and chlorpyrifos) from perilla leaves and broccoli surfaces, demonstrating technical novelty compared to chlorinated water treatment. The topic addresses practical needs in agricultural product safety and provides valuable insights for pesticide residue removal technologies. While the research is well-motivated, several aspects require clarification and improvement to strengthen the manuscript.

1. Section 2.1 should provide a detailed description of the sample treatment process. For instance, Line 79 mentions treating the produce surface with 2000 ppm pesticide solution, but the method of application (e.g., immersion, droplet coating) and the sample mass used are unclear. Please clarify these experimental details.

Response: We have clarified the detailed description of the sample treatment process in section “Pesticide contamination in produce samples”. Can be found in line 94 - 103.

2. Section 2.5 only describes the analytical method for diazinon. As chlorpyrifos is also investigated, its testing conditions must be provided. Additionally, the method for calculating degradation efficiency should be explicitly stated.

Reply: Chlorpyrifos detail is included in line 199 - 206. The method for calculating degradation efficiency also shown in the revised draft (supplementary information Text 6).

3. The pretreatment process involves 15-minute ultrasonication. Did the authors consider whether residual active substances on the surface might continue degrading pesticides during ultrasonication? Parameters such as ultrasonic power and temperature should be specified.

Response: We thank the reviewer for the comment. However, there appears to be a misunderstanding regarding the experimental procedure. The 15-minute ultrasonication step was not used as a pretreatment, but was applied after the treatment solely to facilitate the detachment and homogenization of surface residues for analytical extraction. Therefore, no residual active species were present during ultrasonication that could contribute to additional pesticide degradation. We have now added the missing operational parameters. The ultrasonication was performed using a bath sonicator at 40 kHz frequency, 150 W power, and a controlled water temperature of 25 ± 1°C. These details have been included in the Materials and Methods section to improve procedural transparency (Lines 171–174).

4. The use of 2000 ppm pesticide concentration significantly exceeds maximum residue limits (MRLs) and realistic field residue levels (typically ppm to ppb range). Validation data near actual residue levels should be included. Moreover, the actual residual concentration after sample treatment is not provided.

Response: We agree that 2000 ppm exceeds typical residue levels found on fresh produce. This concentration was intentionally selected for two specific reasons, which we have now clarified in the revised manuscript:

1. Methodological Standardization: High spiking levels are commonly used in degradation studies to ensure accurate quantification, minimize analytical variability at early time points, and enable clear monitoring of degradation kinetics across treatments.

2. Stress-Testing the System: The elevated concentration allows evaluation of the treatment’s capacity under worst-case or extreme contamination scenarios, which provides insight into the robustness and upper performance limits of the technology.

To address the reviewer’s concern about practical relevance, we have added a statement in the Discussion acknowledging that typical field residue levels are much lower and that real-world performance is expected to be similar or higher due to the lower pesticide burden. We also clarified that the purpose of the 2000 ppm condition is not to model actual consumer exposure but to provide a controlled, conservative test of degradation efficiency. These explanations have been incorporated into the revised Introduction (justification of experimental design) and Discussion (practical applicability). Introduction part: Line 85 - 88; Discussion Part: Line 395 – 400.

5. Fig. 2A lacks error bars. Although the microplasma treatment time was 8 minutes, Figs. 2B and 2C do not show data for the full duration. Please provide complete time-course data.

Response: The ozone concentration remained essentially constant during the 8-minute treatment, with minimal fluctuation. Given the high precision of the measurement device, error bars were not included, as they would not meaningfully reflect variation. For transparency, we have clarified this point in the figure legend.

Additional Note on Figs. 2B and 2C: Data at longer treatment times are not shown because no significant changes occurred after the reactive species reached their maximum concentration. This approach is now clarified in the figure legend.

6. Fig. 6 (HPLC chromatograms) should clearly label the peak positions corresponding to the detected pesticides.

Response: The HPLC chromatograms in Fig 6 were included primarily to illustrate overall pesticide degradation patterns rather than for quantitative peak identification. Detailed peak assignments and quantitative analysis have already been reported in the Methods section and validated through calibration and recovery experiments. However, in the revised manuscript peak position were labeled clearly in the figure.

7. In Section 2.5, the authors claim that all contaminated samples were treated for 4 minutes using different methods, but the specific procedures for these four treatments are not described. Please elaborate.

Response: Among the four methods tested, only plasma treatment resulted in a significant reduction of pesticides; the other methods showed negligible effects. For this reason, detailed procedures for the non-effective methods were not originally described. Nevertheless, in response to this comment, we have briefly summarized the treatment conditions for all four methods in the revised manuscript (Different modes of produce washing section) to improve clarity (lines 154–166).

8. References should be formatted uniformly. For example, Reference 11 requires verification, and all references with DOI numbers should include their corresponding DOI links.

Reply: DOI links have been included for all references where available to ensure completeness and traceability.

Reviewer #2: Dear Authors,

The topic of your manuscript — the use of microplasma-treated water (MPW) for the removal of pesticide residues from fresh produce — is relevant and aligns with current interests in sustainable and chemical-free decontamination methods. The concept has potential scientific and practical significance, and the experimental approach demonstrates effort in exploring alternative postharvest treatments.

However, after a detailed evaluation, it appears that the manuscript requires substantial revision to meet scientific and methodological standards expected for publication in PLOS ONE. The study design lacks sufficient analytical validation, several methodological descriptions are incomplete or imprecise, and the data interpretation often extends beyond the evidence presented.

Specific and detailed comments are provided below to help improve the rigor, transparency, and reproducibility of your work. Addressing these issues thoroughly will be essential before the study can be reconsidered for publication.

Kind regards,

Reviewer

Introduction

General comments:

The introduction presents a potentially relevant topic but does not convincingly justify the research gap or demonstrate the novelty of applying microplasma-treated water (MPW) for pesticide removal. A concise, technically precise revision should focus on: (i) the real and documented issue of pesticide residues on fresh produce, (ii) the limitations of current decontamination techniques, and (iii) the specific advantages and innovative aspects of MPW.

Response: We appreciate the reviewer’s emphasis on strengthening the scientific rationale and novelty in the introduction. In response, we have substantially revised this section to address the three key areas highlighted:

(i) Documented issue of pesticide residues:

We now provide a concise summary of the prevalence of pesticide residues on fresh produce, supported by recent global monitoring reports (e.g., EFSA, FDA, FAO) and peer-reviewed studies demonstrating potential health risks and regulatory concern.

Inclusion: Can be found in line 37 to 43.

New references:

8. European Food Safety Authority (EFSA). 2023. The 2021 European Union report on pesticide residues in food. EFSA Journal. 21:e08033. https://doi.org/10.2903/j.efsa.2023.8033

9. U.S. Food and Drug Administration (FDA). 2023. Pesticide Residue Monitoring Program Fiscal Year 2021 Annual Summary. Silver Spring (MD): U.S. Food and Drug Administration.

10. Kim KH, Kabir E, Jahan SA. 2017. Exposure to pesticides and the associated human health effects. Sci Total Environ. 575:525–535. https://doi.org/10.1016/j.scitotenv.2016.09.009

(ii) Limitations of current decontamination techniques:

The revised introduction now discusses the shortcomings of conventional washing methods (e.g., tap water, chlorinated water, ozone, organic acids), including limited removal efficiency, chemical residues, cost, or safety concerns. These limitations clearly support the need for alternative, safe, and effective decontamination strategies. Line 54 - 62 revised substantially.

(iii) Advantages and novelty of MPW:

We have clarified the innovative aspects of microplasma-treated water, including its capability to generate reactive oxygen and nitrogen species (RONS), its antimicrobial and oxidative potential, and its emerging application in food decontamination. These additions significantly improve the scientific justification for the study and clarify the novelty of applying MPW for pesticide degradation. We thank the reviewer for pointing out this gap, which has helped strengthen the clarity and impact of the manuscript. Detail can be found in line 74 – 85.

The description of organophosphorus pesticides (OPPs) as “highly water-soluble” and “substituting organochlorines” is oversimplified and partially inaccurate. OPPs are moderately polar compounds, and their substitution for organochlorines was primarily driven by lower environmental persistence rather than higher solubility. This section should be revised using accurate chemical reasoning and supported by appropriate scientific references.

Response: We agree that our original wording was oversimplified. The replacement of organochlorine pesticides (OCPs) by organophosphorus pesticides (OPPs) was primarily due to their much lower environmental persistence and lower bioaccumulation potential, rather than higher water solubility. We have revised the manuscript to reflect that OPPs are generally moderately polar compounds with low-to-moderate water solubility, depending on the specific molecule. Original manuscript is revised can be found in line 46 - 48.

Line 31 – Why are U.S. statistics cited when the research was conducted in Korea? Data relevant to the study region should be presented.

Response: We agree that regional data are more appropriate for supporting the context of this study. Accordingly, we have removed the U.S. statistics and replaced them with Korea-specific data reflecting fresh produce consumption trends. These Korea-specific data have now been incorporated into the revised introduction, ensuring better alignment with the study’s geographical context. We appreciate the reviewer’s suggestion, which has strengthened the regional relevance of our manuscript. Line 28 - 34.

New References:

1. Ministry of Health and Welfare (MOHW), Korea Disease Control and Prevention Agency (KDCA). 2022. Korea National Health and Nutrition Examination Survey (KNHANES) VIII-4, 2022. Sejong, Republic of Korea.

2. Ministry of Agriculture, Food and Rural Affairs (MAFRA). 2023. Food Consumption Trends and Agricultural Outlook in Korea. Sejong, Republic of Korea.

3. Lee JS, Kim J. 2021. Trends in fruit and vegetable consumption and dietary quality among Korean adults. Nutr Res Pract. 15:641–652. https://doi.org/10.4162/nrp.2021.15.5.641

Line 37 – The description of pesticide use and its impact on human health should be supported by more than two references to ensure sufficient scientific grounding.

Response: In the revised manuscript, we have expanded the discussion on pesticide use and associated human health impacts and have incorporated additional peer-reviewed references to strengthen the scientific grounding. These include recent global assessments and epidemiological studies demonstrating links between pesticide exposure and adverse health outcomes such as endocrine disruption, neurotoxicity, and chronic disease risks. The section has now been updated to provide a more comprehensive and well-supported overview in alignment with the reviewer’s suggestion.

Additional References Included in the Revision:

11. Mostafalou, S., & Abdollahi, M. (2017). Pesticides and human chronic diseases: Evidences, mechanisms, and perspectives. Toxicology and Applied Pharmacology, 329, 45–55.

12. Kim, K. H., Kabir, E., & Jahan, S. A. (2017). Exposure to pesticides and the associated human health effects. Science of the Total Environment, 575, 525–535.

13. Mnif, W., et al. (2011). Effect of endocrine-disrupting pesticides: A review. International Journal of Environmental Research and Public Health, 8(6), 2265–2303.

Line 42 – Avoid the statement that “pesticides are beneficial to agronomy.” Pesticides are necessary for maintaining yield stability but not inherently beneficial. Such phrasing is simplistic and repetitive. The introduction should be condensed and focused on identifying the scientific gap rather than reiterating general background information.

Response: We agree that the original phrasing was overly simplistic and did not accurately reflect the role of pesticides in agricultural systems. In response, we have removed the statement that “pesticides are beneficial to agronomy” and revised the text to provide a more precise explanation. The updated sentence now emphasizes the necessity of pesticides for maintaining yield stability rather than portraying them as inherently beneficial. We have also condensed surrounding content to improve clarity and to keep the introduction focused on defining the scientific gap. Line 49 - 51

Lines 46–55 - The discussion of cleaning and decontamination methods is unbalanced and contains inaccuracies. “Baking” and “freeze-drying” are not recognized or practical approaches for pesticide removal from fresh produce. The claim that “UV cleaning requires additional chemicals” is incorrect — UV treatments are non-chemical and based on photolytic degradation. The authors should reference peer-reviewed studies comparing ozone, plasma, and ultrasound methods in terms of degradation efficiency, safety, and limitations.

Response: Thank you for this helpful comment. We have substantially revised this section to improve clarity and focus, and the content has now been reorganized to better reflect the relevance of the food washing system. The revisions ensure that the discussion is clearly aligned with its applicability and significance to the topic. The revised paragraph can be checked in line 54-62.

Line 50 – More than four references are needed to substantiate claims regarding UV, ultrasound, ozone, and freeze-drying methods. The current text presents these m

Attachment Submitted filename: Response to Reviewer.docx

# Peer review: Decision Letter 1

**DOI:** 10.1371/journal.pone.0351955.r003

**Authors:** S Ezhil Vendan, S Ezhil Vendan

19 Mar 2026

PONE-D-25-44051R1

Degradation efficiency of pesticide residues on contaminated produce surface using microplasma treated water

PLOS One

Dear Dr. Muhammad Saiful Islam Khan,

Thank you for submitting your manuscript to PLOS ONE. After careful consideration, we feel that it has merit but does not fully meet PLOS ONE’s publication criteria as it currently stands. Therefore, we invite you to submit a revised version of the manuscript that addresses the points raised during the review process.

==============================

**ACADEMIC EDITOR:**

The manuscript needs major revision with respect to the reviewers comments and following comments. Authors may revise and submit the revised manuscript with authors response to each comment.

==============================

Please submit your revised manuscript by May 03 2026 11:59PM. If you will need more time than this to complete your revisions, please reply to this message or contact the journal office at plosone@plos.org. When you're ready to submit your revision, log on to https://www.editorialmanager.com/pone/ and select the 'Submissions Needing Revision' folder to locate your manuscript file.

Please include the following items when submitting your revised manuscript:

- A letter that responds to each point raised by the academic editor and reviewer(s). You should upload this letter as a separate file labeled 'Response to Reviewers'.
- A marked-up copy of your manuscript that highlights changes made to the original version. You should upload this as a separate file labeled 'Revised Manuscript with Track Changes'.
- An unmarked version of your revised paper without tracked changes. You should upload this as a separate file labeled 'Manuscript'.

If you would like to make changes to your financial disclosure, please include your updated statement in your cover letter. Guidelines for resubmitting your figure files are available below the reviewer comments at the end of this letter.

If applicable, we recommend that you deposit your laboratory protocols in protocols.io to enhance the reproducibility of your results. Protocols.io assigns your protocol its own identifier (DOI) so that it can be cited independently in the future. For instructions see: https://journals.plos.org/plosone/s/submission-guidelines#loc-laboratory-protocols. Additionally, PLOS ONE offers an option for publishing peer-reviewed Lab Protocol articles, which describe protocols hosted on protocols.io. Read more information on sharing protocols at https://plos.org/protocols?utm_medium=editorial-email&utm_source=authorletters&utm_campaign=protocols.

We look forward to receiving your revised manuscript.

Kind regards,

S Ezhil Vendan, Ph.D

Academic Editor

PLOS One

Journal Requirements:

If the reviewer comments include a recommendation to cite specific previously published works, please review and evaluate these publications to determine whether they are relevant and should be cited. There is no requirement to cite these works unless the editor has indicated otherwise.

Additional Editor Comments:

The manuscript needs major revision with respect to the reviewers comments and following comments. Authors may revise and submit the revised manuscript with authors response to each comment.

The manuscript study focused pesticide degradation analysis on the spiked samples not contaminated samples. So, please avoid “contaminated produce surface” and may write specific term throughout the manuscript including manuscript title. Or, justify with relevant literature.

With respect to the study objective (Line 72) and obtained results, lacking of methodology about the chlorine washing treatment on the pesticide spiked samples. Please check and add methodology in the materials and method section.

Lacking of statistical representation in all the presented results data. Please check and add statistical significance representation in the data.

Line 32: Which standard agency or regulatory body guidelines?. Specify.

Line 68-69: With reference to the cited reference Hernandez-Borges et al., (2009), please check the sentence “We previously showed that one ……………………..” and revise with correct reference.

Line 77: Add scientific name of perilla leaf and broccoli.

Line 77-78: How the pesticide free produce were confirmed/ authenticated?.

Line 79: How much quantity of samples were treated?. Specify.

Line 80-81: Move the sentence “Pesticide samples were obtained from Sigma-Aldrich, South Korea” to before the sentence “The surfaces of the produce ………………”.

Line 80: Add purity and sample code of pesticide standards (diazinon and chlorpyrifos) of Sigma-Aldrich.

Line 82: Why the higher concentration “2 ml of 2000 ppm pesticide solution” was used in the study?. Justify with relevant literature.

Line 84: Why “At least”?. Please specify.

Line 129: Why residual analysis performed for diazinon only not for another pesticide?. Please check the sub-heading and explain in the methodology.

Line 146: In which samples, colour changes analysis were performed?. Specify

Line 368-370: Please remove the sentences “To the best of our knowledge, ………………………… treated produce”, not required.

Line 370: Avoid “our” and may write “In the present study, microplasma discharge water system performed ……………………….”.

[Note: HTML markup is below. Please do not edit.]

Reviewers' comments:

Reviewer's Responses to Questions

**Comments to the Author**

1. If the authors have adequately addressed your comments raised in a previous round of review and you feel that this manuscript is now acceptable for publication, you may indicate that here to bypass the “Comments to the Author” section, enter your conflict of interest statement in the “Confidential to Editor” section, and submit your "Accept" recommendation.

Reviewer #1: (No Response)

Reviewer #2: (No Response)

**********

2. Is the manuscript technically sound, and do the data support the conclusions?

The manuscript must describe a technically sound piece of scientific research with data that supports the conclusions. Experiments must have been conducted rigorously, with appropriate controls, replication, and sample sizes. The conclusions must be drawn appropriately based on the data presented.

Reviewer #1: Partly

Reviewer #2: Partly

**********

3. Has the statistical analysis been performed appropriately and rigorously?

Reviewer #1: Yes

Reviewer #2: No

**********

4. Have the authors made all data underlying the findings in their manuscript fully available?

The PLOS Data policy requires authors to make all data underlying the findings described in their manuscript fully available without restriction, with rare exception (please refer to the Data Availability Statement in the manuscript PDF file). The data should be provided as part of the manuscript or its supporting information, or deposited to a public repository. For example, in addition to summary statistics, the data points behind means, medians and variance measures should be available. If there are restrictions on publicly sharing data—e.g. participant privacy or use of data from a third party—those must be specified.

Reviewer #1: Yes

Reviewer #2: Yes

**********

5. Is the manuscript presented in an intelligible fashion and written in standard English?

PLOS ONE does not copyedit accepted manuscripts, so the language in submitted articles must be clear, correct, and unambiguous. Any typographical or grammatical errors should be corrected at revision, so please note any specific errors here.

Reviewer #1: Yes

Reviewer #2: Yes

**********

6. Review Comments to the Author

Please use the space provided to explain your answers to the questions above. You may also include additional comments for the author, including concerns about dual publication, research ethics, or publication ethics. (Please upload your review as an attachment if it exceeds 20,000 characters)

Reviewer #1: The authors have diligently addressed most of the concerns raised in the previous review round. The manuscript has been significantly improved in terms of experimental design and data presentation. However, two critical issues require further clarification to ensure the robustness of the conclusions and the practical interpretation of the results.

1. Potential Interference from Residual Active Species During Ultrasonication

Comment:

In the revised manuscript, the authors state that reactive nitrogen species (RNS) are the primary agents responsible for pesticide degradation and that these species are long-lived in the system. This raises a valid concern: could residual RNS present on the treated produce surface continue to degrade pesticides during the subsequent ultrasonication step used for extraction? This potential ongoing degradation would conflate the effect of the microplasma-treated water wash with the effect of ultrasonication-assisted degradation, thereby overestimating the efficiency attributed solely to the plasma-treated water.

2.In lines 383-393, the authors cite previous work showing ~99% degradation of pesticides in standard solution within 3 minutes of direct microplasma treatment. They use this to support the efficacy of their process. However, a crucial analytical step is missing in the current study: the quantification of pesticide residues in the rinse water after it has been used to wash the contaminated produce.

The efficiency of pesticide degradation in a standard solution under direct plasma exposure can differ significantly from the efficiency of removal and degradation in a complex matrix like produce wash water. Pesticides may be partially transferred from the produce surface into the wash water without being fully degraded. Therefore, relying solely on prior standard solution data is insufficient to claim complete degradation in the applied context.

Reviewer #2: Dear authors,

The revised manuscript shows improved clarity and structure, and many of the comments from the first review have been addressed. The study addresses a relevant topic and the experimental approach is of potential practical interest. However, several methodological aspects would benefit from further clarification to ensure analytical transparency and reproducibility.

In particular, although additional information on analytical validation, statistical analysis, and plasma diagnostics has been provided, some key methodological details remain insufficiently summarized in the main text. The environmental relevance of the pesticide concentration used (2000 ppm) should also be discussed more explicitly.

Specific comments

1. Analytical validation of the HPLC method

Validation parameters are reported in the Supplementary Information. It would be helpful to briefly summarize the key values (recovery range, R², LOD and LOQ) in the main manuscript to facilitate evaluation of the analytical reliability.

2. Control experiments

The description of control conditions could be clarified further. In particular, please specify whether pesticide-spiked produce washed with untreated water was included as a control to distinguish degradation from simple washing effects.

3. Environmental relevance of pesticide concentrations

The use of a 2000 ppm pesticide concentration is justified by the authors; however, this value is considerably higher than typical residue levels on fresh produce. The Discussion should more clearly acknowledge how this may affect degradation kinetics and the applicability of the results to realistic scenarios.

4. Statistical analysis

Please clarify the statistical model applied, the factors included in the analysis (e.g., washing method, pesticide type, temperature), and the number of biological replicates used in each treatment.

5. Mechanistic interpretation

Additional plasma diagnostics are useful, but the mechanistic interpretation remains partly indirect. The discussion should clearly distinguish between experimentally measured parameters and interpretations based on previous studies.

**********

7. PLOS authors have the option to publish the peer review history of their article (what does this mean?). If published, this will include your full peer review and any attached files.

If you choose “no”, your identity will remain anonymous but your review may still be made public.

**Do you want your identity to be public for this peer review?**  For information about this choice, including consent withdrawal, please see our Privacy Policy.

Reviewer #1: No

Reviewer #2: No

**********

[NOTE: If reviewer comments were submitted as an attachment file, they will be attached to this email and accessible via the submission site. Please log into your account, locate the manuscript record, and check for the action link "View Attachments". If this link does not appear, there are no attachment files.]

To ensure your figures meet our technical requirements, please review our figure guidelines: https://journals.plos.org/plosone/s/figures

You may also use PLOS’s free figure tool, NAAS, to help you prepare publication quality figures: https://journals.plos.org/plosone/s/figures#loc-tools-for-figure-preparation.

NAAS will assess whether your figures meet our technical requirements by comparing each figure against our figure specifications.

# Author comment: Author response to Decision Letter 2

**DOI:** 10.1371/journal.pone.0351955.r004

28 Mar 2026

We sincerely thank the Editor and reviewers for their careful evaluation of our manuscript and for the constructive comments and suggestions. These insights were extremely helpful and have significantly improved the clarity, quality, and scientific rigor of the manuscript. All comments have been carefully considered and addressed in detail below.

Additional Editor Comments:

The manuscript needs major revision with respect to the reviewers comments and following comments. Authors may revise and submit the revised manuscript with authors response to each comment.

The manuscript study focused pesticide degradation analysis on the spiked samples not contaminated samples. So, please avoid “contaminated produce surface” and may write specific term throughout the manuscript including manuscript title. Or, justify with relevant literature.

Reply: “Pesticide-spiked” word is used in throughout the manuscript.

With respect to the study objective (Line 72) and obtained results, lacking of methodology about the chlorine washing treatment on the pesticide spiked samples. Please check and add methodology in the materials and method section.

Reply: Thank you for your valuable comment. We agree that the description of the chlorine washing treatment was insufficient in the original manuscript. Accordingly, a detailed methodology for the chlorine washing of pesticide-spiked samples has now been added to the Materials and Methods section (Lines 172–177).

Lacking of statistical representation in all the presented results data. Please check and add statistical significance representation in the data.

Reply: Added!

Line 32: Which standard agency or regulatory body guidelines? Specify.

Reply: Line is modified as KNHANES 2022 reports that over 72% of adults consume fresh vegetables regularly, which aligns with the Korean Dietary Guidelines issued by the Ministry of Health and Welfare promoting increased vegetable intake [1] which Specified the regulatory body.

Line 68-69: With reference to the cited reference Hernandez-Borges et al., (2009), please check the sentence “We previously showed that one ……………………..” and revise with correct reference.

Reply: This one will be reference 26.

Line 77: Add scientific name of perilla leaf and broccoli.

Reply: Included.

Line 77-78: How the pesticide free produce were confirmed/ authenticated?

Reply: Thank you for your insightful comment. The produce samples used in this study were procured from a government-regulated fresh produce market in Korea that certifies products as organic. We acknowledge that “organic” does not necessarily guarantee the complete absence of pesticide residues. Therefore, the manuscript has been revised to clarify that the samples were organically sourced produce rather than analytically confirmed pesticide-free samples (Line 95).

Line 79: How much quantity of samples were treated?. Specify.

Reply: Mentioned in the revised manuscript (Lines 97-98).

Line 80-81: Move the sentence “Pesticide samples were obtained from Sigma-Aldrich, South Korea” to before the sentence “The surfaces of the produce ………………”.

Reply: It is now shifted in relevant paragraph.

Line 80: Add purity and sample code of pesticide standards (diazinon and chlorpyrifos) of Sigma-Aldrich.

Reply: Added!! Line 202-203.

Line 82: Why the higher concentration “2 ml of 2000 ppm pesticide solution” was used in the study?. Justify with relevant literature.

Response: We thank the reviewer for this valuable observation. Pesticide spiking was performed by applying 2 mL of a 2000 ppm solution to each treatment batch (3 g perilla leaf; 200 g broccoli), resulting in a uniform residue level in the range of 0.1–0.5 µg/g, in accordance with SANTE/11312/2021 guidelines to ensure environmental relevance. We have also added a statement acknowledging that degradation kinetics at higher initial concentrations may differ from those observed under realistic conditions. These revisions improve the clarity and practical applicability of our findings. The corresponding changes have been incorporated in lines 87–91, lines 103 – 106 and lines 545-546.

Line 84: Why “At least”?. Please specify.

Reply: Sentence was re-written. Three independent treatments of each sample were performed using microplasma prior to HPLC analysis to ensure reproducibility and statistical reliability of the results.

Line 129: Why residual analysis performed for diazinon only not for another pesticide?. Please check the sub-heading and explain in the methodology.

Reply: Chlorpyrifos detail is included in the section Preparation of stock solutions and analytical calibration methods.

Line 146: In which samples, colour changes analysis were performed?. Specify

Reply: Specified in line 241-244.

Line 368-370: Please remove the sentences “To the best of our knowledge, ………………………… treated produce”, not required.

Reply: Removed.

Line 370: Avoid “our” and may write “In the present study, microplasma discharge water system performed ……………………….”.

Reply: Done!

6. Review Comments to the Author

Please use the space provided to explain your answers to the questions above. You may also include additional comments for the author, including concerns about dual publication, research ethics, or publication ethics. (Please upload your review as an attachment if it exceeds 20,000 characters).

Reviewer #1: The authors have diligently addressed most of the concerns raised in the previous review round. The manuscript has been significantly improved in terms of experimental design and data presentation. However, two critical issues require further clarification to ensure the robustness of the conclusions and the practical interpretation of the results.

1. Potential Interference from Residual Active Species During Ultrasonication

Comment:

In the revised manuscript, the authors state that reactive nitrogen species (RNS) are the primary agents responsible for pesticide degradation and that these species are long-lived in the system. This raises a valid concern: could residual RNS present on the treated produce surface continue to degrade pesticides during the subsequent ultrasonication step used for extraction? This potential ongoing degradation would conflate the effect of the microplasma-treated water wash with the effect of ultrasonication-assisted degradation, thereby overestimating the efficiency attributed solely to the plasma-treated water.

Response: We thank the reviewer for this insightful comment. We agree that residual reactive nitrogen species (RNS) could potentially contribute to further degradation during extraction. To address this concern, samples were immediately transferred to methanol following treatment. Methanol acts as an effective quenching and extraction solvent, rapidly dissolving pesticide residues while minimizing continued reactions from residual reactive species. Additionally, ultrasonication was conducted under controlled temperature conditions (22 ± 1°C) to avoid any secondary degradation effects. We have clarified this point in the revised manuscript (Pesticide extraction methods), emphasizing that the ultrasonication step was intended solely to enhance extraction efficiency rather than induce degradation. Line 189-185 and line 331-334.

2. In lines 383-393, the authors cite previous work showing ~99% degradation of pesticides in standard solution within 3 minutes of direct microplasma treatment. They use this to support the efficacy of their process. However, a crucial analytical step is missing in the current study: the quantification of pesticide residues in the rinse water after it has been used to wash the contaminated produce.

The efficiency of pesticide degradation in a standard solution under direct plasma exposure can differ significantly from the efficiency of removal and degradation in a complex matrix like produce wash water. Pesticides may be partially transferred from the produce surface into the wash water without being fully degraded. Therefore, relying solely on prior standard solution data is insufficient to claim complete degradation in the applied context.

Response: “While pesticide residues in rinse water were not directly measured, the 4-minute plasma treatment exceeds the 3-minute degradation time established in our previous studies for standard solutions. Therefore, we reasonably expect that any pesticides transferred to the wash water were effectively degraded. A statement clarifying this assumption has been added to the Methods and Discussion sections (Lines 180 – 186). And in the discussion part line 418 to 423.

Reviewer #2:

Dear authors, The revised manuscript shows improved clarity and structure, and many of the comments from the first review have been addressed. The study addresses a relevant topic and the experimental approach is of potential practical interest. However, several methodological aspects would benefit from further clarification to ensure analytical transparency and reproducibility.

In particular, although additional information on analytical validation, statistical analysis, and plasma diagnostics has been provided, some key methodological details remain insufficiently summarized in the main text. The environmental relevance of the pesticide concentration used (2000 ppm) should also be discussed more explicitly.

Specific comments

1. Analytical validation of the HPLC method

Validation parameters are reported in the Supplementary Information. It would be helpful to briefly summarize the key values (recovery range, R², LOD and LOQ) in the main manuscript to facilitate evaluation of the analytical reliability.

Response: “We appreciate the reviewer’s suggestion. Key validation parameters have now been summarized in the main text (Methods section). Line 214-216

2. Control experiments

The description of control conditions could be clarified further. In particular, please specify whether pesticide-spiked produce washed with untreated water was included as a control to distinguish degradation from simple washing effects.

Response: “Produce washed with untreated water was included as a control in all experiments to distinguish the effect of simple washing from plasma-assisted degradation. This has been clarified in the Methods section.” Line 108-109.

3. Environmental relevance of pesticide concentrations

The use of a 2000 ppm pesticide concentration is justified by the authors; however, this value is considerably higher than typical residue levels on fresh produce. The Discussion should more clearly acknowledge how this may affect degradation kinetics and the applicability of the results to realistic scenarios.

Response: We thank the reviewer for this valuable observation. Pesticide spiking was performed by applying 2 mL of a 2000 ppm solution to each treatment batch (3 g perilla leaf; 200 g broccoli), resulting in a uniform residue level in the range of 0.1–0.5 µg/g, in accordance with SANTE/11312/2021 guidelines to ensure environmental relevance. We have also added a statement acknowledging that degradation kinetics at higher initial concentrations may differ from those observed under realistic conditions. These revisions improve the clarity and practical applicability of our findings. The corresponding changes have been incorporated in lines 87–91, lines 103 – 106 and lines 545-546.

4. Statistical analysis

Please clarify the statistical model applied, the factors included in the analysis (e.g., washing method, pesticide type, temperature), and the number of biological replicates used in each treatment.

Response: “Statistical analysis was performed using ANOVA, with factors including washing method, pesticide type, and temperature. A minimum of three biological replicates per treatment were included, and in some cases up to five replicates. This has been clarified in the Methods section.”

5. Mechanistic interpretation

Reviewer Comment:

Additional plasma diagnostics are useful, but the mechanistic interpretation remains partly indirect. The discussion should clearly distinguish between experimentally measured parameters and interpretations based on previous studies.

Response: We sincerely thank the reviewer for this valuable comment. We agree that clearly distinguishing between experimentally measured results and literature-based interpretations is important. In fact, this distinction was already indicated in the original manuscript. For example, in Discussion Section (Lines 458– 485), we state: “Detailed methodologies and analyses used to elucidate the roles of individual reactive species are provided in Text S6 and Fig. S2.” This was intended to clarify that while reactive species (e.g., OH radicals, RNS, H₂O₂, and pH changes) were experimentally quantified in the present study, the specific roles and relative contributions of individual species in pesticide degradation are inferred based on our previous work and other published studies, as appropriately cited. However, we acknowledge that this distinction may not have been sufficiently explicit. Therefore, we have revised the relevant paragraph in the Discussion section to more clearly separate (i) findings obtained directly from the current study and (ii) mechanistic interpretations supported by prior studies.

Attachment Submitted filename: Response to Reviewer R2.docx

# Peer review: Decision Letter 2

**DOI:** 10.1371/journal.pone.0351955.r005

**Authors:** S Ezhil Vendan, S Ezhil Vendan, S Ezhil Vendan

15 Apr 2026

PONE-D-25-44051R2

Degradation Efficiency of Pesticide Residues on Pesticide-Spiked Produce Surfaces Using Microplasma-Treated Water

PLOS One

Dear Dr. Khan,

Thank you for submitting your manuscript to PLOS ONE. After careful consideration, we feel that it has merit but does not fully meet PLOS ONE’s publication criteria as it currently stands. Therefore, we invite you to submit a revised version of the manuscript that addresses the points raised during the review process.

==============================

**ACADEMIC EDITOR:**

With the reviewer comments, the revised manuscript is improved compared to previous version. Though, the manuscript needs revision with respect to the suggested comments.

==============================

Please submit your revised manuscript by May 30 2026 11:59PM. If you will need more time than this to complete your revisions, please reply to this message or contact the journal office at plosone@plos.org. When you're ready to submit your revision, log on to https://www.editorialmanager.com/pone/ and select the 'Submissions Needing Revision' folder to locate your manuscript file.

Please include the following items when submitting your revised manuscript:

- A letter that responds to each point raised by the academic editor and reviewer(s). You should upload this letter as a separate file labeled 'Response to Reviewers'.
- A marked-up copy of your manuscript that highlights changes made to the original version. You should upload this as a separate file labeled 'Revised Manuscript with Track Changes'.
- An unmarked version of your revised paper without tracked changes. You should upload this as a separate file labeled 'Manuscript'.

If you would like to make changes to your financial disclosure, please include your updated statement in your cover letter. Guidelines for resubmitting your figure files are available below the reviewer comments at the end of this letter.

If applicable, we recommend that you deposit your laboratory protocols in protocols.io to enhance the reproducibility of your results. Protocols.io assigns your protocol its own identifier (DOI) so that it can be cited independently in the future. For instructions see: https://journals.plos.org/plosone/s/submission-guidelines#loc-laboratory-protocols. Additionally, PLOS ONE offers an option for publishing peer-reviewed Lab Protocol articles, which describe protocols hosted on protocols.io. Read more information on sharing protocols at https://plos.org/protocols?utm_medium=editorial-email&utm_source=authorletters&utm_campaign=protocols.

As the corresponding author, your ORCID iD is verified in the submission system and will appear in the published article. PLOS supports the use of ORCID, and we encourage all coauthors to register for an ORCID iD and use it as well. Please encourage your coauthors to verify their ORCID iD within the submission system before final acceptance, as unverified ORCID iDs will not appear in the published article. *Only*  the individual author can complete the verification step; PLOS staff *cannot*  verify ORCID iDs on behalf of authors.

We look forward to receiving your revised manuscript.

Kind regards,

S Ezhil Vendan, Ph.D

Academic Editor

PLOS One

Journal Requirements:

If the reviewer comments include a recommendation to cite specific previously published works, please review and evaluate these publications to determine whether they are relevant and should be cited. There is no requirement to cite these works unless the editor has indicated otherwise.

Additional Editor Comments:

With the reviewer comments, the revised manuscript is improved compared to previous version. Though, the manuscript needs revision with respect to the reviewer comments and following comments.

Line 1-2: In the manuscript title, write like “……. on pesticide-spiked perilla and broccoli surfaces using ……” instead of “……… on pesticide-spiked produce surfaces using ………”.

Line 15: Write like “Pesticide-spiked food produce surfaces …..” instead of “Pesticide-spiked Produce surfaces …..”.

Line 20-21: Remove the SD values “(± 2.3–3.1%), (± 2.8–3.6%)” and “(mean ± SD, n = 3)”.

Please check the typographical and grammatical errors throughout the manuscript.

[Note: HTML markup is below. Please do not edit.]

Reviewers' comments:

Reviewer's Responses to Questions

**Comments to the Author**

1. If the authors have adequately addressed your comments raised in a previous round of review and you feel that this manuscript is now acceptable for publication, you may indicate that here to bypass the “Comments to the Author” section, enter your conflict of interest statement in the “Confidential to Editor” section, and submit your "Accept" recommendation.

Reviewer #1: All comments have been addressed

Reviewer #2: All comments have been addressed

**********

2. Is the manuscript technically sound, and do the data support the conclusions?

The manuscript must describe a technically sound piece of scientific research with data that supports the conclusions. Experiments must have been conducted rigorously, with appropriate controls, replication, and sample sizes. The conclusions must be drawn appropriately based on the data presented.

Reviewer #1: Yes

Reviewer #2: Yes

**********

3. Has the statistical analysis been performed appropriately and rigorously?

Reviewer #1: Yes

Reviewer #2: Yes

**********

4. Have the authors made all data underlying the findings in their manuscript fully available?

The PLOS Data policy requires authors to make all data underlying the findings described in their manuscript fully available without restriction, with rare exception (please refer to the Data Availability Statement in the manuscript PDF file). The data should be provided as part of the manuscript or its supporting information, or deposited to a public repository. For example, in addition to summary statistics, the data points behind means, medians and variance measures should be available. If there are restrictions on publicly sharing data—e.g. participant privacy or use of data from a third party—those must be specified.

Reviewer #1: (No Response)

Reviewer #2: (No Response)

**********

5. Is the manuscript presented in an intelligible fashion and written in standard English?

PLOS ONE does not copyedit accepted manuscripts, so the language in submitted articles must be clear, correct, and unambiguous. Any typographical or grammatical errors should be corrected at revision, so please note any specific errors here.

Reviewer #1: Yes

Reviewer #2: Yes

**********

6. Review Comments to the Author

Please use the space provided to explain your answers to the questions above. You may also include additional comments for the author, including concerns about dual publication, research ethics, or publication ethics. (Please upload your review as an attachment if it exceeds 20,000 characters)

Reviewer #1: (No Response)

Reviewer #2: Dear Authors,

The revised manuscript shows clear improvement in structure, clarity, and overall presentation. You have addressed many of the comments from the previous round, and several methodological and reporting aspects are now more transparent.

In particular terminology, conceptual clarity, better description of methodology, statistical analysis has been introduced etc. These changes have strengthened the manuscript.

However, a few points would still benefit from clarification:

1. Distinction between pesticide removal and degradation

The study refers to “degradation efficiency,” but the current design does not clearly distinguish between physical removal (transfer to washing solution) and chemical degradation. As residues in the rinse water were not measured, the reported effects may partly reflect removal rather than degradation. This should be more explicitly acknowledged as a limitation, or the interpretation adjusted accordingly.

2. Potential artefacts during extraction

The use of methanol and controlled temperature during extraction is noted. Given the presence of relatively long-lived reactive species, some continued degradation during ultrasonication cannot be fully excluded. A brief statement acknowledging this as a potential limitation would improve transparency.

3. Environmental relevance of pesticide concentration

Although the authors provide a justification for using a 2000 ppm stock solution and relate the resulting residue levels to SANTE guidelines, the explanation remains somewhat unclear and may be misleading for readers. A short note on how this setup relates to real-world conditions would strengthen the interpretation.

4. Statistical analysis

The inclusion of ANOVA is appropriate, however, a brief clarification of the statistical model, post-hoc testing (if applied), and number of replicates per treatment would improve reproducibility.

Overall comment

The manuscript has improved significantly and addresses a relevant topic. The remaining issues mainly concern clarification of interpretation and methodological transparency. Addressing these points would further strengthen the reliability of the conclusions.

**********

7. PLOS authors have the option to publish the peer review history of their article (what does this mean?). If published, this will include your full peer review and any attached files.

If you choose “no”, your identity will remain anonymous but your review may still be made public.

**Do you want your identity to be public for this peer review?**  For information about this choice, including consent withdrawal, please see our Privacy Policy.

Reviewer #1: No

Reviewer #2: No

**********

[NOTE: If reviewer comments were submitted as an attachment file, they will be attached to this email and accessible via the submission site. Please log into your account, locate the manuscript record, and check for the action link "View Attachments". If this link does not appear, there are no attachment files.]

To ensure your figures meet our technical requirements, please review our figure guidelines: https://journals.plos.org/plosone/s/figures

You may also use PLOS’s free figure tool, NAAS, to help you prepare publication quality figures: https://journals.plos.org/plosone/s/figures#loc-tools-for-figure-preparation.

NAAS will assess whether your figures meet our technical requirements by comparing each figure against our figure specifications.

# Author comment: Author response to Decision Letter 3

**DOI:** 10.1371/journal.pone.0351955.r006

20 Apr 2026

We sincerely thank the Editor and reviewers for their careful evaluation of our manuscript and for the constructive comments and suggestions. These insights were extremely helpful and have significantly improved the clarity, quality, and scientific rigor of the manuscript. All comments have been carefully considered and addressed in detail below.

Additional Editor Comments:

With the reviewer comments, the revised manuscript is improved compared to previous version. Though, the manuscript needs revision with respect to the reviewer comments and following comments.

Line 1-2: In the manuscript title, write like “……. on pesticide-spiked surfaces using ……” instead of “……… on pesticide-spiked produce surfaces using ………”.

Reply: Changed

Line 15: Write like “Pesticide-spiked food produce surfaces …..” instead of “Pesticide-spiked Produce surfaces …..”.

Reply: Wrote.

Line 20-21: Remove the SD values “(± 2.3–3.1%), (± 2.8–3.6%)” and “(mean ± SD, n = 3)”.

Reply: Removed

Please check the typographical and grammatical errors throughout the manuscript.

Reply: Checked carefully and corrected.

However, a few points would still benefit from clarification:

1. Distinction between pesticide removal and degradation

The study refers to “degradation efficiency,” but the current design does not clearly distinguish between physical removal (transfer to washing solution) and chemical degradation. As residues in the rinse water were not measured, the reported effects may partly reflect removal rather than degradation. This should be more explicitly acknowledged as a limitation, or the interpretation adjusted accordingly.

Reply: Thank you for this important observation. We agree that the current experimental design does not fully distinguish between physical removal (transfer into the washing solution) and actual chemical degradation. In our study, the reduction in pesticide residues on the sample surface may indeed result from a combination of both processes. As the pesticide content in the washing solution was not quantified, we cannot definitively separate these contributions. We have now explicitly acknowledged this as a limitation in the revised manuscript and adjusted the wording from “degradation efficiency” to “removal/degradation efficiency” and sometimes removal and/or degradation efficiency where appropriate to avoid overinterpretation. It was acknowledged in the limitation section line 447 to 449.

2. Potential artefacts during extraction

The use of methanol and controlled temperature during extraction is noted. Given the presence of relatively long-lived reactive species, some continued degradation during ultrasonication cannot be fully excluded. A brief statement acknowledging this as a potential limitation would improve transparency.

Reply: We appreciate this insightful comment. Although methanol extraction and controlled temperature conditions were employed to minimize post-treatment reactions, we acknowledge that the presence of relatively long-lived reactive species may lead to continued degradation during ultrasonication. This possibility has now been included as a limitation in the revised manuscript to ensure transparency. “Despite controlled extraction conditions, the possibility of continued degradation due to residual reactive species during ultrasonication cannot be completely excluded.” Is included in the materials and methods section line 195 to 197.

3. Environmental relevance of pesticide concentration

Although the authors provide a justification for using a 2000 ppm stock solution and relate the resulting residue levels to SANTE guidelines, the explanation remains somewhat unclear and may be misleading for readers. A short note on how this setup relates to real-world conditions would strengthen the interpretation.

Reply: Thank you for this valuable comment. We agree that clarification of the environmental relevance of the pesticide concentrations is important. The high concentration (2000 ppm) refers only to the stock solution used for accurate preparation of working solutions and does not represent the actual residue levels on the produce. The final spiking levels applied to the samples (0.1–0.5 µg/g) were selected in accordance with SANTE/11312/2021 guidelines and are within ranges reported for real-world residue levels. To avoid misunderstanding, we have added a clarifying statement in the Introduction section (Line 92 - 95) and in the limitation part line is polished (541-542).

4. Statistical analysis

The inclusion of ANOVA is appropriate, however, a brief clarification of the statistical model, post-hoc testing (if applied), and number of replicates per treatment would improve reproducibility.

Reply: Paragraph is modified to address the query, line 253-260.

Attachment Submitted filename: Response to Reviewer R3.docx

# Peer review: Decision Letter 3

**DOI:** 10.1371/journal.pone.0351955.r007

**Authors:** S Ezhil Vendan, S Ezhil Vendan, S Ezhil Vendan, S Ezhil Vendan

28 Apr 2026

PONE-D-25-44051R3

Removal Efficiency of Pesticide Residues on Pesticide-Spiked Perilla Leaf and Broccoli Surfaces Using Microplasma-Treated Water

PLOS One

Dear Dr. Khan,

Thank you for submitting your manuscript to PLOS ONE. After careful consideration, we feel that it has merit but does not fully meet PLOS ONE’s publication criteria as it currently stands. Therefore, we invite you to submit a revised version of the manuscript that addresses the points raised during the review process.

==============================

**ACADEMIC EDITOR:**

The manuscript needs revision with respect to the reviewer comments. Authors may revise the manuscript and submit the revised version with response to comments.

==============================

Please submit your revised manuscript by Jun 12 2026 11:59PM. If you will need more time than this to complete your revisions, please reply to this message or contact the journal office at plosone@plos.org. When you're ready to submit your revision, log on to https://www.editorialmanager.com/pone/ and select the 'Submissions Needing Revision' folder to locate your manuscript file.

Please include the following items when submitting your revised manuscript:

- A letter that responds to each point raised by the academic editor and reviewer(s). You should upload this letter as a separate file labeled 'Response to Reviewers'.
- A marked-up copy of your manuscript that highlights changes made to the original version. You should upload this as a separate file labeled 'Revised Manuscript with Track Changes'.
- An unmarked version of your revised paper without tracked changes. You should upload this as a separate file labeled 'Manuscript'.

If you would like to make changes to your financial disclosure, please include your updated statement in your cover letter. Guidelines for resubmitting your figure files are available below the reviewer comments at the end of this letter.

If applicable, we recommend that you deposit your laboratory protocols in protocols.io to enhance the reproducibility of your results. Protocols.io assigns your protocol its own identifier (DOI) so that it can be cited independently in the future. For instructions see: https://journals.plos.org/plosone/s/submission-guidelines#loc-laboratory-protocols. Additionally, PLOS ONE offers an option for publishing peer-reviewed Lab Protocol articles, which describe protocols hosted on protocols.io. Read more information on sharing protocols at https://plos.org/protocols?utm_medium=editorial-email&utm_source=authorletters&utm_campaign=protocols.

As the corresponding author, your ORCID iD is verified in the submission system and will appear in the published article. PLOS supports the use of ORCID, and we encourage all coauthors to register for an ORCID iD and use it as well. Please encourage your coauthors to verify their ORCID iD within the submission system before final acceptance, as unverified ORCID iDs will not appear in the published article. *Only*  the individual author can complete the verification step; PLOS staff *cannot*  verify ORCID iDs on behalf of authors.

We look forward to receiving your revised manuscript.

Kind regards,

S Ezhil Vendan, Ph.D

Academic Editor

PLOS One

Journal Requirements:

If the reviewer comments include a recommendation to cite specific previously published works, please review and evaluate these publications to determine whether they are relevant and should be cited. There is no requirement to cite these works unless the editor has indicated otherwise.

Please review your reference list to ensure that it is complete and correct. If you have cited papers that have been retracted, please include the rationale for doing so in the manuscript text, or remove these references and replace them with relevant current references. Any changes to the reference list should be mentioned in the rebuttal letter that accompanies your revised manuscript. If you need to cite a retracted article, indicate the article’s retracted status in the References list and also include a citation and full reference for the retraction notice.

Additional Editor Comments :

The manuscript needs revision with respect to the reviewer comments. Authors may revise the manuscript and submit the revised version with response to comments.

[Note: HTML markup is below. Please do not edit.]

Reviewers' comments:

Reviewer's Responses to Questions

**Comments to the Author**

1. If the authors have adequately addressed your comments raised in a previous round of review and you feel that this manuscript is now acceptable for publication, you may indicate that here to bypass the “Comments to the Author” section, enter your conflict of interest statement in the “Confidential to Editor” section, and submit your "Accept" recommendation.

Reviewer #2: All comments have been addressed

**********

2. Is the manuscript technically sound, and do the data support the conclusions?

The manuscript must describe a technically sound piece of scientific research with data that supports the conclusions. Experiments must have been conducted rigorously, with appropriate controls, replication, and sample sizes. The conclusions must be drawn appropriately based on the data presented.

Reviewer #2: Yes

**********

3. Has the statistical analysis been performed appropriately and rigorously?

Reviewer #2: Yes

**********

4. Have the authors made all data underlying the findings in their manuscript fully available?

The PLOS Data policy requires authors to make all data underlying the findings described in their manuscript fully available without restriction, with rare exception (please refer to the Data Availability Statement in the manuscript PDF file). The data should be provided as part of the manuscript or its supporting information, or deposited to a public repository. For example, in addition to summary statistics, the data points behind means, medians and variance measures should be available. If there are restrictions on publicly sharing data—e.g. participant privacy or use of data from a third party—those must be specified.

Reviewer #2: Yes

**********

5. Is the manuscript presented in an intelligible fashion and written in standard English?

PLOS ONE does not copyedit accepted manuscripts, so the language in submitted articles must be clear, correct, and unambiguous. Any typographical or grammatical errors should be corrected at revision, so please note any specific errors here.

Reviewer #2: Yes

**********

6. Review Comments to the Author

Please use the space provided to explain your answers to the questions above. You may also include additional comments for the author, including concerns about dual publication, research ethics, or publication ethics. (Please upload your review as an attachment if it exceeds 20,000 characters)

Reviewer #2: Dear authors,

the manuscript has improved substantially and now meets the general standards of PLOS ONE in terms of methodological transparency, structure, and clarity. The authors have addressed most of the previously raised concerns in a satisfactory manner.

Before final acceptance, I recommend one minor but important revision related to the interpretation of the results:

While the distinction between pesticide removal and degradation is now acknowledged and the terminology has been adjusted accordingly, some statements in the manuscript still imply effective or near-complete degradation in the washing system based on prior studies in standard solutions. In particular, assumptions regarding the absence of pesticide residues in the wash water are not directly supported by the experimental data presented in this study.

I recommend moderating the wording in the following instances to avoid overinterpretation:

Line 182: “The 4 min exposure … ensures that any pesticides transferred to the wash water are effectively degraded.”

The term “ensures” is too strong and not methodologically justified.

Suggested: “The 4 min exposure exceeds the degradation time observed in standard solutions; however, degradation of pesticides transferred to the wash water was not directly verified under the present experimental conditions.”

Line 184: “ultrasonication … is unlikely to result in additional degradation attributable to residual reactive nitrogen species (RNS)”

This statement is inconsistent with the acknowledged limitation.

Suggested: “Ultrasonication may still allow limited additional degradation due to residual reactive species, which cannot be fully excluded.”

Line 380: “comparable behavior was observed in real field samples, ensuring its effectiveness.”

“Ensuring” implies confirmation beyond the presented data.

Suggested: “Comparable trends were observed in produce samples, supporting the effectiveness of the treatment in reducing surface residues.”

Line 416: “Therefore, it may be reasonably assumed that the wash water did not contain significant amounts of undegraded pesticides.”

This is a direct assumption without experimental verification.

Suggested: “However, the presence of pesticide residues in the wash water was not quantified and cannot be excluded.”

Line 554: “MPW system performed well at removing and/or degrading pesticides…”

This still implies confirmed degradation.

Suggested: “MPW system performed well in reducing pesticide residues (through removal and/or degradation) on produce surfaces.”

This is a matter of wording and interpretation rather than additional experimentation, but it is important to ensure that the conclusions remain fully supported by the data.

With these minor clarifications, the manuscript would be suitable for publication.

With my best regards.

**********

7. PLOS authors have the option to publish the peer review history of their article (what does this mean?). If published, this will include your full peer review and any attached files.

If you choose “no”, your identity will remain anonymous but your review may still be made public.

**Do you want your identity to be public for this peer review?**  For information about this choice, including consent withdrawal, please see our Privacy Policy.

Reviewer #2: No

**********

[NOTE: If reviewer comments were submitted as an attachment file, they will be attached to this email and accessible via the submission site. Please log into your account, locate the manuscript record, and check for the action link "View Attachments". If this link does not appear, there are no attachment files.]

To ensure your figures meet our technical requirements, please review our figure guidelines: https://journals.plos.org/plosone/s/figures

You may also use PLOS’s free figure tool, NAAS, to help you prepare publication quality figures: https://journals.plos.org/plosone/s/figures#loc-tools-for-figure-preparation.

NAAS will assess whether your figures meet our technical requirements by comparing each figure against our figure specifications.

# Author comment: Author response to Decision Letter 4

**DOI:** 10.1371/journal.pone.0351955.r008

28 Apr 2026

We sincerely thank the reviewer for the thorough and highly constructive evaluation of our manuscript. We particularly appreciate the detailed and precise suggestions provided, which have been extremely helpful in improving the clarity, consistency, and scientific interpretation of our work. All comments have been carefully addressed, and the manuscript has been revised accordingly. We believe these revisions have strengthened the overall quality of the manuscript and ensured that all conclusions are fully supported by the experimental data.

6. Review Comments to the Author

Please use the space provided to explain your answers to the questions above. You may also include additional comments for the author, including concerns about dual publication, research ethics, or publication ethics. (Please upload your review as an attachment if it exceeds 20,000 characters)

Reviewer #2: Dear authors,

the manuscript has improved substantially and now meets the general standards of PLOS ONE in terms of methodological transparency, structure, and clarity. The authors have addressed most of the previously raised concerns in a satisfactory manner.

Before final acceptance, I recommend one minor but important revision related to the interpretation of the results:

While the distinction between pesticide removal and degradation is now acknowledged and the terminology has been adjusted accordingly, some statements in the manuscript still imply effective or near-complete degradation in the washing system based on prior studies in standard solutions. In particular, assumptions regarding the absence of pesticide residues in the wash water are not directly supported by the experimental data presented in this study.

I recommend moderating the wording in the following instances to avoid overinterpretation:

Line 182: “The 4 min exposure … ensures that any pesticides transferred to the wash water are effectively degraded.”

The term “ensures” is too strong and not methodologically justified.

Suggested: “The 4 min exposure exceeds the degradation time observed in standard solutions; however, degradation of pesticides transferred to the wash water was not directly verified under the present experimental conditions.”

Reply: Accepted the suggestion and modified accordingly.

Line 184: “ultrasonication … is unlikely to result in additional degradation attributable to residual reactive nitrogen species (RNS)”

This statement is inconsistent with the acknowledged limitation.

Suggested: “Ultrasonication may still allow limited additional degradation due to residual reactive species, which cannot be fully excluded.”

Reply: Accepted the suggestion and modified accordingly.

Line 380: “comparable behavior was observed in real field samples, ensuring its effectiveness.”

“Ensuring” implies confirmation beyond the presented data.

Suggested: “Comparable trends were observed in produce samples, supporting the effectiveness of the treatment in reducing surface residues.”

Reply: Accepted the suggestion and modified accordingly.

Line 416: “Therefore, it may be reasonably assumed that the wash water did not contain significant amounts of undegraded pesticides.”

This is a direct assumption without experimental verification.

Suggested: “However, the presence of pesticide residues in the wash water was not quantified and cannot be excluded.”

Reply: Accepted the suggestion and modified accordingly.

Line 554: “MPW system performed well at removing and/or degrading pesticides…”

This still implies confirmed degradation.

Suggested: “MPW system performed well in reducing pesticide residues (through removal and/or degradation) on produce surfaces.”

Reply: Accepted the suggestion and modified accordingly.

This is a matter of wording and interpretation rather than additional experimentation, but it is important to ensure that the conclusions remain fully supported by the data. With these minor clarifications, the manuscript would be suitable for publication. With my best regards.

Attachment Submitted filename: Response to Reviewer R4.docx

# Peer review: Decision Letter 4

**DOI:** 10.1371/journal.pone.0351955.r009

**Authors:** S Ezhil Vendan, S Ezhil Vendan, S Ezhil Vendan, S Ezhil Vendan, S Ezhil Vendan

4 Jun 2026

Removal Efficiency of Pesticide Residues on Pesticide-Spiked Perilla Leaf and Broccoli Surfaces Using Microplasma-Treated Water

PONE-D-25-44051R4

Dear Dr. Muhammad Saiful Islam Khan,

We’re pleased to inform you that your manuscript has been judged scientifically suitable for publication and will be formally accepted for publication once it meets all outstanding technical requirements.

Within one week, you’ll receive an e-mail detailing the required amendments. When these have been addressed, you’ll receive a formal acceptance letter and your manuscript will be scheduled for publication.

An invoice will be generated when your article is formally accepted. Please note, if your institution has a publishing partnership with PLOS and your article meets the relevant criteria, all or part of your publication costs will be covered. Please make sure your user information is up-to-date by logging into Editorial Manager at Editorial Manager® and clicking the ‘Update My Information' link at the top of the page. For questions related to billing, please contact billing support.

If your institution or institutions have a press office, please notify them about your upcoming paper to help maximize its impact. If they’ll be preparing press materials, please inform our press team as soon as possible -- no later than 48 hours after receiving the formal acceptance. Your manuscript will remain under strict press embargo until 2 pm Eastern Time on the date of publication. For more information, please contact onepress@plos.org.

Kind regards,

S Ezhil Vendan, Ph.D

Academic Editor

PLOS One

Additional Editor Comments (optional):

With the reviewers recommendation, the revised manuscript is satisfactory and accepted.

Reviewers' comments:

Reviewer's Responses to Questions

**Comments to the Author**

1. If the authors have adequately addressed your comments raised in a previous round of review and you feel that this manuscript is now acceptable for publication, you may indicate that here to bypass the “Comments to the Author” section, enter your conflict of interest statement in the “Confidential to Editor” section, and submit your "Accept" recommendation.

Reviewer #2: All comments have been addressed

**********

2. Is the manuscript technically sound, and do the data support the conclusions?

The manuscript must describe a technically sound piece of scientific research with data that supports the conclusions. Experiments must have been conducted rigorously, with appropriate controls, replication, and sample sizes. The conclusions must be drawn appropriately based on the data presented.

Reviewer #2: Yes

**********

3. Has the statistical analysis been performed appropriately and rigorously?

Reviewer #2: Yes

**********

4. Have the authors made all data underlying the findings in their manuscript fully available?

The PLOS Data policy requires authors to make all data underlying the findings described in their manuscript fully available without restriction, with rare exception (please refer to the Data Availability Statement in the manuscript PDF file). The data should be provided as part of the manuscript or its supporting information, or deposited to a public repository. For example, in addition to summary statistics, the data points behind means, medians and variance measures should be available. If there are restrictions on publicly sharing data—e.g. participant privacy or use of data from a third party—those must be specified.

Reviewer #2: Yes

**********

5. Is the manuscript presented in an intelligible fashion and written in standard English?

PLOS ONE does not copyedit accepted manuscripts, so the language in submitted articles must be clear, correct, and unambiguous. Any typographical or grammatical errors should be corrected at revision, so please note any specific errors here.

Reviewer #2: Yes

**********

6. Review Comments to the Author

Please use the space provided to explain your answers to the questions above. You may also include additional comments for the author, including concerns about dual publication, research ethics, or publication ethics. (Please upload your review as an attachment if it exceeds 20,000 characters)

Reviewer #2: Dear authors,

The manuscript has improved substantially and now meets the general standards of PLOS ONE in terms of clarity, structure, and methodological transparency.

Before final acceptance, I recommend a few changes focused on wording and interpretation:

While the distinction between pesticide removal and degradation is now acknowledged, some statements still imply confirmed or complete degradation based on prior studies in standard solutions. These interpretations are not fully supported by the current experimental design, as pesticide residues in the wash water were not directly measured.

I recommend slightly moderating such statements throughout the manuscript to avoid overinterpretation. In particular, please avoid terms such as “ensures” or assumptions about the absence of residues in the washing solution, and consistently frame the results as reduction of surface residues (removal and/or degradation) rather than confirmed degradation.

This is a minor issue of wording and does not require additional experiments.

With these small clarifications, the manuscript would be suitable for publication.

My best regards.

**********

7. PLOS authors have the option to publish the peer review history of their article (what does this mean?). If published, this will include your full peer review and any attached files.

If you choose “no”, your identity will remain anonymous but your review may still be made public.

**Do you want your identity to be public for this peer review?**  For information about this choice, including consent withdrawal, please see our Privacy Policy.

Reviewer #2: No

**********

# Editor report: Acceptance letter

**DOI:** 10.1371/journal.pone.0351955.r010

**Authors:** S Ezhil Vendan, S Ezhil Vendan, S Ezhil Vendan, S Ezhil Vendan, S Ezhil Vendan

PONE-D-25-44051R4

PLOS One

Dear Dr. Kim,

I'm pleased to inform you that your manuscript has been deemed suitable for publication in PLOS One. Congratulations! Your manuscript is now being handed over to our production team.

At this stage, our production department will prepare your paper for publication. This includes ensuring the following:

* All references, tables, and figures are properly cited

* All relevant supporting information is included in the manuscript submission,

* There are no issues that prevent the paper from being properly typeset

You will receive further instructions from the production team, including instructions on how to review your proof when it is ready. Please keep in mind that we are working through a large volume of accepted articles, so please give us a few days to review your paper and let you know the next and final steps.

Lastly, if your institution or institutions have a press office, please let them know about your upcoming paper now to help maximize its impact. If they'll be preparing press materials, please inform our press team within the next 48 hours. Your manuscript will remain under strict press embargo until 2 pm Eastern Time on the date of publication. For more information, please contact onepress@plos.org.

You will receive an invoice from PLOS for your publication fee after your manuscript has reached the completed accept phase. If you receive an email requesting payment before acceptance or for any other service, this may be a phishing scheme. Learn how to identify phishing emails and protect your accounts at https://explore.plos.org/phishing.

If we can help with anything else, please email us at customercare@plos.org.

Thank you for submitting your work to PLOS ONE and supporting open access.

Kind regards,

PLOS ONE Editorial Office Staff

on behalf of

Dr. S Ezhil Vendan

Academic Editor

PLOS One
